# Anomalous Spin‐Optical Helical Effect in Ti‐Based Kagome Metal

**DOI:** 10.1002/adma.202522533

**Published:** 2026-03-18

**Authors:** Federico Mazzola, Wojciech Brzezicki, Chiara Bigi, Armando Consiglio, Luciano Jacopo D'Onofrio, Maria Teresa Mercaldo, Adam Kłosiński, François Bertran, Patrick Le Fèvre, Oliver J. Clark, Mark T. Edmonds, Manuel Tuniz, Alessandro De Vita, Vincent Polewczyk, Jeppe B. Jacobsen, Henrik Jacobsen, Jill A. Miwa, Justin W. Wells, Anupam Jana, Ivana Vobornik, Jun Fujii, Niccolo Mignani, Narges Tarakameh Samani, Alberto Crepaldi, Giorgio Sangiovanni, Anshu Kataria, Tommaso Morresi, Samuele Sanna, Pietro Bonfá, Brenden R. Ortiz, Ganesh Pokharel, Stephen D. Wilson, Domenico Di Sante, Carmine Ortix, Mario Cuoco

**Affiliations:** ^1^ Department of Physics and Astronomy “Galileo Galilei” University of Padova Padova Italy; ^2^ CNR‐SPIN c/o Complesso di Monte S. Angelo Napoli Italy; ^3^ Institute of Theoretical Physics Jagiellonian University Kraków Poland; ^4^ International Research Centre MagTop, Institute of Physics Polish Academy of Sciences Warsaw Poland; ^5^ Synchrotron SOLEIL L'Orme des Merisiers Saint‐Aubin France; ^6^ Istituto Officina dei Materiali Consiglio Nazionale delle Ricerche Trieste Italy; ^7^ CNR‐SPIN c/o Universitá di Salerno Fisciano (SA) Italy; ^8^ Dipartimento di Fisica “E. R. Caianiello” Università di Salerno Fisciano (SA) Italy; ^9^ Institute of Theoretical Physics, Faculty of Physics University of Warsaw Warsaw Poland; ^10^ Univ Rennes IPR Institut de Physique de Rennes Rennes France; ^11^ School of Physics and Astronomy Monash University Clayton Australia; ^12^ Dipartimento di Fisica Universita degli studi di Trieste Trieste Italy; ^13^ Fritz Haber Institut der Max Planck Gesellshaft Berlin Germany; ^14^ Université Paris‐Saclay, UVSQ, CNRS, GEMaC Versailles France; ^15^ Nanoscience Center, Niels Bohr Institute University of Copenhagen Copenhagen Denmark; ^16^ European Spallation Source ERIC ‐ Data Management and Software Center Lyngby Denmark; ^17^ Department of Physics and Astronomy, Interdisciplinary Nanoscience Center Aarhus University Aarhus C Denmark; ^18^ Department of Physics and Centre for Materials Science and Nanotechnology University of Oslo (UiO) Oslo Norway; ^19^ CNR‐IOM Istituto Officina dei Materiali Trieste Italy; ^20^ Dipartimento di Fisica Politecnico di Milano Milano Italy; ^21^ Institute for Theoretical Physics and Astrophysics University of Würzburg Würzburg Germany; ^22^ Dipartimento di Scienze Matematiche, Fisiche e Informatiche Universitá di Parma Parma Italy; ^23^ European Centre for Theoretical Studies in Nuclear Physics and Related Areas (ECT*) Fondazione Bruno Kessler Trento Italy; ^24^ Department of Physics and Astronomy University of Bologna Bologna Italy; ^25^ Materials Science and Technology Division Oak Ridge National Laboratory Oak Ridge USA; ^26^ Materials Department University of California Santa Barbara Santa Barbara California USA; ^27^ Perry College of Mathematics, Computing, and Sciences University of West Georgia Carrollton USA

**Keywords:** quantum materials, loop currents, spin‐handedness, anomalous spin‐optical helical effect

## Abstract

The kagome lattice stands as a rich platform for hosting a wide array of correlated quantum phenomena, ranging from charge density waves and superconductivity to electron nematicity and loop current states. Direct detection of loop currents in kagome systems has remained a formidable challenge due to their intricate spatial arrangements and the weak magnetic field signatures they produce, and this has made their identification experimentally subtle. This has left their existence and underlying mechanisms a topic of intense debate. In this work, we uncover signatures compatible with loop currents: spin handedness‐selective signals that surpass conventional dichroic, spin, and spin‐dichroic responses. We observe this phenomenon in the kagome metal ${\mathrm{CsTi}}_{3}{\mathrm{Bi}}_{5}$ and we call it the anomalous spin‐optical helical effect. This effect arises from the coupling of light's helicity with spin‐orbital electron correlations, thereby providing an indirect yet sensitive approach to probe loop‐current–related electronic correlations in quantum materials. Our discovery not only enriches the debate surrounding loop currents but also offers new experimental strategies to exploit the electronic phases of quantum materials via light–matter interaction.

## Graphical abstract

The kagome lattice hosts diverse correlated quantum states, including elusive loop currents. We report spin‐handedness selective signals in CsTi_3_Bi_5_, termed the anomalous spin‐optical helical effect, surpassing conventional spin responses. Arising from light helicity coupled to spin‐orbital correlations, this effect provides a sensitive, indirect probe of loop‐currentrelated electronic states, offering new experimental strategies to explore and manipulate quantum phases in kagome metals.

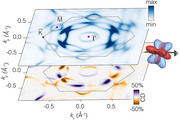

## 1 Introduction

Materials with kagome lattices provide an exceptional platform for exploring emergent correlated quantum phenomena, including charge density waves, superconductivity, and electronic nematicity [1, 2, 3, 4, 5, 6, 7, 8]. Kagome metals exhibit distinct electronic structure features, such as itinerant Dirac‐like states and flat bands, with established topologically nontrivial properties [9, 10, 11, 12, 13]. The “135” kagome family (${\mathrm{AB}}_{3}C_{5}$, with A: Rb, Cs, K; B: Ti, V; and C: Bi, Sb) has been extensively studied due to its rich variety of electronic correlated phases [14, 15, 16], alongside the intriguing potential for time‐reversal symmetry breaking induced by loop currents [17, 18, 19, 20, 21].

Loop current states, known for their violation of time‐reversal symmetry, are marked by electrons circulation along closed lattice paths, generating a magnetic flux. This flux, while intrinsic to the nature of these states, is difficult to detect due to its low amplitude and complex spatial distribution [22, 23, 24]. Despite extensive theoretical and experimental exploration, loop currents in kagome systems remain contentious regarding their origin and nature [20, 21, 25, 26]. These states are hypothesized to arise from bond‐order fluctuations [20] or symmetry reduction induced by charge density waves, evidenced by observations of chiral transport switchable with magnetic fields [19]. Recent neutron diffraction experiments by Liège et al. have detected signatures of loop currents in ${\mathrm{CsV}}_{3}{\mathrm{Sb}}_{5}$, suggesting electron circulation confined to vanadium sites [26]. However, weak signals raise questions about the authenticity of the observed time‐reversal symmetry breaking, underscoring the need for further investigation into the nature of these loop currents. The latter can exhibit identifiable signatures linked to the symmetry of spin and orbital patterns within the current flow but direct experimental confirmation has remained challenging.

When loop currents exhibit strong spin‐orbital correlations, they can induce an optical phenomenon involving the interaction between light helicity and electron spin. This effect, which we term spin‐orbital helical coupling, generates signals surpassing those of conventional spin, dichroic, spin, and spin‐dichroic responses, presenting a novel and promising method for detecting elusive quantum phases. In this work, motivated by a theory of spin‐orbital correlated loop current phases, we employ single‐handed polarized spin‐ and angle‐resolved photoelectron spectroscopy (spin‐ARPES) to reveal the existence of spin‐optical helical coupling in ${\mathrm{CsTi}}_{3}{\mathrm{Bi}}_{5}$. Our findings provide robust signatures which are compatible with time‐reversal symmetry breaking induced by loop currents marked by nonstandard spin‐orbital quadrupole correlations.

**FIGURE 1:**
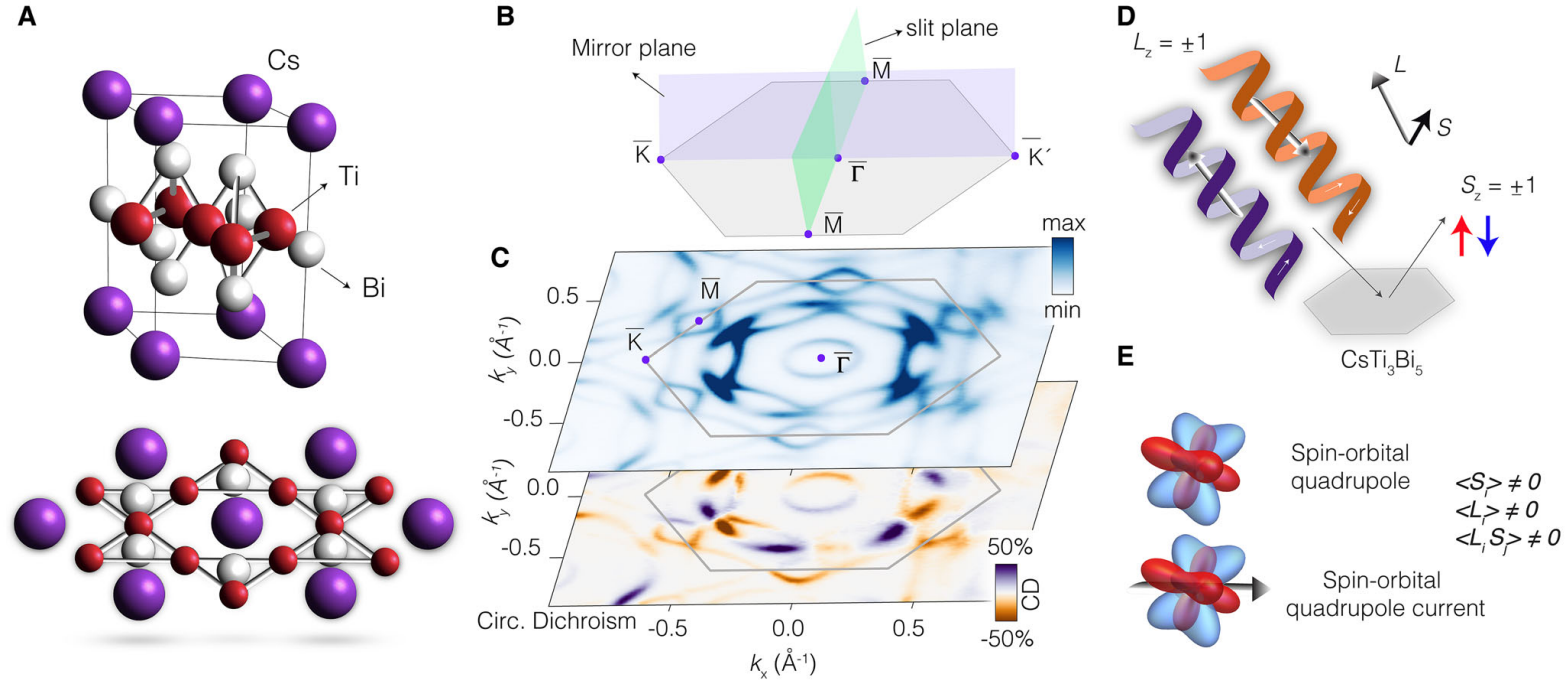
(a) Side view and top view crystal structure of ${\mathrm{CsTi}}_{3}{\mathrm{Bi}}_{5}$ showing the typical kagome geometry featured by the Ti atoms (red). (b) Experimental geometry: the incoming radiation comes within the mirror plane of the crystal and the measurements are collected orthogonal to it, along the plane of the analyzer's slit. (c) Fermi surface map of ${\mathrm{CsTi}}_{3}{\mathrm{Bi}}_{5}$ obtained by summing up circular right and left polarization (monochromatic color map) and subtracting them (purple‐orange color map). In the experimental geometry used, the circular dichroism signal is well‐defined. (d) Schematic of the spin‐resolved circularly polarized ARPES: the helicity of the light can couple to the orbital angular momentum and the spin of electrons. By using a spin‐detector, we can select the spin of the photoemitted electrons. (e) Example of spin‐orbital quadrupoles and of currents generated by them. The breaking of the relevant spin and orbital quantities is also depicted.

Single crystals of ${\mathrm{CsTi}}_{3}{\mathrm{Bi}}_{5}$ were grown using a flux‐based growth technique, as detailed in reference [27] and the methods section. The crystal structure, depicted in Figure 1a, is characteristic of the “135” kagome family, featuring Ti atoms (red) that form a frustrated kagome network, Bi atoms (white) residing within the same plane, and alkali Cs (purple) positioned above and below this plane. While ${\mathrm{CsTi}}_{3}{\mathrm{Bi}}_{5}$ shares several features with its V‐based sister compounds, it does not undergo a charge density wave transition, nor was superconductivity observed in our samples [28], contrary to what reported in a recent study [29]. ${\mathrm{CsTi}}_{3}{\mathrm{Bi}}_{5}$ provides a particularly clean platform to investigate the emergence of loop current states driven by spin‐orbital interactions. In contrast to related (Cs,Rb,K,)$V_{3}{\mathrm{Sb}}_{5}$ compounds, it does not undergo a charge density wave transition. This absence of translational symmetry breaking allows one to isolate electronic mechanisms from those potentially mediated by the lattice. While CDW‐induced symmetry breaking has been suggested as a route to time‐reversal symmetry breaking and loop current formation in the Sb‐based analogues, our focus here is to examine a complementary scenario, in which such effects originate from the strong spin‐orbit coupling associated with the heavy Bi atoms (see Supporting Information for details on spin‐orbit coupling and role of Bi).

The samples were cleaved under ultrahigh vacuum conditions ($1\times 10^{-10}$ mbar) and maintained at approximately 10 K throughout the measurements. This temperature represents an optimal compromise between minimising thermal broadening and enabling reliable alignment of the electronic states with respect to the analyser slit. We emphasise that the surface of ${\mathrm{CsTi}}_{3}{\mathrm{Bi}}_{5}$ is particularly fragile; even minor contamination induced by small thermal variations was sufficient to degrade the spectral quality. For this reason, we cleaved the samples at the same temperature of the data acquisition directly in the measurements chamber. All photoemission experiments were performed with synchrotron light directed at the sample's surfaces within one of the crystal's mirror plane (purple plane in Figure 1b). The analyzer's slit was oriented orthogonally to this plane (green shades in Figure 1b). This experimental configuration ensures that the geometrical contributions to the circular dichroism are well‐defined, yielding anti‐symmetric outcomes in a perfectly mirror and time‐reversal symmetric scenario.

**FIGURE 2:**
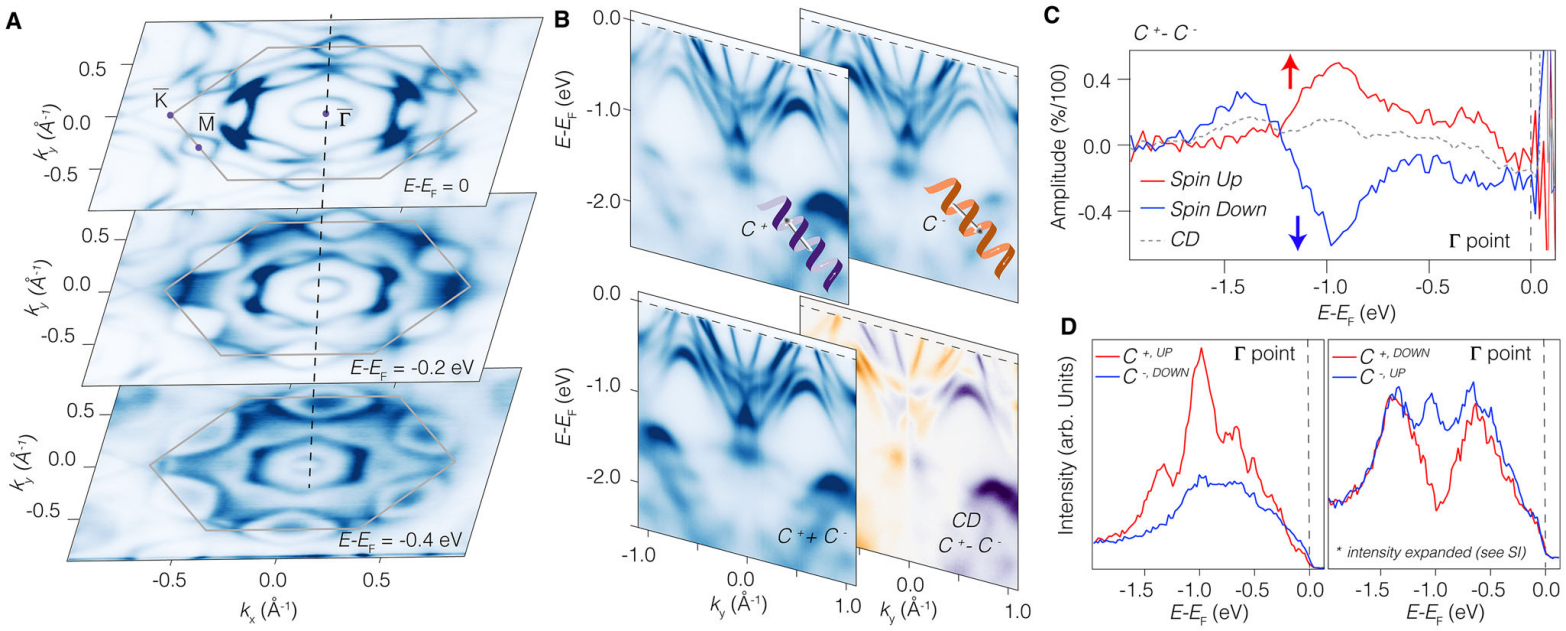
(a) Constant energy maps obtained by summing both circular right and left polarization. The kagome signatures are visible in reciprocal space, similar to other members of the “135” family. (b) Energy versus momenta maps showing the typical electronic structure of ${\mathrm{CsTi}}_{3}{\mathrm{Bi}}_{5}$ obtained with both circular right and left polarization (up), and by summing and subtracting them (down). (c) Spin‐resolved CP‐ARPES (up spin in red, down spin in blue) collected from the Γ point, together with spin‐integrated signal (gray). (d) Single handed polarization signals showing the breaking of time reversal symmetry.

Fermi surfaces collected with circularly polarized angle‐resolved photoelectron spectroscopy (CP‐ARPES), at photon energy where the spectral features were prominent and the circular dichroism from ARPES more symmetric (see also Supporting Information Figures S4– S11 for data collected with various photon energies and polarizations, including $k_{z}$ dispersions and constant energy contours). The measurements reveal the characteristic kagome geometry, featuring a circular pocket centered at $\bar{\Gamma }$, two hexagonal sheets rotated by 30 degrees relative to each other, triangular features around $\bar{K}$, and diamond‐shaped pockets centered at $\bar{M}$ (see Figures 1c and 2a for constant energy maps) [30, 31, 32, 33]. The observed circular dichroism of the Fermi surface, collected without spin‐detector, indicates that the system conserves overall time‐reversal and mirror symmetries (see Figure 1c). Along the $\bar{\Gamma }$‐$\bar{K}$ high‐symmetry direction, the circular dichroism is weak and negligible within the experimental resolution, specifically regarding the degree of asymmetry in light polarization (approximately 10%, see also Ref.[10]). Crucially, however, at the Γ point (k=0), the circular dichroism remains consistently close to zero (within 5%) across all photon energies spanning multiple Brillouin zones (See Supporting Information Figures 4– 6). Since the Daimon effect [34] vanishes in the limit of zero dichroism, we can attribute the observed nonzero signal in the L·S channel to an intrinsic effect rooted in initial‐state symmetry breaking (See extended discussion in the Supporting Information). This signal is much larger than the one collected for the spin‐polarization itself, making this case study different from what would be expected for a ferromagnetic or ferrimagnetic system. In addition, we anticipate that this experiment is performed exclusively for signals from the Brillouin zone center.

Recent studies have demonstrated that quantum systems with negligible circular dichroism can still exhibit non‐vanishing contributions in spin‐dichroic amplitudes, which can be linked to chirality and loop currents [35, 36]. For ${\mathrm{CsTi}}_{3}{\mathrm{Bi}}_{5}$, we have investigated such spin‐dichroic amplitudes, exploiting spin‐resolved CP‐ARPES. To ensure comparability with prior literature [10], we used a controlled experimental geometry with measurements from the Γ point using the spin‐detectors (See methods). At first glance, the system behaves similarly to previously reported findings for CoSn [37] and the “166” kagome family ${\mathrm{XV}}_{6}{\mathrm{Sn}}_{6}$ (X: Ho, Sc, Tb) [10]: the spin‐integrated CP‐ARPES from Γ, as anticipated, shows small deviations from zero. The spin‐resolved CP‐ARPES from the same point, with up (red curve in Figure 2c) and down (blue curve in Figure 2c) spins, exhibits overall opposite signs. This result indicates that, within the experimental uncertainty of the polarization of the incoming radiation, time‐reversal symmetry shows no significant anomalies. In earlier investigations, the sign‐reversal of the spin‐resolved CP‐ARPES was enforced by mirror and time‐reversal symmetry. This is reflected in the total dichroic response ($CD=C^{+}-C^{-}$), where $CD^{↑}$ transitions into $-CD^{↓}$ upon application of the time‐reversal operator (TR). This symmetry relation was also observed in single‐handed polarized measurements: $TR\left(C^{+,↑}\right)=C^{-,↓}$ and $TR\left(C^{+,↓}\right)=C^{-,↑}$. However, in the absence of symmetry constraints, the spin‐dichroic left and right amplitudes may be different. The breaking of such a symmetry constraint for single handed light measurements is the way spin‐optical helical coupling manifests.

The latter effect is indeed observed in ${\mathrm{CsTi}}_{3}{\mathrm{Bi}}_{5}$, exhibiting a remarkably large and time‐reversal symmetry broken response to single‐handed polarized light, as shown by the curves in Figure 2d. Such a significant signal indicates that the mirror symmetric character of electrons is broken and that substantial spin‐orbital correlations are present, thereby supporting the compatibility with loop currents of spin‐orbital nature, as will be demonstrated later. Importantly, as we show in Supporting Information Figures S11 and S12, this result is independent on the choice of background and normalization used. We stress that while the spin‐optical coupling with the single helicity of light is consistently realized, the large asymmetry observed at Γ serves as compelling evidence for strong spin‐orbital correlations that break time‐reversal symmetry. Theoretically, one can expect to observe such a symmetry breaking also in the spin signals, even if with amplitudes smaller than the spin‐orbital ones. Whilst the latter appear strong in our experiments and are evident along the full energy range collected for the energy distribution curves from Γ, the bare spin‐signal (circularly unpolarized data) was less clear, not conclusive and difficult to disentangle from other spurious effects (See Supporting Information Figure S13 and Discussion). Theoretically, while a ferromagnetic phase would allow the local dichroism, the spin‐polarization, and the spin‐dichroism to be all different from zero, here we reveal a different behavior with local non‐zero spin and spin‐dichroic channels but with conservation of the circular dichroism, a clear characteristic compatible with the emergence of loop currents.

We also consider whether the observed spin‐optical anomaly is accompanied by the occurrence of a sizable magnetic moment. This aspect has been explored by means of muon spin rotation and relaxation experiments that have been conducted on powdered samples of ${\mathrm{CsTi}}_{3}{\mathrm{Bi}}_{5}$ (see Supporting Information for details). The analysis of the spectra, along with first principles simulations to assess the muon localization in the crystal, indicates that no static magnetic fields larger than 0.25 mT can be present at the muon site. This would potentially correspond to a dipolar contribution from Ti atoms originating from a magnetic moment smaller than $2\times 10^{-3}$ $\mu_{B}$. We would like to point out that there is no contradiction between the negligible static magnetic signal in μSR and ARPES results. ARPES outcomes refer to states at the Brillouin zone center with no net spin or orbital moments, that yet shows sizable spin dichroism and unique spin responses. The μSR signal averages over the entire zone, where moments cancel out due to inversion symmetry. Spin‐orbital quadrupole loop currents moreover involve internal helicity, producing charge flows with opposite helicities that cancel net magnetic fields. Thus, the extremely weak μSR signals aligns with these internal structure of the spin‐orbital quadrupole loop configurations, which do not generate detectable static magnetism (see Supporting Information for details).

**FIGURE 3:**
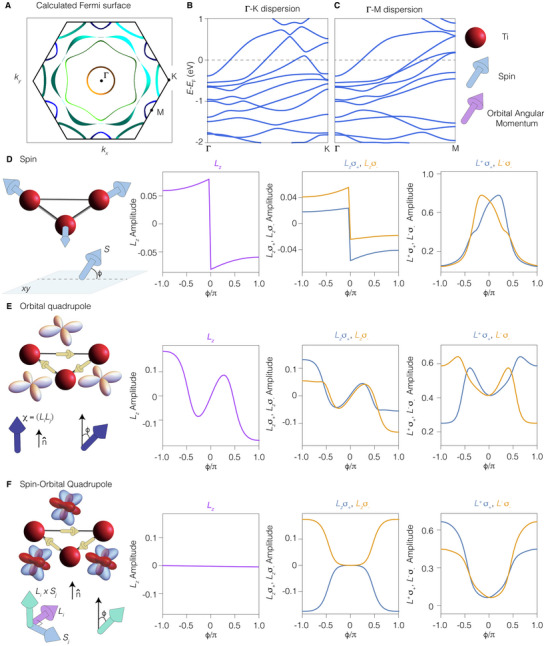
(a) Fermi surface of ${\mathrm{CsTi}}_{3}{\mathrm{Bi}}_{5}$ calculated via density functional theory. (b) Electronic dispersion along the Γ−K and (c) Γ−M directions of the Brillouin zone. (d) Schematic of magnetic ordering with spin moments forming 120‐degree angles in the plane; one spin orientation varies, introducing an out‐of‐plane component at angle ϕ. Orbital polarization ($L_{z}$), spin‐resolved projections ($L_{z}\sigma_{+}$, $L_{z}\sigma_{-}$), and left‐ ($L^{+}\sigma_{-}$) and right‐handed ($L^{-}\sigma_{+}$) amplitudes depend on ϕ. (e) Sketch of orbital quadrupole loop‐current in the kagome unit cell, with three mirror‐symmetric components parameterized by coupling χ. The polar angle ϕ influences the strength of $L_{x}L_{y}$ relative to $L_{x}L_{z}$ and $L_{y}L_{z}$. $L_{z}$ is nonzero only at isolated ϕ points, with differing magnitudes for spin‐resolved orbital polarizations. (f) Illustration of spin‐orbital quadrupole loop‐current with L×s correlations. The out‐of‐plane amplitude varies with ϕ, while planar components remain fixed. The response shows unique features: $L_{z}$ vanishes, and spin‐up and spin‐down dichroic components are equal but opposite, while left‐ and right‐handed orbital polarizations differ in magnitude.

We will now examine our experimental results from a theoretical perspective. We commence by analyzing the Fermi surface structure and the electronic dispersion near the Fermi level for the relevant bands investigated in the ARPES experiment. In Figure 3a, we present the Fermi surface obtained via density functional theory (DFT) (see Methods). The observed structure of the Fermi lines closely resembles the experimental observations (Figure 1c) regarding the number and character of the Fermi pockets around the high‐symmetry positions in the Brillouin zone. The shape and size of these structures correlate with the distinct band dispersions along the Γ−K (Figure 3b) and Γ−M (Figure 3c) directions, respectively. According to our DFT study, the electronic states near the Fermi level predominantly exhibit character stemming from the five d‐orbitals of titanium.

To analyze the dichroic and spin‐dichroic responses, we have developed a two‐dimensional tight‐binding model of the electronic structure of ${\mathrm{CsTi}}_{3}{\mathrm{Bi}}_{5}$, focusing on the complete multiplet of the Ti d‐orbitals ($d_{xy}$, $d_{yz}$, $d_{zx}$, $d_{x^{2}-y^{2}}$, $d_{3z^{2}-r^{2}}$). This allows us to effectively replicate the characteristics of the experimental Fermi surface. The electronic parameters were derived from Wannier projections of the DFT bands, utilizing the Ti d‐orbitals as the basis (see Methods).

Considering both spin and orbital degrees of freedom alongside the tri‐sublattice structure of the kagome lattice, we construct an effective model employing the orbital angular momentum L=2 for the five d‐orbital configurations at each Ti site, along with a $s=\frac{1}{2}$ moment for the electron spin. Additionally, we introduce a pseudospin T=1 to distinguish the states on the three inequivalent atoms within the unit cell. The Ti–Ti hybridization processes within the unit cell and between neighboring unit cells, as well as the local crystalline field potential, can be represented as a tensor product of the operators associated with the L, T, and s algebras (See Supporting Information). We then incorporate various types of symmetry‐breaking states to evaluate the dichroic, spin‐dichroic, and the spin‐resolved left‐ and right‐handed circularly polarized amplitudes of the photoemission process, specifically focusing on values of the crystal wave vector corresponding to the center of the Brillouin zone. The main aim here is to compare the response of different types of symmetry‐breaking states and to demonstrate that the observed experimental features can be uniquely associated with loop currents flowing within the unit cell and carrying a distinct type of spin‐orbital quadrupolar pattern. For each band eigenstate $|\psi_{n}\rangle$ evaluated at Γ, we determine the amplitude of the out‐of‐plane orbital moment $L_{z}=\langle \psi_{n}|{\hat{L}}_{z}\left|\psi_{n}\right\rangle$, the spin‐projected orbital moment $L_{z}\sigma_{\pm }=\langle \psi_{n}|\left(\frac{1}{2}\pm {\hat{s}}_{z}\right){\hat{L}}_{z}\left|\psi_{n}\right\rangle$ (i.e., the out‐of‐plane spin‐up (+) and down (−) components as selected by the projector $\left(\frac{1}{2}\pm {\hat{s}}_{z}\right)$), with ${\hat{s}}_{z}=\frac{1}{2}\sigma_{z}$, and the spin‐resolved left and right‐handed orbitally polarized amplitudes expressed as $L^{\pm }\sigma_{\pm }=\langle \psi_{n}|\left(\frac{1}{2}\pm {\hat{s}}_{z}\right){\hat{L}}_{z}\left(1\pm {\hat{L}}_{z}\right)\left(2\pm {\hat{L}}_{z}\right)\left|\psi_{n}\right\rangle$, respectively. For readability, we have omitted the band index n in the definitions of these observables. In a manner analogous to the spin projector, the operator ${\hat{L}}_{z}\left(1\pm {\hat{L}}_{z}\right)\left(2\pm {\hat{L}}_{z}\right)$ within the (L=2) manifold selects orbital configurations with left‐handed (+) or right‐handed (−) characteristics, dictated by the associated intrinsic orbital angular momentum. These observables are directly related to the dichroic ($L_{z}$), spin‐dichroic ($L_{z}\sigma_{\pm }$), and spin‐resolved left‐ and right‐handed ($L^{\pm }\sigma_{\pm }$) amplitudes probed by ARPES, respectively.

Before delving deeper into the behavior of these physical quantities, it is noteworthy that for electronic configurations exhibiting time‐reversal or mirror symmetry, the amplitudes $L_{z}$, $L_{z}\sigma_{\pm }$, and $L^{\pm }\sigma_{\pm }$ either vanish or are interconnected by symmetry conditions. This stems from the fact that the operators $\hat{L}$ and $\hat{s}$ change sign under time‐reversal symmetry transformation and behave as pseudovectors under mirror transformation. Therefore, in the presence of time and mirror symmetries, the electronic states probed at Γ are not anticipated to exhibit any distinctive anomalies in the dichroic, spin‐dichroic, or spin‐resolved amplitudes associated with left‐ and right‐handed circularly polarized light.

Next, we consider various magnetic configurations that break both time and mirror symmetries. We start with a standard magnetic ordering in which the spin moments are arranged to create 120‐degree angles in the plane, as illustrated schematically in Figure 3d. This magnetic configuration may arise due to the frustration inherent in the kagome lattice and results in the breaking of both time and mirror symmetries. We specifically allow one of the spin moments within the unit cell to exhibit an out‐of‐plane component, characterized by the angle ϕ. This broken symmetry state is introduced by an effective magnetic term in the Hamiltonian for the unit cell as given by $H_{\text{mag}}=J\left(s_{1}+s_{2}+s_{3}\right)\cdot \hat{\sigma }$ with $s_{1}=\left(\cos\phi ,\;0,\;\sin\phi \right)$, $s_{2}=\left(-\frac{1}{2},\;\frac{\sqrt{3}}{2},\;0\right)$, and $s_{3}=\left(-\frac{1}{2},\;-\frac{\sqrt{3}}{2},\;0\right)$, with J being the effective magnetic exchange amplitude. In the calculations, we set J=0.1eV, although the qualitative aspects of the results do not depend on the magnitude of J. Such configuration introduces a non‐vanishing scalar spin chirality, η, defined as the expectation value of $s_{1}\cdot \left(s_{2}\times s_{3}\right)$ (where labels 1, 2, and 3 correspond to the three titanium sites within the unit cell). When ϕ deviates from 0, η does not vanish, thereby breaking all mirror symmetries and any combinations of time and mirror symmetries. In this context, the variable ϕ is introduced to illustrate that the effects are not coincidental and do not arise solely in specific instances of broken symmetry states.

In Figure 3d, we investigate the behavior of the orbital polarization $L_{z}$ (left panel), the spin‐resolved components $\left(L_{z}\sigma_{+},L_{z}\sigma_{-}\right)$ (middle panel), and the spin‐resolved single‐handedness polarizations $\left(L^{+}\sigma_{-},L^{-}\sigma_{+}\right)$ (right panel) for a representative eigenstate $|\psi_{n}\rangle$ at Γ. We find that $L_{z}$ is generally non‐vanishing, except when considering states with spin moments lying entirely within the kagome plane. The amplitude of the spin‐resolved orbital polarization typically exhibits unequal magnitudes between the spin‐up and spin‐down channels, $\left(L_{z}\sigma_{+},L_{z}\sigma_{-}\right)$, a behavior that is also consistent for the spin‐resolved single‐handedness polarizations. This qualitative behavior is characteristic of all electronic states at Γ.

We will now examine two representative states of loop currents that demonstrate mirror‐broken orbital configurations (Figure 3e) and spin‐orbital quadrupoles (Figure 3f). Given that spin and orbital angular momentum possess pseudovector characteristics with magnetic dipole properties, their combination can be classified as a spin‐orbital quadrupole or an orbital quadrupole. Both spin and orbital angular momentum as well as the sublattice pseudospin are odd parity (i.e., they change sign) under time‐reversal transformation. Consequently, currents carrying spin‐orbital or orbital quadrupoles, which are even in time, violate time‐reversal symmetry.

The loop‐current state characterized by mirror‐broken orbital quadrupoles within the unit cell can be represented by introducing the vector field $\hat{\xi }=\left({\hat{\xi }}_{x},{\hat{\xi }}_{y},{\hat{\xi }}_{z}\right)$, where ${\hat{\xi }}_{x}={\hat{L}}_{y}{\hat{L}}_{z}+{\hat{L}}_{z}{\hat{L}}_{y}$, with other components derived through index permutation. The symmetry‐breaking orbital loop‐current term within the unit cell can then be generally expressed as ${\hat{J}}_{o}=\left({\hat{T}}_{x}+{\hat{T}}_{y}+{\hat{T}}_{z}\right)\left(\chi_{x}{\hat{\xi }}_{x}+\chi_{y}{\hat{\xi }}_{y}+\chi_{z}{\hat{\xi }}_{z}\right)$. The broken‐symmetry state is incorporated by adding to the Hamiltonian the term $H_{J_{o}}=g_{o}{\hat{J}}_{o}$, where $g_{o}$ denotes the amplitude of the symmetry‐breaking field. Such a contribution can be derived within a mean‐field decoupling of the nearest‐neighbor Coulomb interaction (see Supporting Information).

Analogous to the magnetic state with aligned spin moments, we analyze the dichroic, spin‐dichroic and single‐handedness responses for the loop‐current state exhibiting mirror‐broken orbital quadrupoles, as shown in Figure 3e. For clarity, we focus on a representative configuration that elucidates the overall behavior of the spin optical response. This is accomplished by maintaining the amplitudes of the (x,y) components, specifically setting $\chi_{x}=\chi_{y}=\chi_{0}$, while varying $\chi_{z}$ as $\chi_{z}=\chi_{0}\cdot \cos\left(\phi \right)$. The parameter ϕ serves to modulate the relative strength of the $\chi_{z}$ term with respect to the $\chi_{x}$ and $\chi_{y}$ components. The overall trend indicates that the orbital polarization typically does not vanish (left panel Figure 3e). Furthermore, the spin‐resolved responses lack any discernible symmetric behavior in amplitude or sign when comparing the spin‐up and spin‐down channels (middle panel Figure 3e) or the left and right orbitally polarized configurations (right panel Figure 3e). Therefore, both the magnetic state and the orbital‐quadrupole loop current phase display a response that contradicts the experimental findings, which show a nearly zero dichroic amplitude and a symmetric spin‐dichroic response.

Next, we consider the case of a loop‐current state featuring mirror‐ and rotation‐broken spin‐orbital quadrupole current (Figure 3f). The loop‐current state, characterized by a mirror‐broken spin‐orbital quadrupole within the unit cell, can be introduced by considering the vector field obtained from the cross product of the spin and orbital angular momentum, $\hat{L}\times \hat{s}$. Just like the orbital loop current, various couplings can be associated with different types of spin‐orbital quadrupolar distributions. The symmetry breaking spin‐orbital loop‐current term, within the unit cell, can be then expressed as ${\hat{J}}_{so}=g_{so}\left({\hat{T}}_{x}+{\hat{T}}_{y}+{\hat{T}}_{z}\right)\left[n\cdot \left(\hat{L}\times \hat{s}\right)\right]$ with $n=(n_{x},n_{y},n_{z})$ setting the director for the spin‐orbital quadrupole distribution, and $g_{so}$ the strength of the loop‐current state. The variation of the spin‐orbital loop current is made through the angle ϕ, i.e., the angle between the vector n and the cross product of $\hat{L}$ and $\hat{s}$. Similarly to the orbital quadrupole loop‐current phase, the spin–orbital quadrupole current configuration can be described by introducing into the Hamiltonian a symmetry‐breaking field of the form $H_{J_{so}}=g_{so}{\hat{J}}_{so}$. This type of loop‐current configuration leads to electronic states that exhibit a distinct response if compared to the spin aligned magnetic state or the orbital quadrupole loop‐current: the orbital polarization $L_{z}$ is zero (left panel Figure 3f) for all values of ϕ, the orbital moment for spin‐up and spin‐down configurations $\left(L_{z}\sigma_{+},L_{z}\sigma_{-}\right)$ have equal magnitudes but opposite signs (middle panel Figure 3f), and in contrast, the handedness and spin‐resolved orbital configurations display a profile with amplitudes that are unrelated in size or sign at any angle ϕ that characterizes the spin‐orbital loop current state (right panel Figure 3f). We would like to point out that this qualitative profile is representative of all the electronic states at Γ, although the distribution of amplitudes changes with energy. These distinctive features characterize the spin‐orbital loop current states, which are not typically found in other electronic phases that break time or mirror symmetries. Notably, the behavior of these loop current states, despite violating both mirror and time‐reversal symmetries, exhibits vanishing dichroism and symmetric amplitudes in the spin‐dichroic channel, traits that are usually associated with systems that uphold time‐reversal or mirror symmetries. Hence, the dichroic, the spin‐dichroic and the handedness responses for this loop current phase match very well with the ARPES experimental observations. We point out that a relevant aspect for the emergence of loop current phases with spin‐orbital cross correlations is in the presence of heavy Bi atoms in ${\mathrm{CsTi}}_{3}{\mathrm{Bi}}_{5}$. Indeed, we find that the electronic hybridization between nearest neighbor Ti sites through the Bi atom within the unit cell has a spin‐orbital structure that couples the spin and orbital moments in a transverse mode, that is there are terms of the type $s_{i}L_{k}$ with i different from k (see Supporting Information). This electronic hybridization is directly related to the presence of a strong spin‐orbit interaction at the Bi site and to the directionality of the Ti‐Bi bond. We point out that these contributions do not generate additional band splittings also due to the preserved inversion symmetry. Moreover, since their magnitude is smaller than the crystal‐field splitting, their effect is limited to a weak renormalization of the energy levels at the Brillouin–zone center.

While a surface‐localized contribution cannot be entirely excluded, several key observations constrain this scenario. The measurements are performed at the Γ point, where time‐reversal and crystal symmetries forbid net orbital polarization, and the total dichroic signal vanishes–ruling out mechanisms such as surface‐induced symmetry breaking or orbital‐Rashba effects, which would yield a finite signal. Any surface‐related contribution would thus appear as a uniform background, not as the structured spin‐helical texture we observe, supporting a bulk‐origin linked to intrinsic spin‐orbital correlations. Spin‐integrated ARPES measurements as a function of photon energy were performed using both right‐ and left‐circularly polarized light (see Figure S4, Supporting Information). The resulting spectra display negligible dispersion along the out‐of‐plane direction, consistent with a predominantly 2D character of the electronic wave functions, which makes it experimentally difficult to disentangle bulk‐derived states from surface‐related contributions. Nonetheless, the small circular dichroism detected at the centre of the Brillouin zone–remaining below 7% across all photon energies–indicates that the measurements are well outside the regime where the Daimon effect could dominate. Although the absence of spin‐ and circular‐dichroic measurements over the full photon‐energy range precludes a definitive depth‐resolved analysis, and a surface‐related contribution to the observed spin–optical helical response cannot be excluded, the effect would remain equally meaningful even if confined to the surface, as it still reflects intrinsic spin–orbital correlations compatible with the proposed loop‐current scenario. Our results therefore remain fully consistent with a spin–orbital correlated origin and motivate further investigations to determine whether this phenomenon is of bulk or surface character.

We would like to point out the implications of having domains with opposite chirality and how this affects the cancellation of the observed anomaly in spin‐resolved dichroic signals within spatially unresolved experiments. As discussed in detail in the supplementary information, the proposed loop currents–arising from spin‐orbital quadrupolar degrees of freedom–lead to a multitude of degenerate domains with opposite chirality. This degeneracy can prevent complete cancellation of the observed anomaly. Specifically, if the symmetry‐broken state is associated with a quadrupole loop current characterized by a particular vector n, there exist 23 symmetry‐related configurations obtained through mirror reflections and threefold rotations, all degenerate in energy. A unique aspect of this degenerate manifold is that these configurations can feature a response in the spin‐resolved single‐handedness polarizations, which can either be interchanged or have identical magnitudes. This behavior depends on whether the symmetry operations relating the spin‐orbital quadrupoles ‐ encoded in the n director ‐ involve combinations of mirror and time‐reversal transformations. Consequently, if the system forms domains with loop currents that possess distinct symmetry‐related spin‐orbital quadrupole configurations, the asymmetric amplitude observed in the spin‐resolved single‐handedness polarizations may not cancel out. To explore this further, we analyzed the energy landscape of domains with different loop current configurations (see Supporting Information for details about the ground state phase diagram). Our findings suggest that domains with opposite chirality can exhibit inequivalent spin‐orbital quadrupole distributions. Notably, domains with loop currents of differing chirality–due to mirror‐transformed directors–are energetically favorable. Importantly, these domains do not lead to cancelation of the asymmetric amplitudes in the spin‐resolved single‐handedness signals. Furthermore, spin‐orbital quadrupole loop current states generate weak magnetic dipolar fields, as demonstrated by muons, making long‐range magnetic dipolar interactions negligible. Domain sizes are determined by the balance between long‐range dipolar forces, which favor small domains, and short‐range interface energies that promote larger domains. Consequently, a few large domains spanning microns are expected. Due to the high degeneracy of loop current states, many small domains are unlikely. Thus, large domains' signals mainly reflect spin‐orbital quadrupole anisotropy, explaining persistent unidirectional handedness and spin‐resolved observations. We have investigated the temperature dependence of the spin–orbital quadrupolar loop‐current phase and find that thermal fluctuations suppress the order through a second‐order phase transition. Using a mean‐field decoupling of the nearest‐neighbor Coulomb interaction (see Supporting Information), we find that the ordering temperature can exceed room temperature even for relatively weak interactions. This robustness arises because the energy gain of the spin–orbital quadrupole state is controlled by the large crystalline‐field splitting (hundreds of meV) and sizable orbital‐dependent Ti–Ti hopping amplitudes. In contrast, conventional loop‐current phases without spin or orbital quadrupoles have transition temperatures set solely by the hopping scale t, with mean‐field estimates typically $T_{c}∼0.1\;t$ (100–500 K) [38, 39, 40, 41].

In conclusion, we have experimentally observed an anomalous spin‐optical helical effect in the “135” kagome system ${\mathrm{CsTi}}_{3}{\mathrm{Bi}}_{5}$, which is consistent with the presence of a loop current phase marked by robust spin‐orbital correlations. Our findings reveal this effect through signals from the Γ point, which we interpret as arising from spin‐orbital quadrupoles circulating across the central hexagonal lattice of the kagome structure, thereby providing a perspective that complements and informs prevailing theories regarding bond order in the literature [20]. Moreover, in the absence of charge density waves, the established loop currents are likely to reflect intrinsic features of the frustrated atomic network. We would like to also point out that, although the material system can exhibit nematic properties [29, 42, 43], these are unrelated to the observed effect, which arises at the Brillouin‐zone center and requires broken time‐reversal symmetry.

In addition, the effect observed, analogously to previous studies [35], could also appear within the first layers of the materials, making this approach a promising and versatile tool for exploring this phenomenology. Such explorations could deepen our understanding of the intricate interplay between atomic arrangements, electronic structures, and spin‐orbital correlations, with potential implications for the exploitation of novel quantum phases in technologies based on spin‐optical helical effects.

## 2 Methods

High‐purity single crystals of ${\mathrm{CsTi}}_{3}{\mathrm{Bi}}_{5}$ were synthesized using a conventional flux‐based method, following the procedure detailed in [27]. The starting materials included cesium (liquid, Alfa 99.98 %), titanium (powder, Alfa 99.9 %), and bismuth (shot, Alfa 99.999 %), which were combined in a stoichiometric ratio of 1:1:6. This mixture was placed in a tungsten carbide milling vial and subjected to one hour of milling in an argon atmosphere to produce the precursor powder. The powder was then transferred to an alumina crucible, which was sealed in a stainless‐steel tube. The sealed tube was heated to 900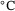 for 10 hours, followed by slow cooling at a rate of 3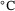 per hour until reaching 500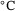. After the process, the tube was opened in an argon‐filled glove box, where the shiny, plate‐like single crystals were carefully separated and stored under an inert gas atmosphere.

The circularly polarized spin‐ARPES measurements were performed with light incident within one of the sample's mirror planes. This critical choice enables precise control over experimental conditions, allowing for effective probing of geometrical matrix elements. We collected energy‐distribution curves from the Γ point, where the geometrical matrix elements from the circular dichroism are defined to be zero. Subsequently, we obtained energy versus momentum maps using a spin detector, akin to the ARPES results reported in Figure 2b for spin‐integrated circular dichroism (see supplementary information figure 10). After aligning the crystals based on these maps, we extracted the energy distribution curve resolved in spin and circular polarization from the Γ point, as illustrated in Figure 2c.

Electronic structure calculations were performed using the full‐potential local‐orbital (FPLO) code (v.21.00‐61) [44]. The unit cell has lattice constants of 5.82709 Å, 5.82709 Å, and 9.93612 Å. The exchange‐correlation energy was parametrized within the local density approximation, following the Perdew‐Wang 92 formulation [45]. A 12×12×12 k‐grid was used to sample the Brillouin Zone, and the tetrahedron method was employed for integration. Calculations were performed in both the full‐relativistic and non‐relativistic frameworks.

A subsequent Wannier function model [46, 47], with symmetries accurately implemented, was constructed, considering projections onto the following states: Caesium 6s; Titanium $3d_{z^{2}}$, $3d_{xz}$, $3d_{yz}$, $3d_{x^{2}-y^{2}}$, $3d_{xy}$; and Bismuth $6p_{z}$, $6p_{x}$, $6p_{y}$, 6s. The Wannier functions are built using the projection method implemented in the FPLO code [48, 49]. In this approach, Wannier functions are obtained by projecting the Kohn–Sham Bloch states onto a set of localized atomic‐like orbitals within the selected energy window. This method provides localized Wannier functions that respect the crystal symmetries by construction, without performing the iterative minimization of the spread functional used in the maximally localized Wannier function (MLWF) scheme á la wannier90 [50, 51]. Although the initial Wannier projection included Ti 3d, Bi 6p/6s, and Cs 6s orbitals, the bands located near the Fermi level are dominated by Ti 3d character (see supplementary information). Contributions from Bi and Cs orbitals are also present, but they provide smaller spectral weights on the main kagome features of interest. Therefore, the effective tight‐binding model retains only the dominant Ti 3d Wannier functions. Hopping parameters involving Bi and Cs orbitals are negligible in the low‐energy window (see supplementary information for details).

## Author Contributions

F.M., W. B., and M. C. devised the project with the help of all the authors. The theoretical analysis has been developed by W.B., M.C., D.D.S., A.K., L.J.D'O., M.T.M., C.O and G.S¨ The samples were grown by S. W. and G. P¨ F. M., C. B., A. D. V. and M. T. performed the measurements. F.M. and M.C. wrote the manuscript, with input and help from all authors. All the authors discussed the results and their interpretation and revised the manuscript.

## Conflicts of Interest

The authors declare no conflict of interest.

## Supporting information


**Supporting File**: adma72799‐sup‐0001‐SuppMat.pdf.

## Data Availability

The data will be available upon request.

## References

[1] X. W. Yi , Z. W. Liao , J. Y. You , B. Gu , and G. Su , “Superconducting, Topological, and Transport Properties of Kagome Metals ${\mathrm{CsTi}}_{3}{\mathrm{Bi}}_{5}$ and ${\mathrm{RbTi}}_{3}{\mathrm{Bi}}_{5}$ ,” Research 6 (2023): 0238, https://spj.science.org/doi/abs/10.34133/research.0238. 37789987 10.34133/research.0238 PMC10543885

[2] Y. X. Jiang , S. Shao , W. Xia , et al., “Van Hove Annihilation and Nematic Instability on a Kagome Lattice,” Nature Materials 23, no. 9 (Sep 2024): 1214–1221, 10.1038/s41563-024-01914-z. 39009656

[3] L. Nie , K. Sun , W. Ma , et al., “Charge‐Density‐Wave‐Driven Electronic Nematicity in a Kagome Superconductor,” Nature 604, no. 7904 (Apr 2022): 59–64, 10.1038/s41586-022-04493-8. 35139530

[4] Y. Hu , C. Le , Y. Zhang , et al., “Non‐Trivial Band Topology and Orbital‐Selective Electronic Nematicity in a Titanium‐Based Kagome Superconductor,” Nature Physics 19, no. 12 (Dec 2023): 1827–1833, 10.1038/s41567-023-02215-z.

[5] T. Schwemmer , H. Hohmann , M. Dürrnagel , et al., “Sublattice Modulated Superconductivity in the Kagome Hubbard Model,” Physical Review B 110 (Jul 2024): 024501, https://link.aps.org/doi/10.1103/PhysRevB.110.024501.

[6] T. Neupert , M. M. Denner , J. X. Yin , R. Thomale , and M. Z. Hasan , “Charge Order and Superconductivity in Kagome Materials,” Nature Physics 18, no. 2 (Feb 2022): 137–143, 10.1038/s41567-021-01404-y.

[7] M. Kang , S. Fang , J. K. Kim , et al., “Twofold Van Hove Singularity and Origin of Charge Order in Topological Kagome Superconductor ${\mathrm{CsV}}_{3}{\mathrm{Sb}}_{5}$ ,” Nature Physics 18, no. 3 (Mar 2022): 301–308, 10.1038/s41567-021-01451-5.

[8] M. Kang , S. Fang , J. Yoo , et al., “Charge Order Landscape and Competition With Superconductivity in Kagome Metals,” Nature Materials 22, no. 2 (2023): 186–193, 10.1038/s41563-022-01375-2. 36329264

[9] M. Kang , S. Fang , L. Ye , et al., “Topological Flat Bands in Frustrated Kagome Lattice CoSn,” Nature Communications 11, no. 1 (2020): 4004, 10.1038/s41467-020-17465-1. PMC7417556 32778669

[10] D. Di Sante , C. Bigi , P. Eck , et al., “Flat Band Separation and Robust Spin Berry Curvature in Bilayer Kagome Metals,” Nature Physics 19, no. 8 (2023): 1135–1142, 10.1038/s41567-023-02053-z.

[11] M. Tuniz , A. Consiglio , D. Puntel , et al., “Dynamics and Resilience of the Unconventional Charge Density Wave in ${\mathrm{ScV}}_{6}{\mathrm{Sn}}_{6}$ Bilayer Kagome Metal,” Communications Materials 4, no. 1 (2023): 103, 10.1038/s43246-023-00430-y.

[12] M. Li , Q. Wang , G. Wang , et al., “Dirac Cone, Flat Band and Saddle Point in Kagome Magnet ${\mathrm{YMn}}_{6}{\mathrm{Sn}}_{6}$ ,” Nature Communications 12, no. 1 (2021): 3129, 10.1038/s41467-021-23536-8. PMC8149840 34035305

[13] Z. Liu , M. Li , Q. Wang , et al., “Orbital‐Selective Dirac Fermions and Extremely Flat Bands in Frustrated Kagome‐Lattice Metal CoSn,” Nature Communications 11, no. 1 (2020): 4002, 10.1038/s41467-020-17462-4. PMC7417585 32778641

[14] Y. Hu , X. Wu , B. R. Ortiz , S. Ju , et al., “Rich Nature of Van Hove Singularities in Kagome Superconductor ${\mathrm{CsV}}_{3}{\mathrm{Sb}}_{5}$ ,” Nature Communications 13, no. 1 (2022): 2220, 10.1038/s41467-022-29828-x. PMC9038924 35468883

[15] H. Zhao , H. Li , B. R. Ortiz , et al., “Cascade of Correlated Electron States in the Kagome Superconductor ${\mathrm{CsV}}_{3}{\mathrm{Sb}}_{5}$ ,” Nature 599, no. 7884 (2021): 216–221, 10.1038/s41586-021-03946-w. 34587622

[16] L. Zheng , Z. Wu , Y. Yang , et al., “Emergent Charge Order in Pressurized Kagome Superconductor ${\mathrm{CsV}}_{3}{\mathrm{Sb}}_{5}$ ,” Nature 611, no. 7937 (2022): 682–687, 10.1038/s41586-022-05351-3. 36418450

[17] T. Le , Z. Pan , Z. Xu , et al., “Superconducting Diode Effect and Interference Patterns in Kagome ${\mathrm{CsV}}_{3}{\mathrm{Sb}}_{5}$ ,” Nature 630, no. 8015 (2024): 64–69, 10.1038/s41586-024-07431-y. 38750364

[18] C. Guo , G. Wagner , C. Putzke , et al., “Correlated Order at the Tipping Point in the Kagome Metal ${\mathrm{CsV}}_{3}{\mathrm{Sb}}_{5}$ ,” Nature Physics 20, no. 4 (Apr 2024): 579–584, 10.1038/s41567-023-02374-z. 38638456 PMC11021193

[19] C. Guo , C. Putzke , S. Konyzheva , et al., “Switchable Chiral Transport in Charge‐Ordered Kagome Metal ${\mathrm{CsV}}_{3}{\mathrm{Sb}}_{5}$ ,” Nature 611, no. 7936 (2022): 461–466, 10.1038/s41586-022-05127-9. 36224393 PMC9668744

[20] R. Tazai , Y. Yamakawa , and H. Kontani , “Charge‐Loop Current Order and $Z_{3}$ Nematicity Mediated by Bond Order Fluctuations in Kagome Metals,” Nature Communications 14, no. 1 (2023): 7845, 10.1038/s41467-023-42952-6. PMC10687221 38030600

[21] M. H. Christensen , T. Birol , B. M. Andersen , and R. M. Fernandes , “Loop Currents in $AV_{3}{\mathrm{Sb}}_{5}$ Kagome Metals: Multipolar and Toroidal Magnetic Orders,” Physical Review B 106 (Oct 2022): 144504, https://link.aps.org/doi/10.1103/PhysRevB.106.144504.

[22] D. Bounoua , L. Mangin‐Thro , J. Jeong , et al., “Loop Currents in Two‐Leg Ladder Cuprates,” Communications Physics 3, no. 1 (2020): 123, 10.1038/s42005-020-0388-1.

[23] D. Bounoua , Y. Sidis , T. Loew , et al., “Hidden Magnetic Texture in the Pseudogap Phase of High‐$T_{c}$ ${\mathrm{YBa}}_{2}{\mathrm{Cu}}_{3}O_{6.6}$ ,” Communications Physics 5, no. 1 (2022): 268, 10.1038/s42005-022-01048-1.

[24] Y. Tang , L. Mangin‐Thro , A. Wildes , et al., “Orientation of the Intra‐Unit‐Cell Magnetic Moment in the High‐$T_{c}$ Superconductor ${\mathrm{HgBa}}_{2}{\mathrm{CuO}}_{4+\delta }$ ,” Physical Review B 98 (Dec 2018): 214418, https://link.aps.org/doi/10.1103/PhysRevB.98.214418.

[25] J. W. Dong , Z. Wang , and S. Zhou , “Loop‐Current Charge Density Wave Driven by Long‐Range Coulomb Repulsion on the Kagomé Lattice,” Physical Review B 107 (Jan 2023): 045127, https://link.aps.org/doi/10.1103/PhysRevB.107.045127.

[26] W. Liège , Y. Xie , D. Bounoua , et al., “Search for Orbital Magnetism in the Kagome Superconductor ${\mathrm{CsV}}_{3}{\mathrm{Sb}}_{5}$ Using Neutron Diffraction,” 2024, https://arxiv.org/abs/2407.14391.

[27] Zeitschrift für Naturforschung B 77, no. 11‐12 (2022), 10.1515/znb-2022-frontmatter11-12.

[28] M. Wenzel , E. Uykur , A. A. Tsirlin , et al., “Interplay of d‐ and p‐States in ${\mathrm{RbTi}}_{3}{\mathrm{Bi}}_{5}$ and ${\mathrm{CsTi}}_{3}{\mathrm{Bi}}_{5}$ Flat‐Band Kagome Metals,” (2025), https://arxiv.org/abs/2501.18389.

[29] H. Yang , Y. Ye , Z. Zhao , et al., “Superconductivity and Nematic Order in a New Titanium‐Based Kagome Metal ${\mathrm{CsTi}}_{3}{\mathrm{Bi}}_{5}$ Without Charge Density Wave Order,” Nature Communications 15 (Nov 2024): 9626, 10.1038/s41467-024-53870-6. PMC11543671 39511208

[30] J. Yang , X. Yi , Z. Zhao , et al., “Observation of Flat Band, Dirac Nodal Lines and Topological Surface States in Kagome Superconductor ${\mathrm{CsTi}}_{3}{\mathrm{Bi}}_{5}$ ,” Nature Communications 14, no. 1 (2023): 4089, 10.1038/s41467-023-39620-0. PMC10333313 37429852

[31] Y. Wang , Y. Liu , Z. Hao , et al., “Flat Band and $Z_{2}$ Topology of Kagome Metal ${\mathrm{CsTi}}_{3}{\mathrm{Bi}}_{5}$ ,” Chinese Physics Letters 40, no. 3 (Mar 2023): 037102, 10.1088/0256-307X/40/3/037102.

[32] B. Liu , M. Q. Kuang , Y. Luo , et al., “Tunable Van Hove Singularity Without Structural Instability in Kagome Metal ${\mathrm{CsTi}}_{3}{\mathrm{Bi}}_{5}$ ,” Physical Review Letters 131 (Jul 2023): 026701, https://link.aps.org/doi/10.1103/PhysRevLett.131.026701. 37505968 10.1103/PhysRevLett.131.026701

[33] Z. Jiang , Z. Liu , H. Ma , et al., “Flat Bands, Non‐Trivial Band Topology and Rotation Symmetry Breaking in Layered Kagome‐Lattice ${\mathrm{RbTi}}_{3}{\mathrm{Bi}}_{5}$ ,” Nature Communications 14, no. 1 (2023): 4892, 10.1038/s41467-023-40515-3. PMC10425367 37580381

[34] H. Boban , M. Qahosh , X. Hou , et al., “Scattering Makes a Difference in Circular Dichroic Angle‐Resolved Photoemission,” (2024), https://arxiv.org/abs/2410.19652.

[35] F. Mazzola , W. Brzezicki , M. T. Mercaldo , et al., “Signatures of a Surface Spin–Orbital Chiral Metal,” Nature 626, no. 8000 (2024): 752–758, 10.1038/s41586-024-07033-8. 38326617 PMC10881390

[36] R. Fittipaldi , R. Hartmann , M. T. Mercaldo , et al., “Unveiling Unconventional Magnetism at the Surface of ${\mathrm{Sr}}_{2}{\mathrm{RuO}}_{4}$ ,” Nature Communications 12, no. 1 (2021): 5792, 10.1038/s41467-021-26020-5. PMC8490454 34608149

[37] M. Kang , S. Kim , Y. Qian , et al., “Measurements of the Quantum Geometric Tensor in Solids,” Nature Physics (2024), 10.1038/s41567-024-02678-8.

[38] T. C. Hsu , J. B. Marston , and I. Affleck , “Two Observable Features of the Staggered‐Flux Phase at Nonzero Doping,” Physical Review B 43 (Feb 1991): 2866–2877, https://link.aps.org/doi/10.1103/PhysRevB.43.2866. 10.1103/physrevb.43.2866 9997586

[39] C. M. Varma , “Non‐Fermi‐Liquid States and Pairing Instability of a General Model of Copper Oxide Metals,” Physical Review B 55 (1997): 14 554–14 580.

[40] I. Affleck and J. B. Marston , “Large‐n Limit of the Heisenberg‐Hubbard Model: Implications for High‐$t_{c}$ Superconductors,” Physical Review B 37 (1988): 3774–3777. 10.1103/physrevb.37.3774 9944997

[41] P. Bourges , D. Bounoua , and Y. Sidis , “Loop Currents in Quantum Matter,” Comptes Rendus Physique 22, no. S5 (2021): 7–31.

[42] H. Li , S. Cheng , B. R. Ortiz , et al., “Electronic Nematicity Without Charge Density Waves in Titanium‐Based Kagome Metal,” Nature Physics 19, no. 11 (Nov 2023): 1591–1598, 10.1038/s41567-023-02176-3.

[43] C. Bigi , M. Dürrnagel , L. Klebl , et al., “Pomeranchuk Instability From Electronic Correlations in ${\mathrm{CsTi}}_{3}{\mathrm{Bi}}_{5}$ Kagome Metal,” Nature Communications 17, no. 1 (Dec 2025): 325, 10.1038/s41467-025-67037-4. PMC12789546 41436439

[44] K. Koepernik and H. Eschrig , “Full‐Potential Nonorthogonal Local‐Orbital Minimum‐Basis Band‐Structure Scheme,” Physical Review B 59 (Jan 1999): 1743–1757, https://link.aps.org/doi/10.1103/PhysRevB.59.1743.

[45] J. P. Perdew and Y. Wang , “Accurate and Simple Analytic Representation of the Electron‐Gas Correlation Energy,” Physical Review B 45 (Jun 1992): 13 244–13 249, https://link.aps.org/doi/10.1103/PhysRevB.45.13244. 10.1103/physrevb.45.13244 10001404

[46] G. H. Wannier , “The Structure of Electronic Excitation Levels in Insulating Crystals,” Physical Review 52 (Aug 1937): 191–197, https://link.aps.org/doi/10.1103/PhysRev.52.191.

[47] G. H. Wannier , “Dynamics of Band Electrons in Electric and Magnetic Fields,” Reviews of Modern Physics 34 (Oct 1962): 645–655, https://link.aps.org/doi/10.1103/RevModPhys.34.645.

[48] K. Koepernik and H. Eschrig , “Full‐Potential Nonorthogonal Local‐Orbital Minimum‐Basis Band‐Structure Scheme,” Physical Review B 59 (Jan 1999): 1743–1757, https://link.aps.org/doi/10.1103/PhysRevB.59.1743.

[49] I. Opahle , K. Koepernik , and H. Eschrig , “Full‐Potential Band‐Structure Calculation of Iron Pyrite,” Physical Review B 60 (Nov 1999): 14 035–14 041, https://link.aps.org/doi/10.1103/PhysRevB.60.14035.

[50] N. Marzari , A. A. Mostofi , J. R. Yates , I. Souza , and D. Vanderbilt , “Maximally Localized Wannier Functions: Theory and Applications,” Reviews of Modern Physics 84 (Oct 2012): 1419–1475, https://link.aps.org/doi/10.1103/RevModPhys.84.1419.

[51] G. Pizzi , V. Vitale , R. Arita , et al., “Wannier90 as a Community Code: New Features and Applications,” Journal of Physics: Condensed Matter 32, no. 16 (Jan 2020): 165902, 10.1088/1361-648X/ab51ff. 31658458

