# Supplementary material for: Anomalous Spin‐Optical Helical Effect in Ti‐Based Kagome Metal
Source: Adv Mater. 2026 Mar 18;38(21):e22533. doi: 10.1002/adma.202522533 (PMC13073107; doi:10.1002/adma.202522533)
Supplement: Supplementary file 1 — Supporting File: adma72799‐sup‐0001‐SuppMat.pdf. [file ADMA-38-e22533-s001.pdf]

Supplementary Information:Anomalous spin-optical helical effect in Ti-based kagome metal

Federico Mazzola**,[1,*] Wojciech Brzezicki**,[2,3,†] Chiara Bigi,[4] Armando Consiglio,[5] Luciano Jacopo D'Onofrio,[6] Maria Teresa Mercaldo,[7] Adam Kłosiński,[8] François Bertran,[4] Patrick Le Fèvre,[9] Oliver J. Clark,[10] Mark T. Edmonds,[10] Manuel Tuniz,[11] Alessandro De Vita,[12] Vincent Polewczyk,[13] Jeppe B. Jacobsen,[14] Henrik Jacobsen,[15] Jill A. Miwa,[16] Justin W. Wells,[17] Anupam Jana,[18] Ivana Vobornik,[18] Jun Fujii,[18] Niccolo Mignani,[19] Narges Samani Tarakameh,[19] Alberto Crepaldi,[19] Giorgio Sangiovanni,[20] Anshu Kataria,[21] Tommaso Morresi,[22] Samuele Sanna,[23] Pietro Bonfá,[21] Brenden R. Ortiz,[24] Ganesh Pokharel,[25,26] Stephen D. Wilson,[25] Domenico Di Sante,[23] Carmine Ortix,[7] and Mario Cuoco[6,‡]

[1]*CNR-SPIN, c/o Complesso di Monte S. Angelo, IT-80126 Napoli, Italy*

[2]*Institute of Theoretical Physics, Jagiellonian University, ulic, S. Łojasiewicza 11, PL-30348 Kraków, Poland*

[3]*International Research Centre MagTop, Institute of Physics, Polish Academy of Sciences, Aleja Lotników 32/46, PL-02668 Warsaw, Poland*

[4]*Synchrotron SOLEIL, L'Orme des Merisiers, Départementale 128, F-91190 Saint-Aubin, France*

[5]*Istituto Officina dei Materiali, Consiglio Nazionale delle Ricerche, Trieste I-34149, Italy*

[6]*CNR-SPIN, c/o Universitá di Salerno, IT-84084 Fisciano (SA), Italy*

[7]*Dipartimento di Fisica "E. R. Caianiello", Università di Salerno, IT-84084 Fisciano (SA), Italy*

[8]*Institute of Theoretical Physics, Faculty of Physics, University of Warsaw, Pasteura 5, PL-02093 Warsaw, Poland*

[9]*Univ Rennes, IPR Institut de Physique de Rennes, UMR 6251, F-35000 Rennes, France*

[10]*School of Physics and Astronomy, Monash University, Clayton, Victoria 3800, Australia*

[11]*Dipartimento di Fisica, Universita degli studi di Trieste, 34127, Trieste, Italy*

[12]*Fritz Haber Institut der Max Planck Gesellshaft, Faradayweg 4–6, 14195 Berlin, Germany*

[13]*Université Paris-Saclay, UVSQ, CNRS, GEMaC, 78000, Versailles, France*

[14]*Nanoscience Center, Niels Bohr Institute, University of Copenhagen, 2100 Copenhagen, Denmark*

[15]*European Spallation Source ERIC - Data Management and Software Center, 2800 Kgs. Lyngby, Denmark*

[16]*Department of Physics and Astronomy, Interdisciplinary Nanoscience Center, Aarhus University, 8000 Aarhus C, Denmark*

[17]*Department of Physics and Centre for Materials Science and Nanotechnology, University of Oslo (UiO), 0318 Oslo, Norway.*

[18]*CNR-IOM Istituto Officina dei Materiali, I-34139 Trieste, Italy*

[19]*Dipartimento di Fisica, Politecnico di Milano,*

Piazza Leonardo Da Vinci 32, Milano 20133, Italy
[20]*Institute for Theoretical Physics and Astrophysics, University of Würzburg, D-97074 Würzburg, Germany*
[21]*Dipartimento di Scienze Matematiche, Fisiche e Informatiche, Universitá di Parma, I-43124 Parma, Italy*
[22]*European Centre for Theoretical Studies in Nuclear Physics and Related Areas (ECT*), Fondazione Bruno Kessler, Trento, Italy*
[23]*Department of Physics and Astronomy, University of Bologna, 40127 Bologna, Italy*
[24]*Materials Science and Technology Division, Oak Ridge National Laboratory, Oak Ridge, TN 37831, USA*
[25]*Materials Department, University of California Santa Barbara, Santa Barbara, California 93106, USA*
[26]*Perry College of Mathematics, Computing, and Sciences, University of West Georgia, Carrollton, GA 30118, USA*

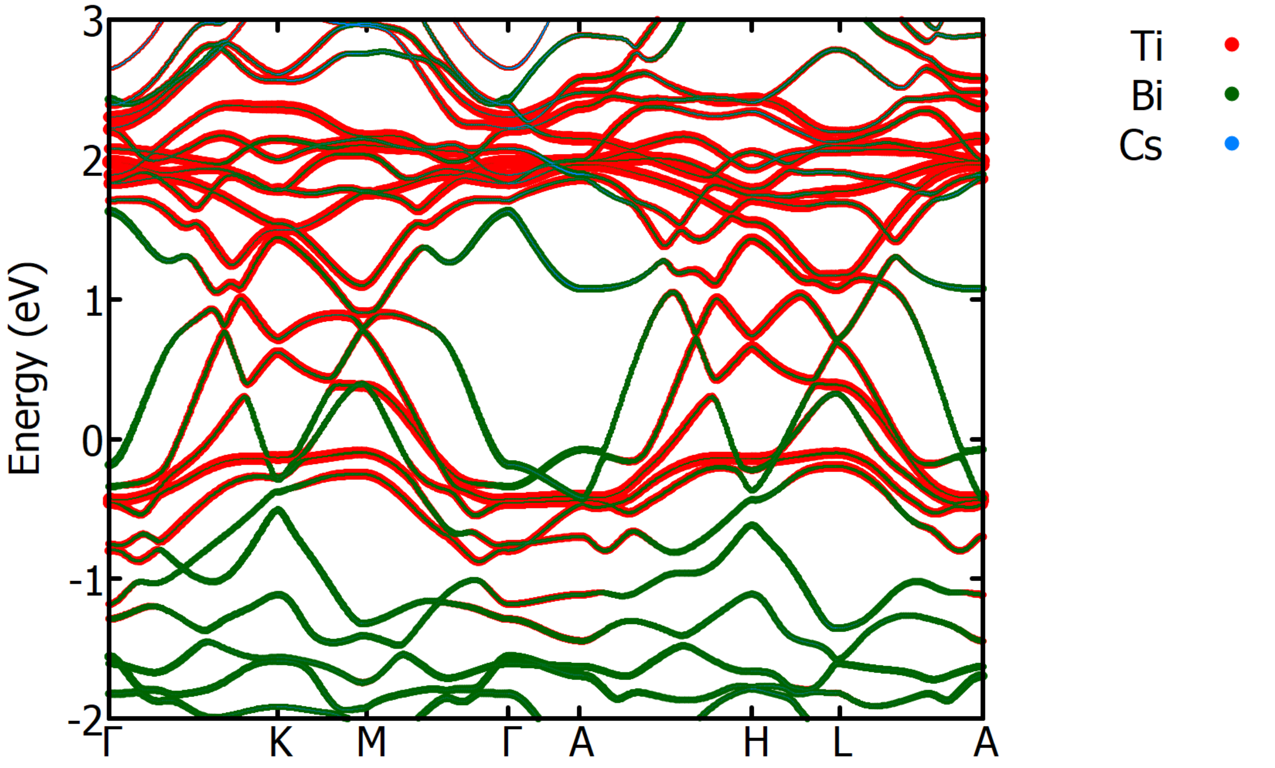

FIG. 1. Electronic structure from density functional theory, showing the projected spectral weight corresponding to Ti, Bi, and Cs atomic contributions.

I. SYMMETRY-ADAPTED WANNIER MODEL CONSTRUCTION

To derive an effective low-energy description, we constructed a Wannier-function-based model [1, 2] with the crystal symmetries explicitly enforced. The initial set of projection orbitals comprised Cs $6s$; Ti $3d_{z^2}$, $3d_{xz}$, $3d_{yz}$, $3d_{x^2-y^2}$, and $3d_{xy}$; and Bi $6p_z$, $6p_x$, $6p_y$, and $6s$ states.

The Wannier functions were generated using the projection scheme implemented in the FPLO code [3, 4]. In this approach, Kohn–Sham Bloch states within a chosen energy window are projected onto localized atomic-like orbitals. This procedure yields symmetry-adapted Wannier functions by construction, without requiring the iterative minimization of the spread functional employed in the maximally localized Wannier function (MLWF) approach as implemented, for example, in `wannier90` [5, 6].

Although the initial projection included Ti $3d$, Bi $6p/6s$, and Cs $6s$ orbitals, the electronic states in the vicinity of the Fermi level are predominantly of Ti $3d$ character (see Fig. 1). While Bi and Cs orbitals contribute with finite spectral weight, their role in shaping the kagome-derived bands of interest is comparatively minor. Consequently, the

* federico.mazzola@spin.cnr.it
† w.brzezicki@uj.edu.pl
‡ mario.cuoco@spin.cnr.it

effective tight-binding model is constructed by retaining only the Ti $3d$ Wannier functions.

II. 2D TIGHT-BINDING MODEL AND LOOP CURRENTS STATES

Based on the density functional theory calculations we construct a 2D tight-binding model that is based only on the d-orbitals of Ti. To this aim we employ the basis of cubic harmonics d_{xy}, d_{yz}, d_{zx}, $d_{x^2-y^2}$ and $d_{3z^2-r^2}$ and thus we need the $L=2$ angular momentum matrices to describe the corresponding orbital dependent electronic processes.

$$\hat{L}_x = \begin{pmatrix} 0 & 0 & -i & 0 & 0 \\ 0 & 0 & 0 & -i & -i\sqrt{3} \\ i & 0 & 0 & 0 & 0 \\ 0 & i & 0 & 0 & 0 \\ 0 & i\sqrt{3} & 0 & 0 & 0 \end{pmatrix}, \hat{L}_y = \begin{pmatrix} 0 & i & 0 & 0 & 0 \\ -i & 0 & 0 & 0 & 0 \\ 0 & 0 & 0 & -i & i\sqrt{3} \\ 0 & 0 & i & 0 & 0 \\ 0 & 0 & -i\sqrt{3} & 0 & 0 \end{pmatrix}, \hat{L}_z = \begin{pmatrix} 0 & 0 & 0 & 2i & 0 \\ 0 & 0 & i & 0 & 0 \\ 0 & -i & 0 & 0 & 0 \\ -2i & 0 & 0 & 0 & 0 \\ 0 & 0 & 0 & 0 & 0 \end{pmatrix} \tag{1}$$

Moreover, in order to capture the tri-sublattice structure of the unit cell of the kagome lattice we employ a $T=1$ sublattice pseudospin moment whose components are expressed:

$$\hat{T}_x = \begin{pmatrix} 0 & 0 & 0 \\ 0 & 0 & -i \\ 0 & i & 0 \end{pmatrix}, \quad \hat{T}_y = \begin{pmatrix} 0 & 0 & i \\ 0 & 0 & 0 \\ -i & 0 & 0 \end{pmatrix}, \quad \hat{T}_z = \begin{pmatrix} 0 & -i & 0 \\ i & 0 & 0 \\ 0 & 0 & 0 \end{pmatrix}. \tag{2}$$

Now, we can define hopping elements between sites 1 and 2 of the unit cell as (see Fig. 2):

$$\begin{aligned} \hat{t}_{12} = & \frac{1}{6} dd\sigma \left(\hat{L}_x^2 + \hat{L}_z^2\right) + \frac{1}{12}\left(5dd\delta + 5dd\sigma - 8dd\pi\right)\hat{L}_y^2 \\ & + \frac{1}{24}\left(4dd\pi - 3dd\sigma - dd\delta\right)\left(\left\{\hat{L}_x^2, \hat{L}_y^2\right\} + \left\{\hat{L}_y^2, \hat{L}_z^2\right\}\right) \\ & + ia\hat{L}_z + ib\left\{\hat{L}_y^2, \hat{L}_z\right\} + ic\left\{\hat{L}_x^2, \hat{L}_z\right\} + d\hat{L}_z^2 + e\left\{\hat{L}_y^2, \hat{L}_z^2\right\} + f\left\{\hat{L}_z^2, \hat{L}_x^2\right\}. \end{aligned} \tag{3}$$

Here, $dd\pi$, $dd\sigma$ and $dd\delta$ Slater-Koster parameters are the only ones that are non-vanishing in the case of a fully symmetric simple kagome lattice. However due to the presence of other elements in the lattice (for instance the Bi in the center of the unit cell) also the parameters a, b, c, d, e, f are non-vanishing, though being small in amplitude. We also need to define the onsite crystal-field splitting elements for site 3:

$$\hat{h}_3 = q_x\hat{L}_x^2 + q_y\hat{L}_y^2 + q_z\hat{L}_z^2 + r_x\left\{\hat{L}_y^2, \hat{L}_z^2\right\} + r_y\left\{\hat{L}_x^2, \hat{L}_x^2\right\} + r_z\left\{\hat{L}_x^2, \hat{L}_y^2\right\}. \tag{4}$$

Similarly, to get hopping and crystal field terms between other sites in the unit cell it is useful to define a 120° orbital rotation matrix: $\hat{C}_3 = \exp\left[i2\pi/3\hat{L}_z\right]$ (it implicity includes the rotation of the site index within the unit cell). The operators $\hat{h}_1$ and $\hat{h}_2$ can be deduced from the expression of the operator h_3 through rotations of 120 degrees as generated by the rotation operator. According to the site labels in Fig. 1 of the Supplemental Information, we have that the operator $\hat{h}_1 = \hat{C}_3\hat{h}_3\hat{C}_3^\dagger$ while $\hat{h}_2 = \hat{C}_3^\dagger\hat{h}_3\hat{C}_3$. Similarly, we have that from the expression of the hopping at the bond 12 and and local terms at one of the site of the unit cell one can construct the Hamiltonian for the other

symmetry related bonds and sites:

$$\hat{t}_{31} = \hat{C}_3^\dagger \hat{t}_{12} \hat{C}_3, \quad \hat{t}_{23} = \hat{C}_3 \hat{t}_{12} \hat{C}_3^\dagger, \tag{5}$$

Then, we are ready to define a tight-binding Hamiltonian, we have:

$$\begin{aligned}\hat{H}_{\vec{k}} = &- \left[1 + e^{-i\frac{1}{2}\left(k_x + \sqrt{3}k_y\right)}\right] \hat{t}_{31} \otimes \left(\hat{T}_x \hat{T}_z\right) - \left[1 + e^{i\frac{1}{2}\left(k_x + \sqrt{3}k_y\right)}\right] \hat{t}_{13} \otimes \left(\hat{T}_z \hat{T}_x\right) \\ &- \left[1 + e^{-ik_x}\right] \hat{t}_{21} \otimes \left(\hat{T}_x \hat{T}_y\right) - \left[1 + e^{ik_x}\right] \hat{t}_{12} \otimes \left(\hat{T}_y \hat{T}_x\right) \\ &- \left[1 + e^{-i\frac{1}{2}\left(-k_x + \sqrt{3}k_y\right)}\right] \hat{t}_{32} \otimes \left(\hat{T}_y \hat{T}_z\right) - \left[1 + e^{i\frac{1}{2}\left(-k_x + \sqrt{3}k_y\right)}\right] \hat{t}_{23} \otimes \left(\hat{T}_z \hat{T}_y\right) \\ &+ \hat{h}_1 \otimes \left(1 - \hat{T}_x^2\right) + \hat{h}_2 \otimes \left(1 - \hat{T}_y^2\right) + \hat{h}_3 \otimes \left(1 - \hat{T}_z^2\right). \end{aligned} \tag{6}$$

The atomic spin-orbit coupling at the Ti site is expressed as $\lambda_{so} \hat{L} \cdot \hat{s}$. For convenience, we have discarded λ_{so} because it is relatively small compared to the other energy scales involved in the problem and does not significantly influence the results of the analysis. The values of the hopping parameters extracted from the Wannier projection analysis are the following (in units of eV):

$$\begin{aligned} dd\pi = 0.3433,\ dd\sigma = -0.9807,\ dd\delta = 0.0267 \\ a = -0.0894,\ b = -0.0013,\ c = 0.0280 \\ d = 0.0759,\ e = -0.0232,\ f = 0.0081 \\ q_x = 0.0720,\ q_y = 0.3035,\ q_z = 0.0246 \\ r_x = -0.0124,\ r_y = 0.0653,\ r_z = -0.0278. \end{aligned} \tag{7}$$

To describe the loop current phases, it is advantageous to use current operators that are directly formulated in terms of the sublattice-spin-orbital operators. The loop current phase with isotropic charge circulation within the unit cell can be expressed by the following symmetry breaking term

$$\hat{J}_{\mathrm{c}} = \left(\hat{T}_x + \hat{T}_y + \hat{T}_z\right), \tag{8}$$

since $\hat{T}_x$,$\hat{T}_y$, and $\hat{T}_z$ correspond to the current operators on the three bonds within the unit cell.

Similarly, one can introduce loop current states that possess orbital or spin-orbital quadrupoles. The orbital quadrupoles within the unit cell can be represented by means of the mirror symmetric fields $\hat{A} = (\hat{A}_x, \hat{A}_y, \hat{A}_z)$, with $\hat{A}_x = \hat{L}_x \hat{L}_x$, and mirror broken orbital operators $\hat{\xi} = (\hat{\xi}_x, \hat{\xi}_y, \hat{\xi}_z)$, where $\hat{\xi}_x = \hat{L}_y \hat{L}_z + \hat{L}_z \hat{L}_y$, with other components derived through index permutation. For the purposes of our work we are interested in the mirror broken loop current state. This configuration can be generally expressed by having symmetry breaking current terms in the Hamiltonian that are superposition of the $\hat{\xi}$ components. Thus, it is expressed as $\hat{J}_o = (\hat{T}_x + \hat{T}_y + \hat{T}_z)(\chi_x \hat{\xi}_x + \chi_y \hat{\xi}_y + \chi_z \hat{\xi}_z)$ with the coefficients χ_i ($i = x, y, z$) defining the pattern of the orbital quadrupole that breaks rotation and mirror symmetries. The same approach can be followed when constructing the spin-orbital quadrupoles. In this case one can have different configurations depending on the orientation of the spin and orbital moments. The loop current configuration is consistently established by the charge circulation within the unit cell and is represented through the

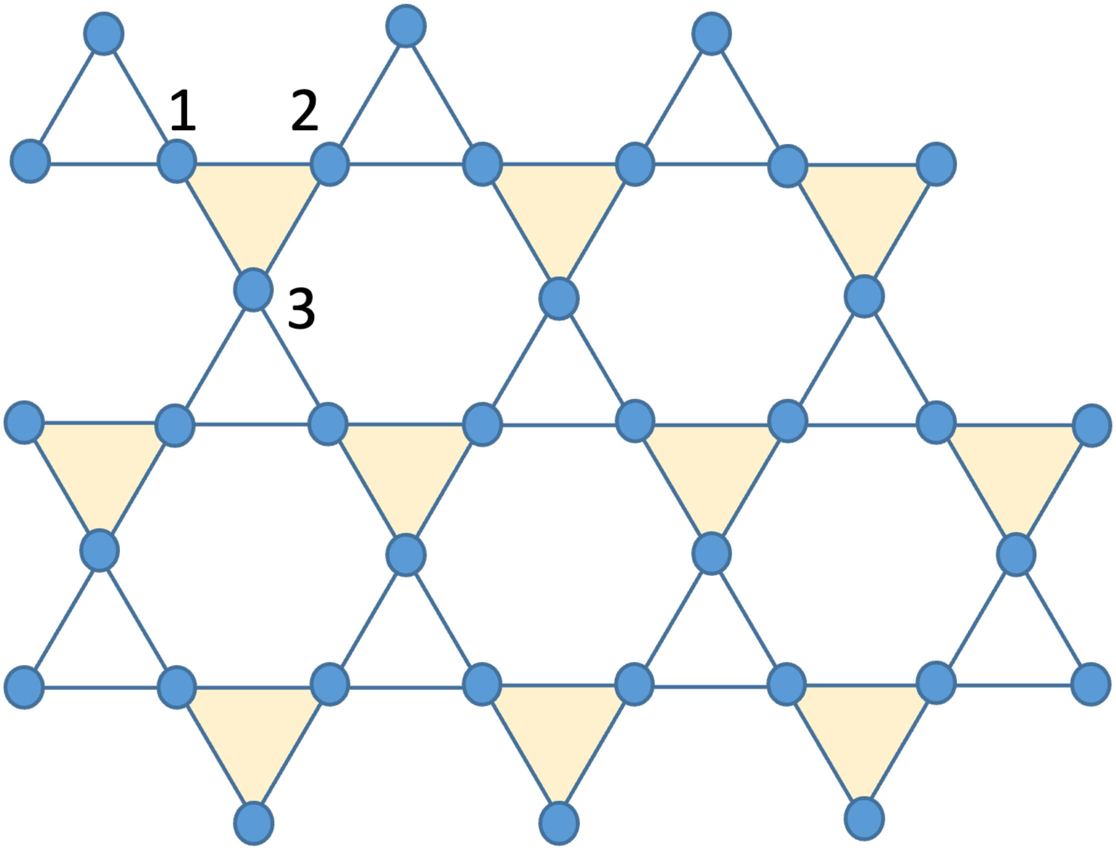

FIG. 2. Kagome lattice structure with the labels $\{1, 2, 3\}$ indicating the positions of the Ti atoms in the unit cell.

combination of the sublattice operators $\left(\hat{T}_x + \hat{T}_y + \hat{T}_z\right)$. Instead, for the spin-orbital quadrupole, configurations can be constructed that are either mirror and rotationally symmetric or exhibit broken mirror and rotational symmetry in the spin-orbital space. The symmetric loop current phase is associated to the current operators of the type $\hat{A}_{so} = (\hat{T}_x+\hat{T}_y+\hat{T}_z)(a_x\hat{L}_x\hat{s}_x+a_y\hat{L}_y\hat{s}_y+a_z\hat{L}_z\hat{s}_z)$. These phases do not lead to anomalies in the spin-dichroic and handedness-resolved spin-responses for the photoemission intensity. The spin-orbital quadrupole loop current phases that we have considered in the manuscript are marked by cross-spin-orbital correlations associated with the components of the term $\hat{\mathbf{L}} \times \hat{\mathbf{s}}$. A representative configuration of this type of spin-orbital quadrupole loop current phase can then be expressed as $\hat{J}_{so} = g_{so}(\hat{T}_x + \hat{T}_y + \hat{T}_z)[\mathbf{n} \cdot (\hat{\mathbf{L}} \times \hat{\mathbf{s}})]$ with $\mathbf{n} = (n_x, n_y, n_z)$ setting the director for the spin-orbital quadrupole distribution.

III. MEAN FIELD DECOUPLING AND TEMPERATURE DEPENDENCE OF SPIN-ORBITAL QUADRUPOLE LOOP CURRENT PHASE

It is useful to provide details about the construction of the quadrupolar order parameter. The electronic current phase can arise as a broken symmetry state with a nonvanishing expectation value of the current operator on the Ti-Ti bond within the unit cell due to the short-range Coulomb interaction. Indeed, one needs to introduce the spin-orbital

asymmetric current operator

$$\phi^{\alpha\beta}_{\sigma,\sigma'}(l,m) = i\left(c^{\dagger}_{\alpha,\sigma}(l)c_{\beta,\sigma'}(m) - c^{\dagger}_{\beta,\sigma'}(m)c_{\alpha,\sigma}(l)\right) \tag{9}$$

for the $l-m$ bond between two Ti atoms with position identified by the coordinates R_l and R_m. Here, $c_{\alpha,\sigma}(l)(c^{\dagger}_{\alpha,\sigma}(l))$ are the annihilation (creation) operators associated with an electronic state with α orbital (α labeling (d_{xy}, d_{yz}, d_{zx}, $d_{x^2-y^2}$, $d_{3z^2-r^2}$) and spin σ at the atomic site R_l. Then, the spin and orbital dependent terms that build up the density-density inter-site Coulomb interaction V_{lm} for a generic $l-m$ bond can be written in the following form

$$V_{lm}n_{\alpha,\sigma}(l)n_{\beta,\sigma'}(m) = -\frac{1}{2}V_{lm}(\phi^{\alpha\beta}_{\sigma,\sigma'}(l,m))^{\dagger}\phi^{\alpha\beta}_{\sigma,\sigma'}(l,m) + \frac{1}{2}V_{lm}\left(n_{\alpha,\sigma}(l) + n_{\beta,\sigma'}(m)\right) \tag{10}$$

where the orbital and spin resolved density operators, $n_{\alpha,\sigma}(l)$, are defined as $n_{\alpha,\sigma}(l) = c^{\dagger}_{\alpha,\sigma}(l)c_{\alpha,\sigma}(l)$. Hence, by decoupling the quartic term, one can introduce an order parameter associated with the expectation value of the operator $\phi^{\alpha\beta}_{\sigma,\sigma'}(l,m)$ and express the interaction as

$$V_{lm}n_{\alpha,\sigma}(l)n_{\beta,\sigma'}(m) \sim -\frac{1}{2}V_{lm}\left[\langle\phi^{\alpha\beta}_{\sigma,\sigma'}(l,m)\rangle(\phi^{\alpha\beta}_{\sigma,\sigma'}(l,m))^{\dagger} + h.c. - |\langle\phi^{\alpha\beta}_{\sigma,\sigma'}(l,m)\rangle|^2\right] \tag{11}$$

where the average value indicates the summation over all the electronic states weighted by the Fermi distribution function. Taking into account the spin-orbital order parameters on the $l-m$ bond, by suitable superposition of the ϕ operators one can construct a bond current order parameter that is given by the expectation value of the following orbital and spin-orbital quadrupole current operators:

$$\begin{aligned} J^{o}_{i,j}(l,m) &= i\left(\vec{c}^{\,\dagger}(l)\hat{L}_i\hat{L}_j\vec{c}(m) - h.c.\right) \\ J^{so}_{i,j}(l,m) &= i\left(\vec{c}^{\,\dagger}(l)\hat{L}_i\hat{S}_j\vec{c}(m) - h.c.\right), \end{aligned} \tag{12}$$

with the vector $\vec{c}^{\,\dagger}(l)$ defined as

$$\vec{c}^{\,\dagger}(l) = \left[c^{\dagger}_{xy,\uparrow}(l), c^{\dagger}_{yz,\uparrow}(l), c^{\dagger}_{zx,\uparrow}(l), c^{\dagger}_{3z^2-r^2,\uparrow}(l), c^{\dagger}_{x^2-y^2,\uparrow}(l), c^{\dagger}_{xy,\downarrow}(l), c^{\dagger}_{yz,\downarrow}(l), c^{\dagger}_{zx,\downarrow}(l), c^{\dagger}_{3z^2-r^2,\downarrow}(l), c^{\dagger}_{x^2-y^2,\downarrow}(l)\right] \tag{13}$$

and $i, j \in \{x, y, z\}$. Then, by combining the various quadrupolar current contributions, one can construct the following term in the unit cell as a source of the spin-quadrupole loop current

$$H_{J_{so}} = -V\left[(\langle\hat{J}_{so}\rangle\hat{J}_{so} + h.c.) - |\langle\hat{J}_{so}\rangle|^2\right] \tag{14}$$

with $\hat{J}_{so} = (\hat{T}_x + \hat{T}_y + \hat{T}_z)[\mathbf{n}\cdot(\hat{\mathbf{L}}\times\hat{\mathbf{s}})]$ with $\mathbf{n} = (n_x, n_y, n_z)$ and we assumed that $V_{lm} = V$ for each Ti-Ti bond within the unit cell. In an analogous manner, solutions corresponding to different quadrupolar loop-current phases can be constructed as manifestations of pure orbital quadrupoles.

Taking into this approach we can evaluate the free energy in terms of the expectation value $\langle\hat{J}_{so}\rangle$ ($\langle\cdot\rangle$ indicates the thermal average) and obtain its minimum as a function of temperature. As expected thermal fluctuations destroy the

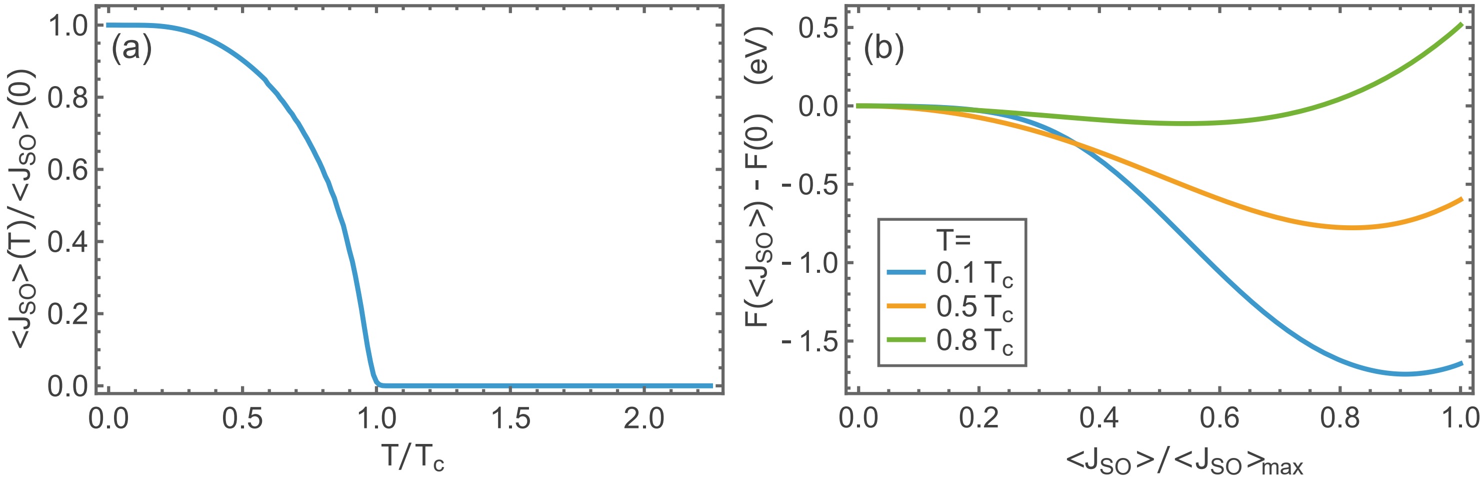

FIG. 3. Results of the mean-field calculation for the spin-orbital quadrupole loop current phase. (a) Order parameter (thermal average of the spin-orbital quadrupole current operator J_{SO}) versus scaled temperature $\frac{T}{T_c}$. (b) Free energy versus order parameter for representative temperatures. The order parameter is rescaled with respect to its maximum amplitude at zero temperature. The other parameters are $V = 0.01$ eV and the direction of the spin-orbital $\vec{g}$ vector in the spherical coordinates is $\theta = \pi/4$ and $\phi = \pi/4$.

quadrupole loop current phase. We have examined the phase transition associated with the spin–orbital quadrupole current phase and find that it is of second-order character. In Fig. 3 we have reported the temperature evolution of the order parameter associated with the spin-orbital quadrupole loop current operator in the unit cell. The behavior follows a standard second order phase transition with the minimum of the free energy that shifts to zero amplitude of the order parameter as the transition temperature is approached. Within a mean-field decoupling scheme, we simulate a representative value for a nearest-neighbor Coulomb interaction $V = 20\,\mathrm{meV}$ yields a transition temperature of the order of $200\,\mathrm{meV}$, indicating that this ordering is expected to occur at temperatures well above room temperature. Our results show that this type of ordering can emerge at temperatures far exceeding room temperature, even when the Coulomb interaction is relatively weak. This behavior arises because the energy gain of the spin–orbital quadrupole state is determined by the intrinsic energy scale of the orbital quadrupoles set by the crystalline electric field. In the present system, this includes contributions on the order of hundred of meV, as well as substantial orbital-dependent Ti–Ti hopping amplitudes. By contrast, in loop-current phases that do not involve spin or orbital quadrupoles linked to atomic orbital angular momentum, the transition temperature is typically controlled solely by the electronic hopping scale, t.

IV. LOOP CURRENTS SPIN-ORBITAL QUADRUPOLE DOMAINS

The nature of the proposed loop currents, due to the spin-orbital quadrupolar degrees of freedom, gives rise to a large variety of degenerate domains with opposite chirality, which hinder the cancellation of the observed anomaly.

A key aspect to consider when examining the domain structure is that the loop currents we propose—responsible

for the spin-resolved helical anomaly—possess a distinctive spin-orbital quadrupolar configuration. As detailed in the main text, the symmetry breaking of the quadrupolar current can be characterized by an n-vector, to specify the spin-orbital quadrupole pattern. Specifically, the spin-orbital current can be expressed as $\hat{J}_{\text{so}} = g_{\text{so}}(\hat{T}_x + \hat{T}_y + \hat{T}_z)[\mathbf{n}\cdot(\hat{\mathbf{L}}\times\hat{\mathbf{s}})]$, where $\mathbf{n} = (n_x, n_y, n_z)$ defines the spin-orbital quadrupole distribution, and g_{so} represents the coupling strength associated with the loop-current state. Taking into account the structure of this order parameter, one can find that, due to the trigonal symmetry of the unit cell, the manifold of equally energy states is made of 24 configurations. Indeed, assuming that the symmetry broken state is related to a quadrupole loop current represented by a given vector $\mathbf{n}$, there are 23 symmetry related configurations which are degenerate in energy. The symmetry operations that are relevant for the construction of the degenerate energy manifold are the vertical/horizontal mirrors, threefold rotation around the out-of-plane axis (see Fig. 4) and time-reversal symmetry. For convenience and clarity we indicate the vertical and horizontal mirrors of the kagome lattice as M_1, M_2, M_3, M_{z}, while the threefold rotation symmetry is given by C_3. The vector $\mathbf{n}$ transforms upon the application of the point group symmetry in the standard way, for instance, the solution $M_z.\mathbf{n} = \{n_x, n_y, -n_z\}$. Moreover, the time partner configuration with opposite circulation of the quadrupole loop current is obtained by reversing the sign of the coupling, as $T.\mathbf{n} = \{-n_x, -n_y, -n_z\}$, with T being the time reversal symmetry operator. Taking into account the relation among the three vertical mirrors and the threefold rotation, one can have 24 independent solutions that are degenerate in energy. They are given by the following twelve configurations $\{\Gamma_i\} = \{\text{n}, M_1.\text{n}, M_2\text{n}, M_3.\text{n}, M_z.\text{n}, C_3.\text{n}, C_3^{-1}.\text{n}, M_z C_3.\text{n}, M_z C_3^{-1}.\text{n}, M_z M_1.\text{n}, M_z M_2.\text{n}, M_z M_3.\text{n}\}$ and those ones that are the time reversal symmetric, i.e. $T\Gamma_i$, with the loop current flow circulating in the opposite direction as compared to that of $\mathbf{n}$.

Before discussing the energetics of configurations with different domains, there are few observations to be made about the various loop current states and the corresponding spin optical anomalies evaluated. Let us compare a configuration with a given $\tilde{\mathbf{n}}$ spin-orbital quadrupole distribution and the time reversal partner $T.\tilde{\mathbf{n}}$, having the same spin-orbital quadrupole but opposite sign of the loop current circulation. Then, one finds that the spin (s_z) and orbital (L_z) polarization for the states at the center of the Brilluoin zone are reversed in sign, i.e. $s_z(\tilde{\mathbf{n}}) = -s_z(T.\tilde{\mathbf{n}})$ and $L_z(\tilde{\mathbf{n}}) = -L_z(T.\tilde{\mathbf{n}})$. Furthermore, the amplitudes of the spin-resolved single-handedness polarizations—such as $(L^+\sigma_-, L^-\sigma_+)$- are interchanged when moving from $\tilde{\mathbf{n}}$ to its time-reversal counterpart $T.\tilde{\mathbf{n}}$. This relationship can be expressed as $L^+\sigma_-(\tilde{\mathbf{n}}) = L^-\sigma_+(T.\tilde{\mathbf{n}})$. A similar relation holds for configurations related by mirror symmetry transformations, affecting the spin-resolved single-handedness polarizations in an analogous manner, i.e. $L^+\sigma_-(\tilde{\mathbf{n}}) = L^-\sigma_+(M_\alpha.\tilde{\mathbf{n}})$ with $\alpha = 1, 2, 3, z$. Instead, the states which are obtained by C_3 rotation have equal single-handedness polarizations, namely $L^+\sigma_-(\tilde{\mathbf{n}}) = L^+\sigma_-(C_3.\tilde{\mathbf{n}})$ and the same for $L^-\sigma_+$. These symmetry relations imply that the difference of the amplitudes related to the left-polarized up (down) and the right-polarized down (up) signals transforms as a pseudoscalar physical observable. It then follows that the behavior of the spin-resolved single-handedness polarizations can be deduced when analyzing spin-orbital quadrupole states that incorporate mirror or rotational symmetries combined with time-reversal symmetry. Specifically, loop current states related by the product of an odd number of mirror reflections and the time-reversal operation—possibly including the threefold

rotation C_3 - such as $A = \{M_\alpha T, M_\alpha C_3 T\}$ with $\alpha = 1,2,3,z$ - share the same magnitude of the spin-resolved single-handedness polarization, i.e. $L^+\sigma_-(\tilde{\mathbf{n}}) = L^-\sigma_+(A.\tilde{\mathbf{n}})$. This implies that if the system breaks in domains with loop currents states that include combination of $\mathbf{n}$ and $A.\mathbf{n}$ spin-orbital quadrupole configurations there will be no cancellation in the amplitude asymmetric behavior manifested by the spin-resolved single-handedness polarizations. To quantitatively address this issue, we examined the energetics of a configuration consisting of two spatially separated domains (see Fig. 4C). In one domain, we assumed a specific n-vector state, while in the neighboring domain, we considered various spin-orbital quadrupole configurations corresponding to all possible degenerate states. Our analysis employed the tight-binding model derived from density functional theory (DFT), incorporating the spin-orbital quadrupole symmetry-breaking loop current term. Within one domain, we selected a representative loop current configuration characterized by the n-vector spherical angles: $\phi = 50°$ and $\theta = 25°$, where θ is measured relative to the z-axis and ϕ within the xy-plane. A variation of the n-vector does not alter the qualitative outcome. The system size was set to $L_1 = 20$ unit cells along the direction non-parallel to the domain wall and $L_2 = 20$ unit cells along the direction parallel to the domain wall (see Fig. 4c). We subsequently examined various electron filling configurations relative to the nominal value, $\langle n \rangle = n_0$, derived from DFT calculations. The results of this analysis are presented in Fig. 5. The phase diagram reveals a competitive interplay between different domain pattern types. Specifically, there are phases featuring loop current domains with identical spin-orbital quadrupole distributions but opposite chirality ($\tilde{\mathbf{n}}|T.\tilde{\mathbf{n}}$), as well as phases where both the chirality and the spin-orbital quadrupole structure differ ($\tilde{\mathbf{n}}|M_\alpha T.\tilde{\mathbf{n}}$), with $\alpha = 1,2,3,z$ —these latter are associated with the mirror-transformed director. Focusing first on the case where the electron density $\langle n \rangle = n_0$, we observe that the ground state configurations vary depending on the strength of the spin-orbital coupling g_{so}. At small values of g_{so}, the domains of the type ($\tilde{\mathbf{n}}|M_\alpha T.\tilde{\mathbf{n}}$) are close in energy to that of the type ($\tilde{\mathbf{n}}|T.\tilde{\mathbf{n}}$), leading to possible re-entrant transitions as a function of g_{so}. Hence, the domain characterized by loop currents with identical spin-orbital quadrupole but opposite chirality remains the ground state up to approximately $g_{\text{so}} = 0.2$eV. Beyond this value, the domain of the type ($\tilde{\mathbf{n}}|M_1 T.\tilde{\mathbf{n}}$) becomes energetically favored over others. The stability window of domains with phases where both the chirality and the spin-orbital quadrupole structure differ ($\tilde{\mathbf{n}}|M_\alpha T.\tilde{\mathbf{n}}$, $\alpha = 1,2,3,z$) is enhanced at small values of g_{so} when allowing slight variations in the electron filling, ranging from $\langle n \rangle = n_0$ to $\langle n \rangle = n_0 \pm 0.02$. This analysis suggests that when domains with opposite chirality form, they can feature inequivalent spin-orbital quadrupole distributions. In particular, domains involving loop currents with differing chirality and spin-orbital quadrupole structure—due to mirror-transformed directors - ($\tilde{\mathbf{n}}|M_\alpha T.\tilde{\mathbf{n}}$), with $\alpha = 1,2,3,z$ - cannot lead to a cancellation in the asymmetric amplitude of the spin-resolved single-handedness polarizations.

These findings suggest that the competition between loop current phases with opposite chirality and varying spin-orbital quadrupole configurations leads to domain structures displaying anomalies in spin-resolved single-handedness polarizations, which cannot be fully canceled out.

Hence, analyzing the various possible configurations of loop currents reveals that domain formation is primarily driven by interface energetics, which depend on the nature of the spin-orbital quadrupoles. Specifically, the coupling

at interfaces between states that are time-reversal and mirror partners results in differing energy stiffnesses, thereby favoring the emergence of a limited set of domains.

Furthermore, it is important to note that because spin-orbital quadrupole loop current states produce weak magnetic dipolar field, the contribution of long-range magnetic dipolar interactions to the energy is negligible. Since the typical size of the domains will be set by the competition between long-range dipolar forces – these try to minimize the domain size – and the short-range interface energy that instead tries to maximize the domain size, one can expect that only a few large domains, potentially spanning several microns will be formed. Given the large degeneracy of possible loop current states, the nucleation of a huge number of domains averaging to zero is unlikely. Therefore, in samples with large domains, the observed signals are mainly influenced by the anisotropy of the spin-orbital quadrupole orientation, which can consistently explain the persistent observation of a finite, unidirectional handedness and spin-resolved signals.

Let us further elaborate about the physical reason for having an interface which splits the energy of domains pairs. The studied loop currents exhibit a planar nature within the kagome lattice, resulting in domains with interfaces forming along the trigonal symmetry direction. When two neighbouring domains possess different spin-orbital quadrupole loop current patterns, the interface breaks certain symmetries—for instance, the vertical mirror symmetry in the configurations examined. This symmetry breaking is rooted into the internal spin-orbital quadrupole structure and the variations in loop current arrangements between the domains. Consequently, such symmetry breaking can produce interface effects, including the suppression or enhancement of the loop current spin-orbital quadrupole amplitude and the emergence of interface states that differ from those found in single-domain regions. However, when these states are coupled across the interface—particularly those related by vertical mirror reflection and time-reversal symmetry—their coupling can lead to a coexisting region that can approximately restore the broken symmetry. Indeed, when considering patterns with loop current having spin-orbital quadrupoles of the type, for instance, A and MA with M being the broken vertical mirror involved across the interface, the combination of these states can form a configuration that is mirror symmetric as $A + MA$. This coupling can produce states nearby the interface that are more symmetric under mirror reflection, effectively locking the proximitized loop current states and leading to an energy gain which is different as compared to the other domains configurations. This is consistent with what can be found by performing a full microscopic calculation based on the tight-binding model derived from the DFT analysis. As a result, the symmetry-breaking effects typically introduced by the interface are reduced and the amplitude of the spin-orbital quadrupole can be larger. Such phenomenon is rooted in the crystal's symmetry and the structure of the proposed spin-orbital quadrupole loop currents. Through this coupling at the interface, nucleation of neighbouring domains can develop a preferred pattern that filters specific configurations within the manifold of allowed spin-orbital loop current phases. This type of mechanism can thus account for having a non-random subset of domains that is consistently selected across multiple samples, cleaves, and measurement locations. A purely random distribution of time-reversal domains cannot satisfy this type of mechanism at the interface within the planar kagome lattice. Consequently, domains with loop currents that are time-reversal and mirror partners display the same spin-resolved

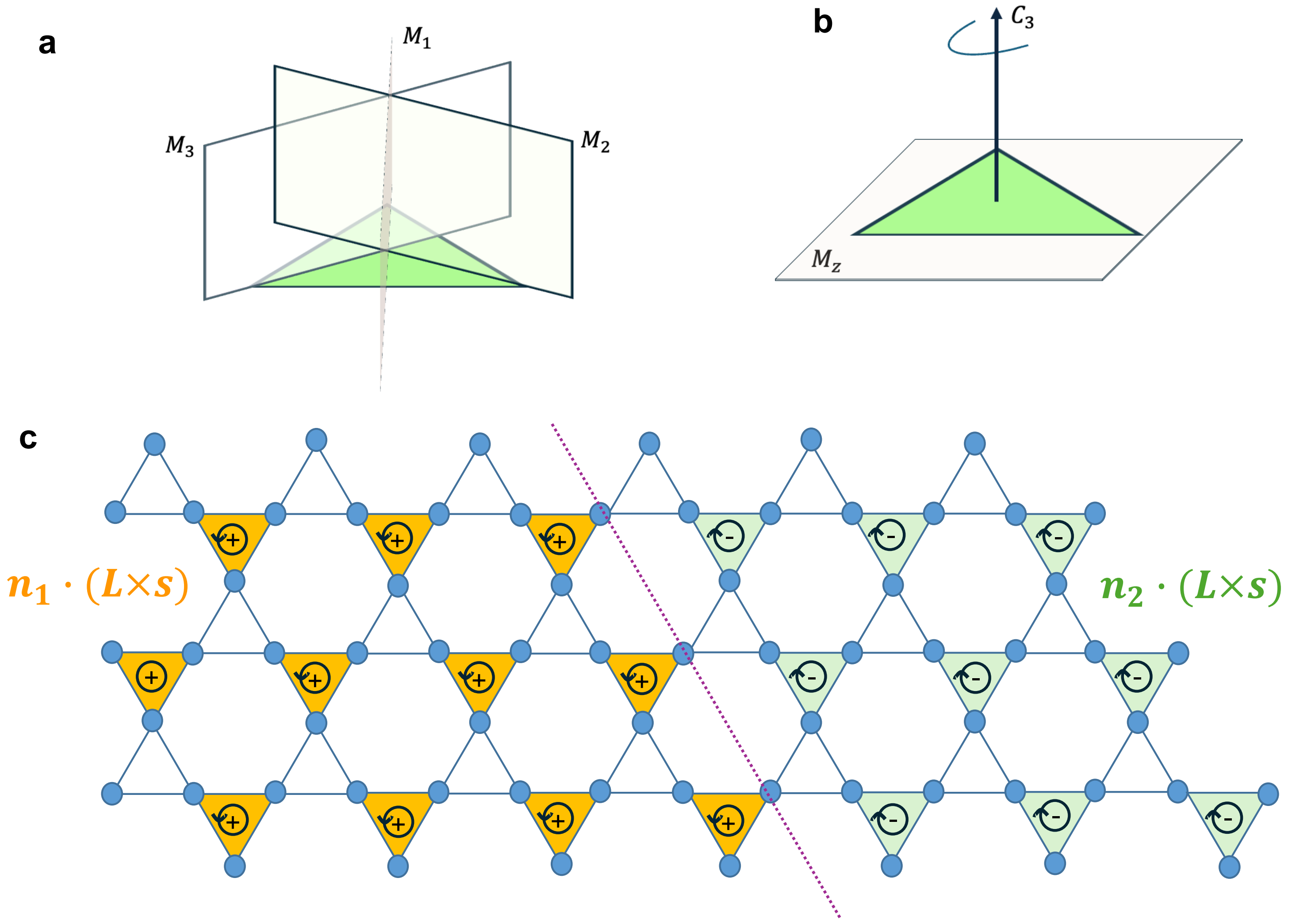

FIG. 4. Sketch of the mirrors and rotational symmetry transformations (**a**,**b**) for the trigonal configuration of the unit cell that are relevant for the construction of the manifold of energy degenerate loop current with distinct spin-orbital quadrupoles. **c** Representative structure of two domains with opposite chirality of the circulating loop current and spin-orbital quadrupoles identified by the vectors $\mathbf{n}_1$ and $\mathbf{n}_2$.

photoemission amplitude with a single handedness, thus consistently account for the experimental observations. Additionally, we would like to discuss about the anisotropy of the spin-orbital quandrupole loop current. It is important to note that the choice of the n-vector is related to the system's anisotropy, which is connected to the spin-orbital quadrupoles embedded in the electronic states at the Fermi level, crystal field effects, and spin-orbit coupling, similarly to the magnetic anisotropy in a ferromagnet. In our previous reply, we highlighted how bismuth (Bi) influences the spin-orbital Ti–Ti hopping terms, leading to cross correlations between spin and orbital angular momentum. The anisotropic nature of the resulting loop current phases is thus determined by the crystalline environment, leading to a consistent behavior of the spin-resolved response with a single handedness across multiple samples, cleaves, and measurement locations.

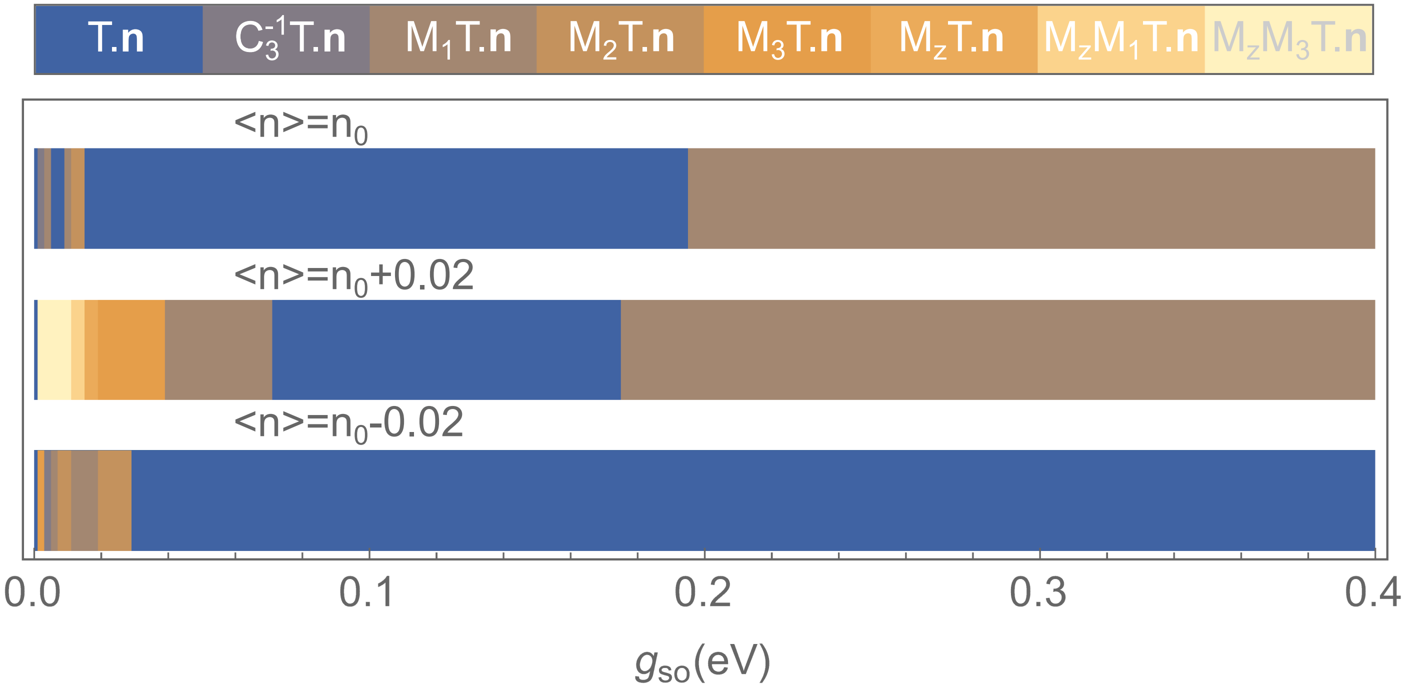

FIG. 5. Ground state phase diagram as a function of the coupling strength $g_{\rm so}$ associated with different types of spin-orbital quadrupole loop current configurations with opposite chirality with respect to a given n-director and involving symmetry related states through mirror M and threefold rotations C_3. The system includes two domains - as shown in Fig. 2c. One region has a configuration which is given by a representative n-vector with spherical angles: $\phi = 50°$ and $\theta = 25°$, where θ is measured relative to the z-axis and ϕ within the xy-plane. The other region features a spin-orbital quadrupole characterized by a configuration with opposite chirality, as given by the time-reversal operation T, combined with mirror M_α, for $\alpha = 1, 2, 3, z$, and with the rotational symmetry transformation C_3. The configurations corresponding to each domain pattern are indicated in the top row using different colors. The other rows display the evolution in terms of $g_{\rm so}$ of the lowest-energy states for various electron fillings $\langle n \rangle$ in proximity to the nominal filling as given by the DFT calculation.

V. ROLE OF BI IN MEDIATING TI-TI HYBRIDIZATION PROCESSES

We report the strategy and the details of the derivation of the effective spin-orbit terms which are induced by the Ti-Bi electron hybridization and the atomic spin-orbit coupling at the Bi site. The basic idea is that electrons in a specific orbital at the Ti site can hop to neighboring Ti atoms through hybridization with the p orbitals at the Bi site within the same unit cell. However, because of the significant Bi spin-orbit coupling, this hopping process generally becomes spin- and orbital- dependent. When the electronic states of Bi are projected out, considering second-order effects of Ti-Bi hopping, an effective local spin-orbit coupling emerges at the Ti site, along with spin-orbital-dependent Ti–Ti hoppings. We will show that at the lowest order in the Ti orbital angular momentum, due to the directions of the Ti–Bi bonds, we obtain an effective spin-orbit terms that include both collinear $s_i L_i$ components and cross terms $s_i L_k$, with $i \neq k$.

We consider the spin and orbital configurations that have been employed to model the electronic structure of the

$CsTi_3Bi_5$ system. Titanium (Ti) atoms are located at the vertices of the triangular plaquette that identifies the unit cell of the kagome lattice, and are labeled 1, 2, and 3, respectively. Additionally, a bismuth (Bi) atom with sizable spin-orbit coupling is positioned at the center of the plaquette. Our aim is to derive the effective Ti-Ti hopping processes and the local Ti terms that are due to the Ti-Bi-Ti electronic hybridization.

To this aim, it is useful to introduce a reference system $Oxyz$ so that the coordinates of the titanium atoms are the following ones:

$$\begin{cases} \mathbf{R}_1 = \{0, 1, 0\} \\ \mathbf{R}_2 = \{0, 0, 0\} \\ \mathbf{R}_3 = \left\{-\frac{\sqrt{3}}{2}, \frac{1}{2}, 0\right\} \end{cases} . \tag{15}$$

Thus, we can define the direction cosines of the vectors connecting two titanium atoms within the cell:

$$\begin{cases} \boldsymbol{b}_{12} = [0, -1, 0] \\ \boldsymbol{b}_{23} = \left[-\frac{\sqrt{3}}{2}, \frac{1}{2}, 0\right] \\ \boldsymbol{b}_{31} = \left[\frac{\sqrt{3}}{2}, \frac{1}{2}, 0\right] \end{cases} . \tag{16}$$

The coordinates of the bismuth atom are given by $\mathbf{R}_{Bi} = \left\{-\sqrt{3}/6, 1/2, z_{Bi}\right\}$ and we define the direction cosines of the vectors connecting each Ti atom to the Bi atom as follows:

$$\begin{cases} \boldsymbol{b}_{1Bi} = \left[-\frac{1}{2}\sin\theta, -\frac{\sqrt{3}}{2}\sin\theta, \cos\theta\right] \\ \boldsymbol{b}_{2Bi} = \left[-\frac{1}{2}\sin\theta, \frac{\sqrt{3}}{2}\sin\theta, \cos\theta\right] \\ \boldsymbol{b}_{3Bi} = [\sin\theta, 0, \cos\theta] \end{cases} . \tag{17}$$

Here, θ is the polar angle is the polar angle of the vector pointing from the origin to the bismuth atom.

To construct the effective model for Ti-Ti hybridization due to Ti-Bi-Ti hybridization, we employ d orbitals of the Ti atoms and p-ones of the bismuth atom. Thus, when considering the hopping term between two Ti atoms, we focus on the following Slater-Koster coefficients:

$$
\begin{cases}
t_{\mathrm{xy,\,xy}}[l,m,n] = 3l^2m^2\mathrm{dd}\sigma + (l^2+m^2-4l^2m^2)\mathrm{dd}\pi + (n^2+l^2m^2)\mathrm{dd}\delta,\\
t_{\mathrm{xy,\,yz}}[l,m,n] = 3lm^2n\mathrm{dd}\sigma + ln(1-4m^2)\mathrm{dd}\pi + ln(m^2-1)\mathrm{dd}\delta,\\
t_{\mathrm{xy,\,zx}}[l,m,n] = 3l^2mn\mathrm{dd}\sigma + mn(1-4l^2)\mathrm{dd}\pi + mn(l^2-1)\mathrm{dd}\delta,\\
t_{\mathrm{yz,\,yz}}[l,m,n] = 3m^2n^2\mathrm{dd}\sigma + (m^2+n^2-4m^2n^2)\mathrm{dd}\pi + (l^2+m^2n^2)\mathrm{dd}\delta,\\
t_{\mathrm{yz,\,zx}}[l,m,n] = 3mn^2l\mathrm{dd}\sigma + ml(1-4n^2)\mathrm{dd}\pi + ml(n^2-1)\mathrm{dd}\delta,\\
t_{\mathrm{yz,\,xy}}[l,m,n] = 3m^2nl\mathrm{dd}\sigma + nl(1-4m^2)\mathrm{dd}\pi + nl(m^2-1)\mathrm{dd}\delta,\\
t_{\mathrm{zx,\,zx}}[l,m,n] = 3n^2l^2\mathrm{dd}\sigma + (n^2+l^2-4n^2l^2)\mathrm{dd}\pi + (m^2+n^2l^2)\mathrm{dd}\delta,\\
t_{\mathrm{zx,\,xy}}[l,m,n] = 3nl^2m\mathrm{dd}\sigma + nm(1-4l^2)\mathrm{dd}\pi + nm(l^2-1)\mathrm{dd}\delta,\\
t_{\mathrm{zx,\,yz}}[l,m,n] = 3n^2lm\mathrm{dd}\sigma + lm(1-4n^2)\mathrm{dd}\pi + lm(n^2-1)\mathrm{dd}\delta,\\
t_{\mathrm{xy,\,x}^2-y^2}[l,m,n] = \frac{3}{2}lm(l^2-m^2)\mathrm{dd}\sigma + 2lm(m^2-l^2)\mathrm{dd}\pi + \frac{1}{2}lm(l^2-m^2)\mathrm{dd}\delta,\\
t_{\mathrm{yz,\,x}^2-y^2}[l,m,n] = \frac{3}{2}mn(l^2-m^2)\mathrm{dd}\sigma - mn\left(1+2(l^2-m^2)\right)\mathrm{dd}\pi + mn\left(1+\frac{1}{2}(l^2-m^2)\right)\mathrm{dd}\delta,\\
t_{\mathrm{zx,\,x}^2-y^2}[l,m,n] = \frac{3}{2}nl(l^2-m^2)\mathrm{dd}\sigma + nl\left(1-2(l^2-m^2)\right)\mathrm{dd}\pi - nl\left(1-\frac{1}{2}(l^2-m^2)\right)\mathrm{dd}\delta,\\
t_{\mathrm{xy},3z^2-r^2}[l,m,n] = \sqrt{3}lm\left(n^2-\frac{1}{2}(l^2+m^2)\right)\mathrm{dd}\sigma - 2\sqrt{3}lmn^2\mathrm{dd}\pi + \frac{1}{2}\sqrt{3}lm(1+n^2)\mathrm{dd}\delta,\\
t_{\mathrm{yz},3z^2-r^2}[l,m,n] = \sqrt{3}mn\left(n^2-\frac{1}{2}(l^2+m^2)\right)\mathrm{dd}\sigma + \sqrt{3}mn(l^2+m^2-n^2)\mathrm{dd}\pi - \frac{1}{2}\sqrt{3}mn(l^2+m^2)\mathrm{dd}\delta,\\
t_{\mathrm{zx},3z^2-r^2}[l,m,n] = \sqrt{3}ln\left(n^2-\frac{1}{2}(l^2+m^2)\right)\mathrm{dd}\sigma + \sqrt{3}ln(l^2+m^2-n^2)\mathrm{dd}\pi - \frac{1}{2}\sqrt{3}ln(l^2+m^2)\mathrm{dd}\delta,\\
t_{x^2-y^2,x^2-y^2}[l,m,n] = \frac{3}{4}(l^2-m^2)^2\mathrm{dd}\sigma + (l^2+m^2-(l^2-m^2)^2)\mathrm{dd}\pi + \left(n^2+\frac{1}{4}(l^2-m^2)^2\right)\mathrm{dd}\delta,\\
t_{3z^2-r^2,3z^2-r^2}[l,m,n] = \left(n^2-\frac{1}{2}(l^2+m^2)\right)^2\mathrm{dd}\sigma + 3n^2(l^2+m^2)\mathrm{dd}\pi + \frac{3}{4}(l^2+m^2)^2\mathrm{dd}\delta,\\
t_{x^2-y^2,3z^2-r^2}[l,m,n] = \frac{1}{2}\sqrt{3}(l^2-m^2)\left(n^2-\frac{1}{2}(l^2+m^2)\right)\mathrm{dd}\sigma + \sqrt{3}n^2(m^2-l^2)\mathrm{dd}\pi +\\
\frac{1}{4}\sqrt{3}(1+n^2)(l^2-m^2)\mathrm{dd}\delta.
\end{cases}
\tag{18}
$$

where $[l,m,n]$ are the direction cosines of the vector connecting the two atoms under consideration, while abα denotes the type α overlap integral between the orbitals a and b, respectively. As shown in the above relation, the Slater-Koster approach allows to express all the matrix elements (hopping integrals) between atomic orbitals in terms of a set of direction-dependent parameters. For two d orbitals on neighboring atoms, the overlap integrals depend on their relative orientation and are categorized into three types: ddσ, ddπ, ddδ. For the ddσ type, the orbitals overlap along the internuclear axis, leading to the strongest hybridization. For the ddπ type, overlap occurs when orbitals are oriented side-by-side, with a moderate hybridization. Finally, the ddδ type occurs when orbitals are oriented such that their lobes are misaligned in a way that produces a weaker overlap. Although not needed for deriving the effective Ti-Ti hopping through the Bi, the values of these parameters can be obtained by means of the Wannier approach within the DFT calculation discussed in the previous section. On the other hand, when dealing with the hopping term between a Ti atom and the bismuth one, we have to take into account the $d-p$ Slater-Koster coefficients:

$$
\begin{cases}
t_{\mathrm{x,\,xy}}[l,m,n] = \sqrt{3}l^2 m\mathrm{pd}\sigma + m(1-2l^2)\mathrm{pd}\pi, \\
t_{\mathrm{x,\,yz}}[l,m,n] = \sqrt{3}lmn\mathrm{pd}\sigma - 2lmn\mathrm{pd}\pi, \\
t_{\mathrm{x,\,zx}}[l,m,n] = \sqrt{3}l^2 n\mathrm{pd}\sigma + n(1-2l^2)\mathrm{pd}\pi, \\
t_{\mathrm{y,\,yz}}[l,m,n] = \sqrt{3}m^2 n\mathrm{pd}\sigma + n(1-2m^2)\mathrm{pd}\pi, \\
t_{\mathrm{y,\,zx}}[l,m,n] = \sqrt{3}mnl\mathrm{pd}\sigma - 2mnl\mathrm{pd}\pi, \\
t_{\mathrm{y,\,xy}}[l,m,n] = \sqrt{3}m^2 l\mathrm{pd}\sigma + l(1-2m^2)\mathrm{pd}\pi, \\
t_{\mathrm{z,\,zx}}[l,m,n] = \sqrt{3}n^2 l\mathrm{pd}\sigma + l(1-2n^2)\mathrm{pd}\pi, \\
t_{\mathrm{z,\,xy}}[l,m,n] = \sqrt{3}nlm\mathrm{pd}\sigma - 2nlm\mathrm{pd}\pi, \\
t_{\mathrm{z,\,yz}}[l,m,n] = \sqrt{3}n^2 m\mathrm{pd}\sigma + m(1-2n^2)\mathrm{pd}\pi, \\
t_{\mathrm{x,\,x^2}-y^2}[l,m,n] = \frac{\sqrt{3}}{2}l(l^2-m^2)\mathrm{pd}\sigma + l(1-l^2+m^2)\mathrm{pd}\pi, \\
t_{\mathrm{y,\,x^2}-y^2}[l,m,n] = \frac{\sqrt{3}}{2}m(l^2-m^2)\mathrm{pd}\sigma - m(1+l^2-m^2)\mathrm{pd}\pi, \\
t_{\mathrm{z,\,x^2}-y^2}[l,m,n] = \frac{\sqrt{3}}{2}n(l^2-m^2)\mathrm{pd}\sigma - n(l^2-m^2)\mathrm{pd}\pi, \\
t_{\mathrm{x,\,3}z^2\text{-}r^2}[l,m,n] = l(n^2-\frac{1}{2}(l^2+m^2))\mathrm{pd}\sigma - \sqrt{3}ln^2\mathrm{pd}\pi, \\
t_{\mathrm{y,\,3}z^2\text{-}r^2}[l,m,n] = m(n^2-\frac{1}{2}(l^2+m^2))\mathrm{pd}\sigma - \sqrt{3}mn^2\mathrm{pd}\pi, \\
t_{\mathrm{z,\,3}z^2\text{-}r^2}[l,m,n] = n(n^2-\frac{1}{2}(l^2+m^2))\mathrm{pd}\sigma + \sqrt{3}n(l^2+m^2)\mathrm{pd}\pi.
\end{cases}
\tag{19}
$$

By exploiting the properties of the Slater-Koster coefficients, we can directly build up the following hopping matrices $\hat{t}_{dd}$ and $\hat{t}_{pd}$:

$$
\begin{cases}
\left\{\hat{t}_{dd}\right\}_{a,b}[l,m,n] = t_{a,b}[l,m,n] \\
\left\{\hat{t}_{pd}\right\}_{c,b}[l,m,n] = t_{c,b}[l,m,n]
\end{cases}
, \tag{20}
$$

with $a,b = xy, yz, xz, x^2-y^2, 3z^2-r^2$ and $c = x, y, z$.

Neglecting the processes through the bismuth, we can reconstruct the tight-binding d-orbital Hamiltonian for the unit cell, which includes only the on-site terms and the direct hopping terms between Ti atoms. This part of the Hamiltonian can be expressed as

$$
\hat{h}_d = \begin{pmatrix} \hat{o}_1 & \hat{t}_{12} & \hat{t}_{13} \\ \hat{t}_{21} & \hat{o}_2 & \hat{t}_{23} \\ \hat{t}_{31} & \hat{t}_{32} & \hat{o}_3 \end{pmatrix}, \tag{21}
$$

where $\hat{o}_i$ is the on-site energy operator for the i-th site, while $\hat{t}_{ij}$ is the hopping one connecting the sites i and j. Since the Hamiltonian is hermitian, we have that $\hat{t}_{21} = \hat{t}^\dagger_{12}$, $\hat{t}_{31} = \hat{t}^\dagger_{13}$, and $\hat{t}_{32} = \hat{t}^\dagger_{23}$. Moreover, being the system invariant under rotation of $2\pi/3$ (and its multiples) around the z-axis, we infer that

$$
\begin{cases}
\hat{o}_1 = \mathcal{C}_{3z}\hat{o}_3\mathcal{C}^\dagger_{3z} \\
\hat{o}_2 = \mathcal{C}^\dagger_{3z}\hat{o}_3\mathcal{C}_{3z}
\end{cases}
\tag{22}
$$

and

$$\begin{cases} \hat{t}_{31} = \mathcal{C}_{3z}^{\dagger} \hat{t}_{12} \mathcal{C}_{3z} \\ \hat{t}_{23} = \mathcal{C}_{3z} \hat{t}_{12} \mathcal{C}_{3z}^{\dagger} \end{cases}, \tag{23}$$

where $\mathcal{C}_{3z} = \exp\left(i\frac{2\pi}{3}\hat{L}_z\right)$, and $\hat{\boldsymbol{L}} = \left\{\hat{L}_x, \hat{L}_y, \hat{L}_z\right\}$ is the orbital angular momentum operator. By providing the following representation for the $L = 2$ orbital angular moment operators

$$\hat{L}_x = \begin{pmatrix} 0 & 0 & -i & 0 & 0 \\ 0 & 0 & 0 & -i & -i\sqrt{3} \\ i & 0 & 0 & 0 & 0 \\ 0 & i & 0 & 0 & 0 \\ 0 & i\sqrt{3} & 0 & 0 & 0 \end{pmatrix}, \hat{L}_y = \begin{pmatrix} 0 & i & 0 & 0 & 0 \\ -i & 0 & 0 & 0 & 0 \\ 0 & 0 & 0 & -i & i\sqrt{3} \\ 0 & 0 & i & 0 & 0 \\ 0 & 0 & -i\sqrt{3} & 0 & 0 \end{pmatrix}, \tag{24}$$

$$\hat{L}_z = \begin{pmatrix} 0 & 0 & 0 & 2i & 0 \\ 0 & 0 & i & 0 & 0 \\ 0 & -i & 0 & 0 & 0 \\ -2i & 0 & 0 & 0 & 0 \\ 0 & 0 & 0 & 0 & 0 \end{pmatrix}, \tag{25}$$

it can be shown that

$$\begin{aligned} \hat{t}_{12} = &\frac{1}{12}\left(5\mathrm{dd}\delta - 8\mathrm{dd}\pi + 5\mathrm{dd}\sigma\right)\hat{L}_y^2 + \frac{1}{6}\mathrm{dd}\sigma\left(\hat{L}_x^2 + \hat{L}_z^2\right) \\ &+ \frac{1}{24}\left(-\mathrm{dd}\delta + 4\mathrm{dd}\pi - 3\mathrm{dd}\sigma\right)\left(\left\{\hat{L}_x^2, \hat{L}_y^2\right\} + \left\{\hat{L}_y^2, \hat{L}_z^2\right\}\right) \\ &+ ia\hat{L}_z + ib\left\{\hat{L}_y^2, \hat{L}_z\right\} + ic\left\{\hat{L}_x^2, \hat{L}_z\right\} \\ &+ d\hat{L}_z^2 + e\left\{\hat{L}_y^2, \hat{L}_z^2\right\} + f\left\{\hat{L}_x^2, \hat{L}_z^2\right\} \end{aligned}$$

and

$$\begin{aligned} \hat{o}_3 = &q_x\hat{L}_x^2 + q_y\hat{L}_y^2 + q_z\hat{L}_z^2 \\ &+ r_x\left\{\hat{L}_y^2, \hat{L}_z^2\right\} + r_y\left\{\hat{L}_x^2, \hat{L}_z^2\right\} + r_z\left\{\hat{L}_x^2, \hat{L}_y^2\right\}. \end{aligned}$$

We now include the central bismuth atom in our analysis. The whole Hamiltonian would be given by

$$\hat{\mathcal{H}} = \begin{pmatrix} \hat{o}_1 & \hat{t}_{12} & \hat{t}_{13} & \hat{t}_{1Bi} \\ \hat{t}_{21} & \hat{o}_2 & \hat{t}_{23} & \hat{t}_{2Bi} \\ \hat{t}_{31} & \hat{t}_{32} & \hat{o}_3 & \hat{t}_{3Bi} \\ \hat{t}_{1Bi}^{\dagger} & \hat{t}_{2Bi}^{\dagger} & \hat{t}_{3Bi}^{\dagger} & \hat{o}_{Bi} \end{pmatrix} = \begin{pmatrix} \hat{o}_1 & \hat{t}_{12} & \hat{t}_{13} & 0 \\ \hat{t}_{21} & \hat{o}_2 & \hat{t}_{23} & 0 \\ \hat{t}_{31} & \hat{t}_{32} & \hat{o}_3 & 0 \\ 0 & 0 & 0 & 0 \end{pmatrix} + \begin{pmatrix} 0 & 0 & 0 & \hat{t}_{1Bi} \\ 0 & 0 & 0 & \hat{t}_{2Bi} \\ 0 & 0 & 0 & \hat{t}_{3Bi} \\ \hat{t}_{1Bi}^{\dagger} & \hat{t}_{2Bi}^{\dagger} & \hat{t}_{3Bi}^{\dagger} & \hat{o}_{Bi} \end{pmatrix}, \tag{26}$$

with $\hat{t}_{jBi} = \hat{t}_{pd}\left(\boldsymbol{b}_{jBi}\right)$ (for $j = 1, 2, 3$), $\hat{o}_{Bi} = \lambda \hat{\boldsymbol{L}} \cdot \hat{\boldsymbol{\sigma}}$, and $\hat{\boldsymbol{\sigma}}$ as the Pauli matrix vector and the orbital moment refers to the operator within the p-orbitals manifold. To determine the effective d-orbital Hamiltonian $\hat{h}_{\text{eff}}$ for the Ti-Ti processes through the hybridization of the p-orbitals at the bismuth atom, we can apply the second-order perturbation theory. Specifically, we decompose the second term of the previous equation into a sum of two contributions:

$$\hat{\mathcal{H}}_0 = \begin{pmatrix} 0 & 0 & 0 & 0 \\ 0 & 0 & 0 & 0 \\ 0 & 0 & 0 & 0 \\ 0 & 0 & 0 & \hat{o}_{Bi} \end{pmatrix}, \hat{\mathcal{V}} = \begin{pmatrix} 0 & 0 & 0 & \hat{t}_{1Bi} \\ 0 & 0 & 0 & \hat{t}_{2Bi} \\ 0 & 0 & 0 & \hat{t}_{3Bi} \\ \hat{t}^{\dagger}_{1Bi} & \hat{t}^{\dagger}_{2Bi} & \hat{t}^{\dagger}_{3Bi} & 0 \end{pmatrix}, \tag{27}$$

where we treat $\hat{\mathcal{V}}$ as a perturbation with respect to $\hat{\mathcal{H}}_0$. Thus, we diagonalize $\hat{\mathcal{H}}_0$ and determine its (degenerate) eigenvalues: $E_0 = 0, -2\lambda, \lambda$. In addition, the eigenvectors associated to $E_0 = 0$ coincides with the d-orbitals centered on the Ti atoms. This implies that, by applying the second-order degenerate perturbation theory, one can obtain the correction $\hat{h}_{Bi}$ to $\hat{h}_d$ arising from the hybridization effects induced by the presence of the Bi atom within the cell. In detail, we have that

$$\hat{h}_{Bi} = \sum_i -\frac{\hat{\mathcal{V}} \left|v_i\right\rangle \left\langle v_i\right| \hat{\mathcal{V}}}{E_i}, \tag{28}$$

where the sum is extended over all the eigenstates $\left|v_i\right\rangle$ of $\hat{\mathcal{H}}_0$ such that $\hat{\mathcal{H}}_0 \left|v_i\right\rangle = E_i \left|v_i\right\rangle$ with $E_i \neq E_0$. It follows that

$$\hat{h}_{\text{eff}} = \hat{h}_d + \hat{h}_{Bi}, \tag{29}$$

where $\hat{h}_{Bi}$ can be put in the following form:

$$\hat{h}_{Bi} = \begin{pmatrix} \hat{\tilde{o}}_1 & \hat{\tilde{t}}_{12} & \hat{\tilde{t}}_{13} \\ \hat{\tilde{t}}^{\dagger}_{12} & \hat{\tilde{o}}_2 & \hat{\tilde{t}}_{23} \\ \hat{\tilde{t}}^{\dagger}_{13} & \hat{\tilde{t}}^{\dagger}_{23} & \hat{\tilde{o}}_3 \end{pmatrix}. \tag{30}$$

As done previously, we can exploit the symmetry argument to determine the matrix elements. Then, one can express $\hat{\tilde{o}}_1$ and $\hat{\tilde{t}}_{12}$ in a proper operator basis $\left(\hat{\mathcal{A}}_1, \hat{\mathcal{A}}_2, \ldots, \hat{\mathcal{A}}_{100}\right)$, i.e.,

$$\begin{cases} \hat{\tilde{o}}_1 = \sum_{i=1}^{100} \alpha_i \hat{\mathcal{A}}_i \\ \hat{\tilde{t}}_{12} = \sum_{i=1}^{100} \beta_i \hat{\mathcal{A}}_i \end{cases}. \tag{31}$$

Focusing only on the orbital degrees of freedom, we can choose the following set of operators:

$$\begin{pmatrix} \hat{I}_5, \hat{L}_x, \hat{L}_y, \hat{L}_z, \hat{L}_x^2, \hat{L}_y^2, \left\{\hat{L}_x, \hat{L}_y\right\}, \left\{\hat{L}_y, \hat{L}_z\right\}, \left\{\hat{L}_z, \hat{L}_x\right\}, \\ \hat{L}_x^3, \hat{L}_y^3, \hat{L}_z^3, \left\{\hat{L}_x^2, \hat{L}_y\right\}, \left\{\hat{L}_y^2, \hat{L}_z\right\}, \left\{\hat{L}_z^2, \hat{L}_x\right\}, \left\{\left\{\hat{L}_x, \hat{L}_y\right\}, \hat{L}_z\right\}, \\ \hat{L}_x^4, \hat{L}_y^4, \hat{L}_z^4, \left\{\hat{L}_x, \hat{L}_y^3\right\}, \left\{\hat{L}_y, \hat{L}_z^3\right\}, \left\{\hat{L}_z, \hat{L}_x^3\right\}, \left\{\hat{L}_y, \hat{L}_x^3\right\}, \left\{\hat{L}_z, \hat{L}_y^3\right\}, \left\{\hat{L}_x, \hat{L}_z^3\right\} \end{pmatrix}, \tag{32}$$

with $\hat{I}_5$ as the identity matrix in the $L = 2$ orbital space. Now, we can tensor-multiply each term in this operator set by $\hat{I}_2 = \hat{\sigma}^2_{i=x,y,z}$, $\hat{\sigma}_x$, $\hat{\sigma}_y$, and $\hat{\sigma}_z$, respectively, thereby obtaining the desired operator basis $\left(\hat{\mathcal{A}}_{i=1,\ldots,100}\right)$. At this stage, our aim is to demonstrate that the presence of the bismuth atom at the center of the triangular plaquette gives rise to cross spin-orbit coupling terms in the effective d-orbital Hamiltonian for the Ti atoms. Thus, we consider the expansions for $\hat{\hat{o}}_1$ and $\hat{\hat{t}}_{12}$ up to the $\hat{L}^2$ terms, neglecting higher-order contributions in the orbital angular momentum. Furthermore, for sake of clarity, we fix the polar angle to a representative value of $\theta = \pi/3$, capturing the out-of-plane spatial configuration of the Bi atom. Starting with the on-site matrix term $\hat{\hat{o}}_1$, we infer that

$$\begin{aligned}\hat{\hat{o}}_1 &= a_1 + a_2\hat{L}_x^2 + a_3\hat{L}_y^2 + a_4\left\{\hat{L}_x, \hat{L}_y\right\} + a_5\left\{\hat{L}_y, \hat{L}_z\right\} + a_6\left\{\hat{L}_z, \hat{L}_x\right\} \\ &+\hat{\sigma}_x\left[a_7\hat{L}_x + a_8\hat{L}_y + a_9\hat{L}_z\right] + \hat{\sigma}_y\left[a_{10}\hat{L}_x + a_{11}\hat{L}_y + a_{12}\hat{L}_z\right] \\ &+\hat{\sigma}_z\left[a_{13}\hat{L}_x + a_{14}\hat{L}_y + a_{15}\hat{L}_z\right] + \mathcal{O}\left(\hat{L}^3\right).\end{aligned} \tag{33}$$

Specifically, the coefficients of the cross spin-orbit coupling terms are given by

$a_8/\text{pd}\pi$	$a_9/\text{pd}\pi$	$a_{10}/\text{pd}\pi$	$a_{12}/\text{pd}\pi$	$a_{13}/\text{pd}\pi$	$a_{14}/\text{pd}\pi$
$\frac{3\left(6\sqrt{3}\text{pd}\pi-73\text{pd}\sigma\right)}{2048\lambda}$	$\frac{2\sqrt{3}\text{pd}\pi-33\text{pd}\sigma}{96\lambda}$	$\frac{3\left(122\sqrt{3}\text{pd}\pi+137\text{pd}\sigma\right)}{2048\lambda}$	$\frac{2\text{pd}\pi+\sqrt{3}\text{pd}\sigma}{32\lambda}$	$\frac{-122\sqrt{3}\text{pd}\pi+247\text{pd}\sigma}{1024\lambda}$	$\frac{-18\text{pd}\pi-311\sqrt{3}\text{pd}\sigma}{1024\lambda}$

On the other hand, we find that the hopping matrix term $\hat{\hat{t}}_{12}$ can be written as

$$\begin{aligned}\hat{\hat{t}}_{12} =& b_1 + ib_2\hat{L}_x + ib_3\hat{L}_z + b_4\hat{L}_x^2 + b_5\hat{L}_y^2 + b_6\left\{\hat{L}_z, \hat{L}_x\right\} \\ &+\hat{\sigma}_x\left[ib_7 + b_8\hat{L}_x + b_9\hat{L}_z + ib_{10}\hat{L}_x^2 + ib_{11}\hat{L}_y^2 + ib_{12}\left\{\hat{L}_z, \hat{L}_x\right\}\right] \\ &+\hat{\sigma}_y\left[ib_{13}\left\{\hat{L}_y, \hat{L}_z\right\} + ib_{14}\left\{\hat{L}_x, \hat{L}_y\right\} + b_{15}\hat{L}_y\right] \\ &+\hat{\sigma}_z\left[ib_{16} + b_{17}\hat{L}_x + b_{18}\hat{L}_z + ib_{19}\hat{L}_x^2 + ib_{20}\hat{L}_y^2 + ib_{21}\left\{\hat{L}_z, \hat{L}_x\right\}\right] + \mathcal{O}\left(\hat{L}^3\right).\end{aligned} \tag{34}$$

In this case, defining $\text{p}\tilde{\text{d}}\sigma = \text{pd}\sigma/\text{pd}\pi$, the coefficients of the terms involving cross spin-orbit couplings are

$b_9/\text{pd}\pi^2$	$b_{11}/\text{pd}\pi^2$	$b_{12}/\text{pd}\pi^2$	$b_{13}/\text{pd}\pi^2$	$b_{14}/\text{pd}\pi^2$
$\frac{292\sqrt{3}-3036\text{p}\tilde{\text{d}}\sigma-621\sqrt{3}\text{p}\tilde{\text{d}}\sigma^2}{12288\lambda}$	$\frac{2397\text{p}\tilde{\text{d}}\sigma^2-1476-860\sqrt{3}\text{p}\tilde{\text{d}}\sigma}{8192\lambda}$	$\frac{3\left(96\sqrt{3}+2\text{p}\tilde{\text{d}}\sigma-57\sqrt{3}\text{p}\tilde{\text{d}}\sigma^2\right)}{2048\lambda}$	$\frac{63\text{p}\tilde{\text{d}}\sigma-10\sqrt{3}}{1024\lambda}$	$\frac{14+\sqrt{3}\text{p}\tilde{\text{d}}\sigma}{256\lambda}$

$b_{17}/\text{pd}\pi^2$	$b_{19}/\text{pd}\pi^2$	$b_{20}/\text{pd}\pi^2$	$b_{21}/\text{pd}\pi^2$
$\frac{764\sqrt{3}+1076\text{p}\tilde{\text{d}}\sigma-855\sqrt{3}\text{p}\tilde{\text{d}}\sigma^2}{8192\lambda}$	$\frac{3\left(1604\text{p}\tilde{\text{d}}\sigma-89\sqrt{3}\text{p}\tilde{\text{d}}\sigma^2-1292\sqrt{3}\right)}{16384\lambda}$	$\frac{2397\sqrt{3}\text{p}\tilde{\text{d}}\sigma^2-6340\sqrt{3}+3180\text{p}\tilde{\text{d}}\sigma}{16384\lambda}$	$\frac{1156-108\sqrt{3}\text{p}\tilde{\text{d}}\sigma-513\text{p}\tilde{\text{d}}\sigma^2}{4096\lambda}$

Considering that the ratio between pdπ and pdσ is approximately one, with both values around 1 eV, and noting that the atomic spin-orbit coupling of Bi, λ, is roughly 1.5 eV, we estimate that the magnitudes of the b coefficients listed in the above table are on the order of 0.2 eV. Therefore, because of these electronic processes, it is reasonable to anticipate that phases which break time-reversal symmetry and display spin-orbital cross correlations can gain kinetic energy, making them more energetically favorable compared to phases characterized solely by orbital quadrupoles.

VI. ARPES AND SPIN-ARPES DATA

ARPES and Spin-ARPES were conducted at both the CASSIOPEE laboratory of SOLEIL (Paris) and the APE-LE laboratory of Elettra (Trieste). The samples were prepared by mounting them in a controlled glovebox environment and top-posting them with ceramic elements affixed using silver epoxy. The silver epoxy was cured within the glovebox at 100°C for one hour, after which the samples were transferred into ultra-high vacuum (UHV) conditions, cooled to 15 K, and cleaved *in situ*.

The samples were meticulously aligned along high-symmetry directions, employing various light polarizations and photon energies. Through the analysis of the multi-polarized Fermi surfaces, we achieved azimuthal alignment with an accuracy of 1 degree. The bulk Γ point, the focal point of our spin-ARPES investigation, was estimated to occur at approximately 65 eV photon energy. However, as detailed below, all observed bands displayed pronounced two-dimensional characteristics, exhibiting nearly dispersionless features.

We commenced our investigation by focusing on spin-integrated ARPES, electronic dimensionality, and polarization control. Spectra were systematically acquired with the analyser slit precisely aligned along both the $\Gamma - M$ direction of the Brillouin zone, across a comprehensive range of photon energies (See Fig. 6 and Fig. 7, for linear and circular polarizations respectively). This methodological approach facilitated a thorough exploration of the system's electronic dimensionality, enabling the identification of any deviations that could indicate three-dimensional dispersion. Notably, the electronic states consistently exhibited a fully two-dimensional character, irrespective of the polarization employed. The same conclusion is drawn by looking at the electronic states below the Fermi level, corroborating the genuine two-dimensional nature of the bands investigated. Furthermore, the extracted circular dichroism at various photon energies exhibits no anomalies along this direction, with only a minimal residual signal observed at the zone center. This finding reinforces the assertion presented in the main text, where the circular dichroism of the spin-integrated signal was shown to be negligible, given the limitations of the experimental resolution.

Another compelling aspect that substantiates the two-dimensional electronic behavior is the lack of significant variations in the dispersion of the system's Fermi surface and constant energy maps. Specifically, while a general redistribution of spectral weight occurs, the fermiology identified across a broad photon energy range exhibits remarkable consistency. This consistency is illustrated in Fig. 9-10-11, where no substantial changes are observed across different photon energies. Note that the Γ point has been estimated by comparing our data with previous works and by extracting it from the c-axis parameters ($c = 9.2062$ Å, and $V_0 = 6$ eV). Here, for completeness we report Fermi surface maps and constant energy contours collected at various photon energies.

We now focus on the Spin-ARPES aspect of our study. Initially, using conventional ARPES, we aligned the samples such that the incident light lies within one of the mirror planes of the crystal. In this configuration ($K \rightarrow K$ and $M \rightarrow M$), the matrix elements are well-defined, enabling us to effectively disentangle the contributions from geometrical matrix elements. Additionally, we concentrated our investigation at the Γ point, where the geometrical matrix elements are inherently zero. This strategic approach significantly minimizes the potential for artefacts in our

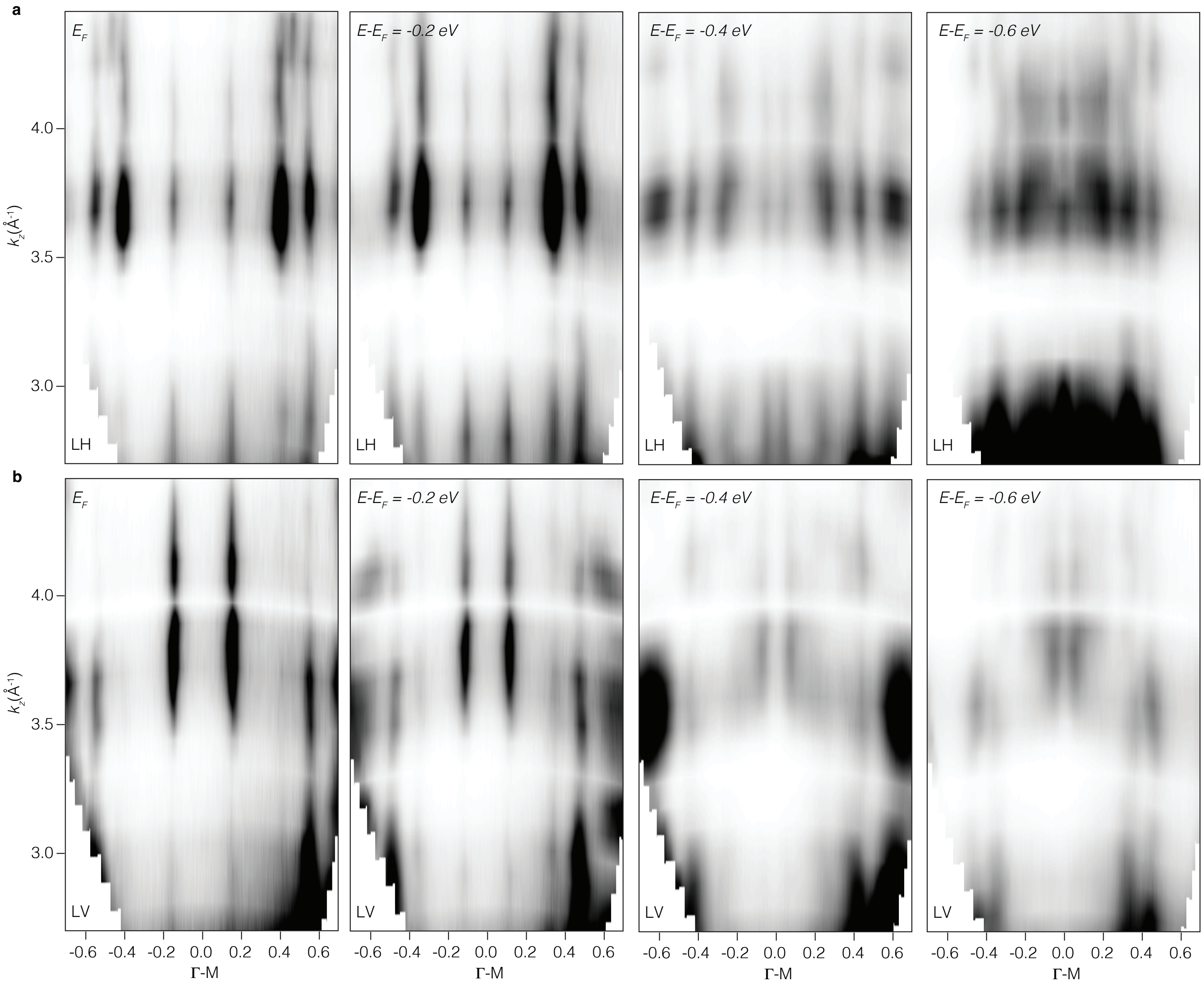

FIG. 6. **a.** Linear horizontal and **b.** vertical polarizations spectra collected at various photon energies and covering multiple Brillouin zones in k_z. The absence of three-dimensionality is confirmed by the stripe-like behaviour shown by the data, which is also independent on the binding energy measured.

measurements.

To acquire spectra at Γ, we collected photoelectron data at various angles using the spin detector and constructed the ARPES image from these measurements. In Fig. 12, we present the spectra obtained solely with the spin detector in various light-polarization configurations, depicted here with a thick grid of points for clarity. The resulting dispersion now reveals the electronic bands as ARPES typically does, allowing us to precisely identify the angle corresponding to the center of the Brillouin zone.

With the Γ point identified, we proceeded to acquire spin-resolved energy distribution curves (EDCs) at the zero angle, capturing both spin channels (positive and negative) while employing both right- and left-handed circular polarizations. The spin-ARPES data were normalized by enforcing a uniform background across all polarization and

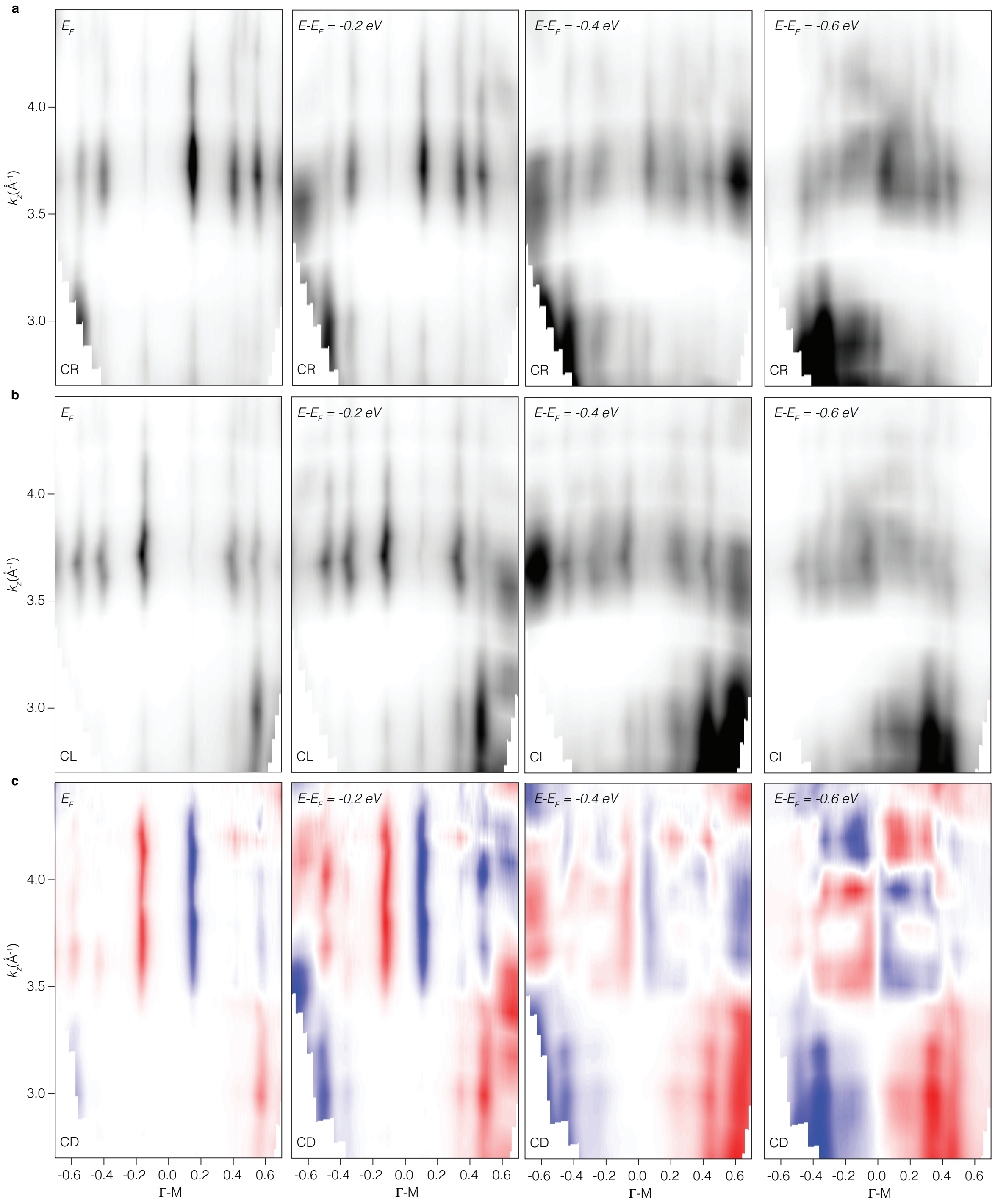

FIG. 7. **a.** Circular right, **b.** left polarizations, and **c.**Dichroism spectra were collected across various photon energies, encompassing multiple Brillouin zones in k_z. Consistent with the previously presented linear polarizations, the absence of three-dimensional behavior is again observed. Notably, despite the variation in photon energy, the dichroism results are distinctly defined at both positive and negative momenta, with minimal residual contributions at the center of the Brillouin zone. This observation is in alignment with the main text findings and further reinforces the robustness of this effect across different photon energies.

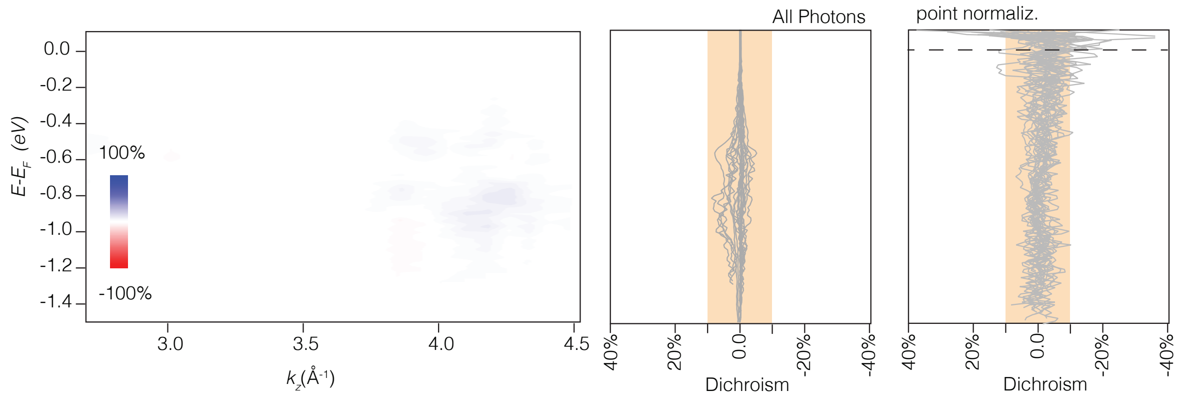

FIG. 8. EDCs collected with circular dichroism at several photon energies. As one can see, the maximum deviation occurs with circa 10% of maximum residual. To the right the single EDCs are reported: the first left line-graph is the standard dichroism obtained as the difference between right and left helicity. The panel to the right shows the EDCs but after the difference in helicities is divided by the sum of them. The increased noise above and in proximity of Fermi is normal and due to a division by a number which is nearly zero. Both methods show clearly the corroboration of a small circular dichroism.

spin channels, ensuring that the signal above the Fermi level was systematically nullified. Following this procedure, we extracted the polarization for each spin species ($\sigma_z = +1$ and $\sigma_z = -1$), incorporating an efficiency factor ($S = 0.3$) derived from the Sherman function. Consequently, for each circular polarization, the spin polarization P is given by:

$$P_{C^{+,-}} = \frac{1}{S}\frac{C_\uparrow^{+,-} - C_\downarrow^{+,-}}{C_\uparrow^{+,-} + C_\downarrow^{+,-}}$$

Upon extraction of the polarization, the EDCs were recalculated to account for the Sherman function, allowing us to express the true spin-resolved intensities as:

$$C_{TRUE}^{+,-}(\uparrow) = \frac{C_\uparrow^{+,-} + C_\downarrow^{+,-}}{2} * (1 + P_{C^{+,-}})$$

$$C_{TRUE}^{+,-}(\downarrow) = \frac{C_\uparrow^{+,-} + C_\downarrow^{+,-}}{2} * (1 - P_{C^{+,-}})$$

These results correspond to the analysis presented in Fig. 2d of the main text. For the presentation of Fig. 2c, we opted for a representation that provides an intuitive understanding of the degree of spin polarization and dichroic signal. Specifically, the spin-resolved dichroism reflects the percentage of circular polarization for each spin channel, while the spin-integrated dichroism illustrates the residual percentage of the total dichroic signal, where the spin is fully integrated (and thus irrelevant and all summed up). This approach enables for the spin-integrated curves a direct comparison with dichroism data collected via standard ARPES.

For completeness, we present the results of alternative analysis approaches in Fig.13. Specifically, we show with normalization the data to their background (as in the main text), then with application of a background subtraction using the Shirley method, and finally, an additional normalization to the Fermi edge. The most notable observation is that the curves differ significantly, strongly indicating the anomalous spin-optical effect, which remains

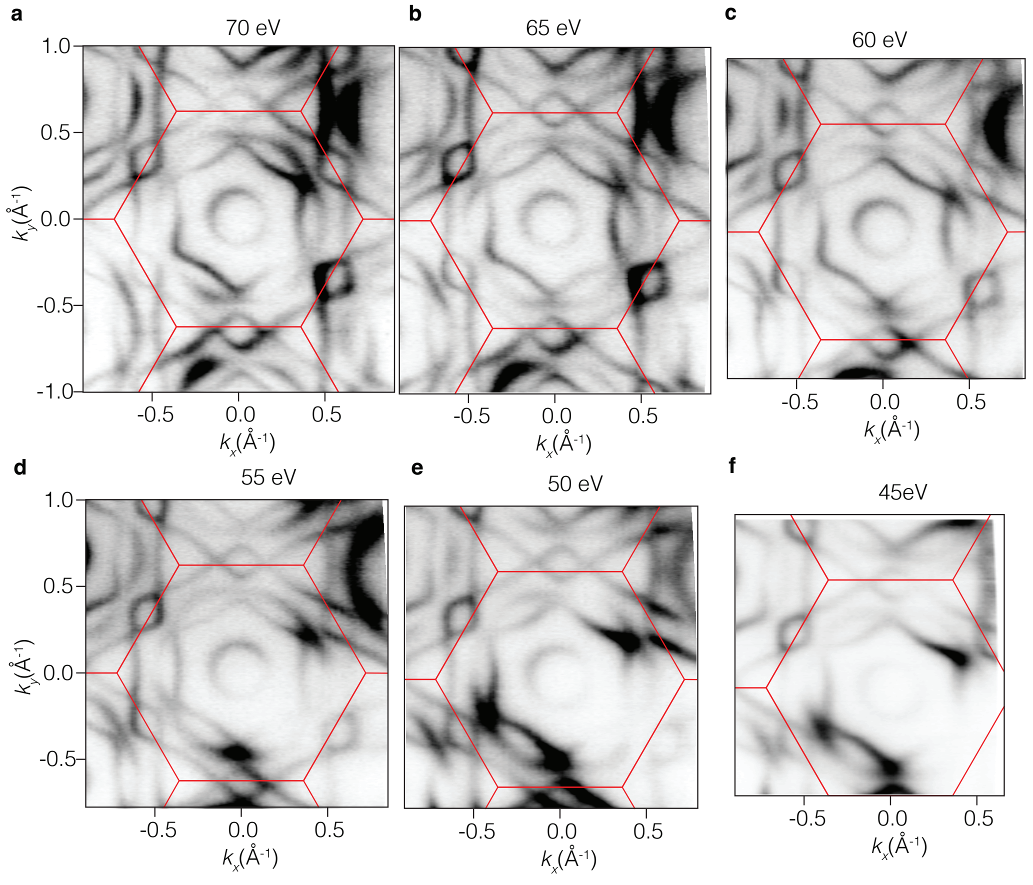

FIG. 9. Fermi surface map collected with linear horizontal polarization from 70 eV to 45 eV in step of 5 eV.

unaffected by the choice of normalization. For completeness we also extract the polarization of the dichroism and spin-dichroism for Fig.13b-c and we show this in Fig.14: we note that the outcome is entirely independent of the normalization method and procedure applied. The larger oscillations are expected: when dividing by the sum to obtain the percentage ratio, the division by values near zero amplifies the signal. However, the associated uncertainty scales proportionally as well.

A thorough evaluation of k_z-dependent matrix element effects is essential when interpreting spin- and circular-dichroism-resolved ARPES data, particularly due to the influence of final-state interference phenomena such as the Daimon effect. This effect, arising from coherent scattering in the final state, can generate artificial dichroic and spin-polarized signals even in centrosymmetric, non-magnetic materials. To assess this possibility, we carried out photon energy-dependent measurements, which revealed intricate variations in the dichroic response across momentum space, including occasional sign reversals. Nonetheless, a key result emerges at the Γ point ($\mathbf{k} = 0$), where the circular dichroism remains consistently suppressed—below 5%—over a wide photon energy range covering several Brillouin zones (as in supplementary Fig. 14). Since the Daimon effect is known to diminish as the dichroism approaches zero, and because the spin polarization also vanishes at this location, we identify the finite $L \cdot S$ signal as an intrinsic property of the initial state rather than an artifact of the photoemission process. This situation—where both L and S are independently negligible while their scalar product remains finite—is incompatible with a purely

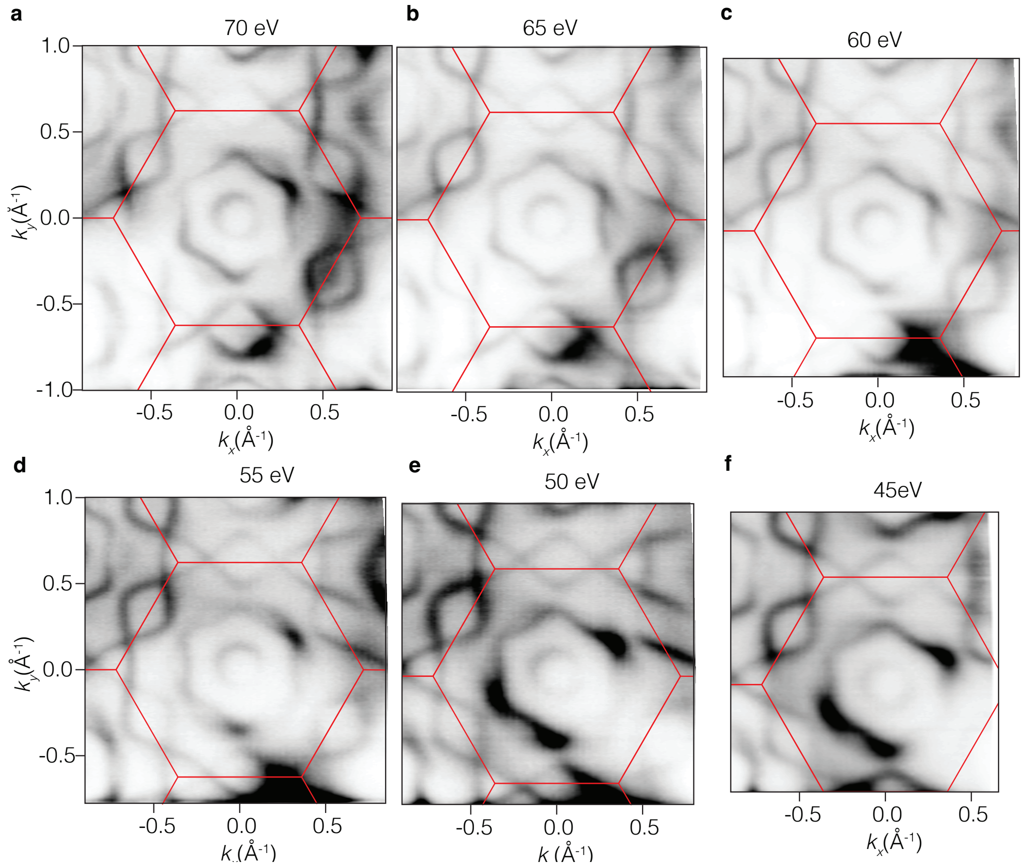

FIG. 10. Constant energy contour (200 meV below the Fermi level) collected with linear horizontal polarization from 70 eV to 45 eV in step of 5 eV.

final-state origin and points instead to a genuine bulk effect driven by spin-orbit coupling. The choice to probe the Γ point, where time-reversal and inversion symmetries impose strong constraints, further reduces the likelihood of spurious contributions from surface or geometric effects.

Importantly, from a theoretical point of view, the time reversal symmetry breaking should manifest also via analyzing the spin signal alone, without the need of circular polarization, even if its amplitude is expected to be significantly smaller than that one generated by spin-orbital correlations. In Fig.15 we show all the various degrees of freedom compared at the Γ point. In particular, left and right circular polarization show negligible difference and circularly polarized spin-resolved measurements are instead largely distinct between each others, as shown also in the main text. The spin-resolved measurements (red and green lines - no circular polarization) show a difference in one of the peaks and negligible difference in the rest of the peaks measured. While this might be compatible with time-reversal symmetry breaking as predicted by theory, we cannot exclude that this state might come from a band aging extremely rapidly within the time-frame of our experiment (we performed all measurements within 30 minutes of total time to avoid this and such a phenomenon was not observed by ARPES within the time frame used and also in reversed ordered for up and down species). Thus, even if precautions were taken, we cannot exclude that the origin of that peak might be an artifact and this would need an investigation by itself. However, the phenomenology observed involves also the other spectral features, for which spin is negligible or significantly much smaller than the spin-helical

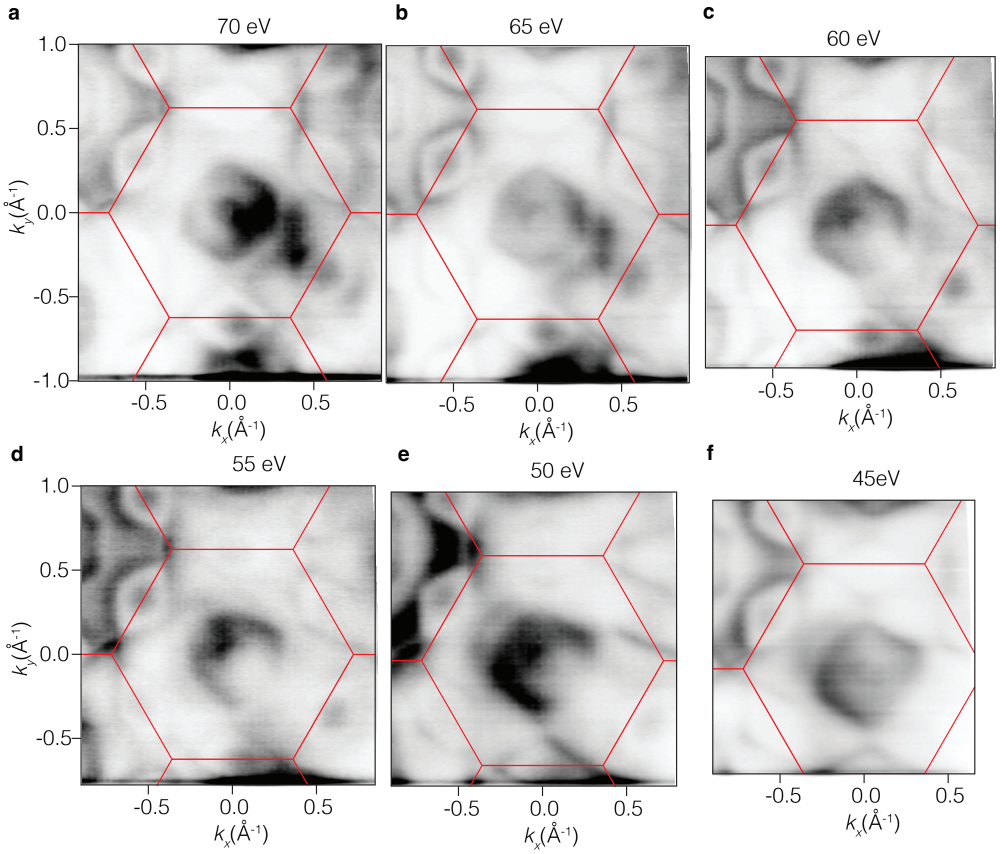

FIG. 11. Constant energy contour (400 meV below the Fermi level) collected with linear horizontal polarization from 70 eV to 45 eV in step of 5 eV.

signals, corroborating the validity of the match between experiment and theory.

VII. ZERO FIELD AND TRANSVERSE FIELD μSR RESULTS IN CSTI$_3$BI$_5$

A. Experimental results

Muon spin rotation and relaxation experiments have been conducted on a powdered sample mounted on a sample holder covered with a Kapton mask to reduce the background signal. The experiments were performed using the EMU spectrometer at ISIS, STFC Rutherford Appleton Laboratory, United Kingdom [7, 8].

The zero field asymmetry spectra obtained at 5 K are shown in Fig. 16a. A clear departure from the standard Gaussian Kubo-Toyabe trend is observed. This can be attributed, in general, to either the anisotropy of the nuclear fields [9], or electronic fluctuations or dynamical effects due to quantum tunneling. Interestingly, with increasing temperature, the ZF-spectrum evolves toward a marked dynamic behavior, which most likely originates from classical muon diffusion in the lattice. The analysis detailed below shows that the hopping rate v of the muon follows and activated behaviors, as shown in the inset of Fig. 16a, with an activation energy about 8 meV.

In order to clarify the observed behavior, we have performed first-principles simulations using the Density Func-

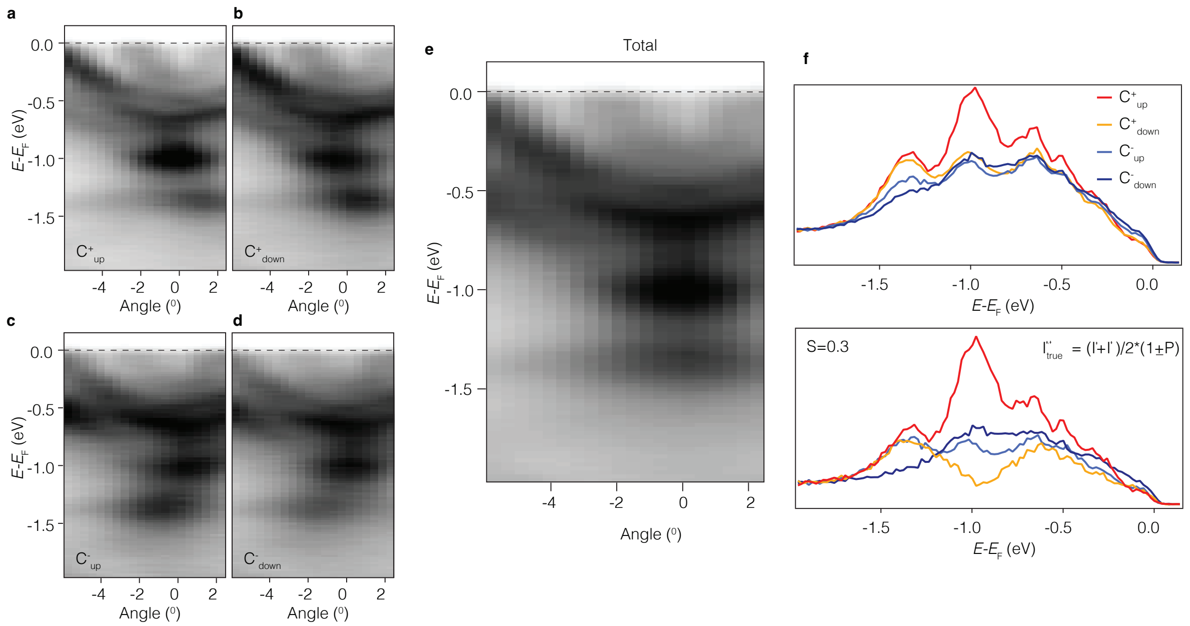

FIG. 12. ARPES map reconstructed by using the spin detectors: by collecting EDCs it is possible to build the maps in each polarization and spin and understand the Γ point. Data have been collected as **a** $C^+_\uparrow$, **b** $C^+_\downarrow$, **c** $C^-_\uparrow$, and **d** $C^-_\downarrow$. Then **e** the total signal which gives a better overview of the centre of the zone is reconstructed. **f** EDCs at Γ extracted from the raw spectra are shown in the upper panel (note the effect is visible already there) and the ones after Sherman function applied for each helicity and including the spin species at the Γ point.

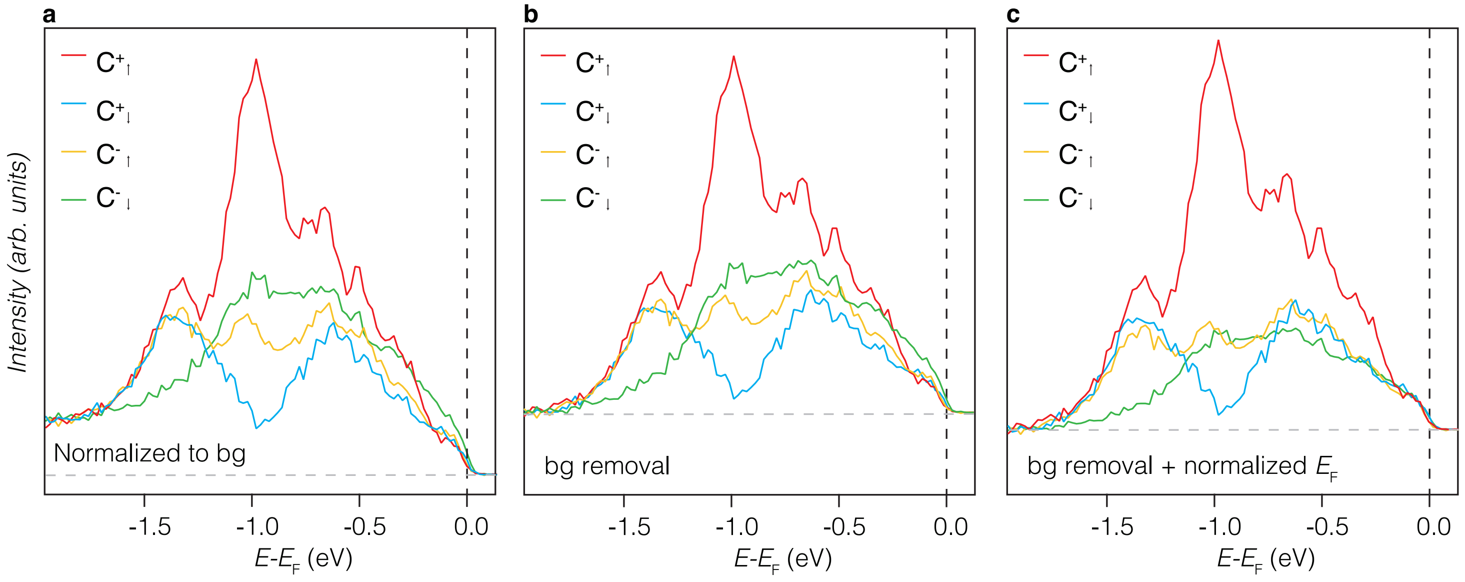

FIG. 13. EDCs collected from the Γ point after **a** normalization to the background as in the main text, but here reported on the same graph with the real relative intensity. **b** The same but after a Shirley background removal and **c** after forcing the Fermi edge to be the same.

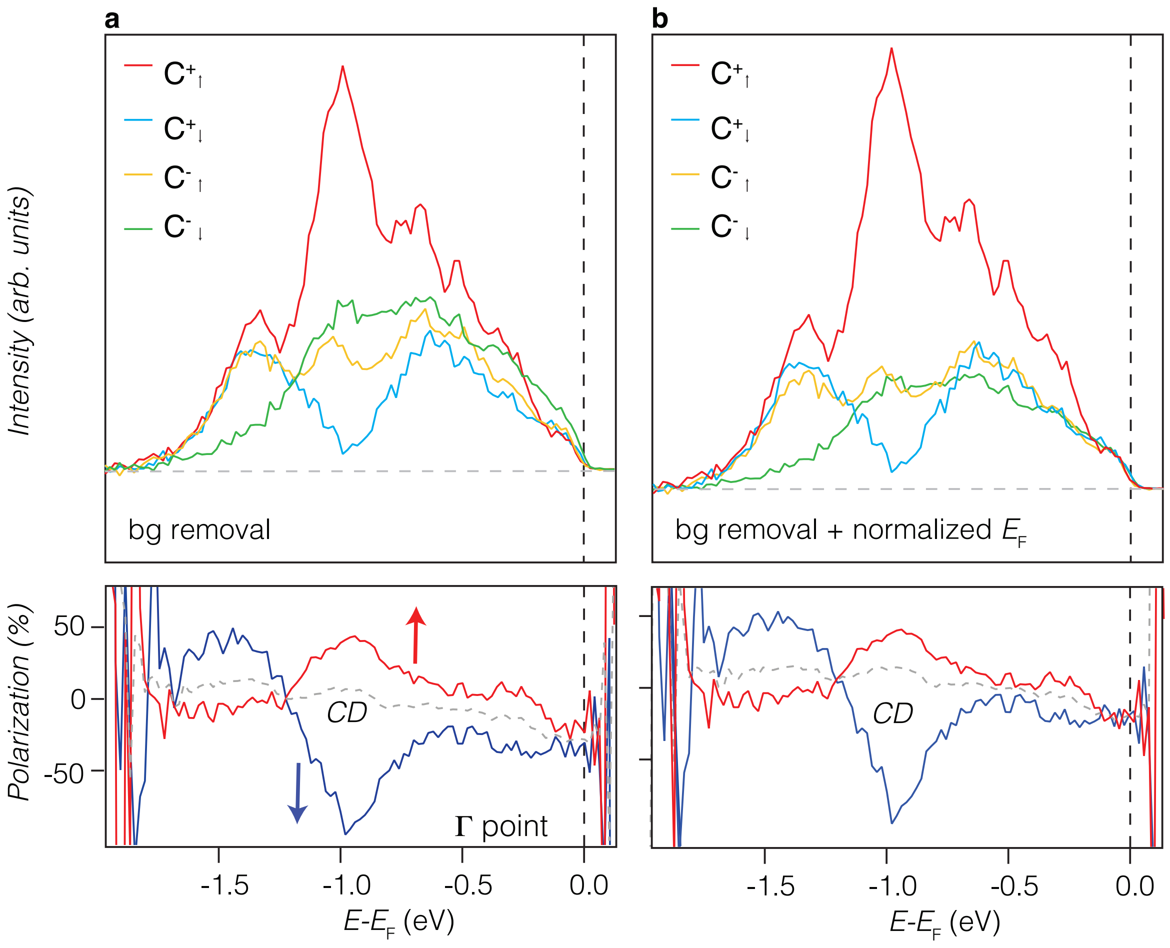

FIG. 14. EDCs collected from the Γ point after normalization to the background and **b** after a Shirley background removal and **c** after forcing the Fermi edge to be the same. Below each panel the extracted spin-dichroic response and canonical circular dichroism are reported. Both methodologies used bring to a similar conclusion. Note that by normalizing around the Fermi level, gives an offset to all the signals.

tional Theory (DFT)+μ approach [10, 11]. The two lowest energy and almost degenerate stable muon sites are shown in Fig. 16c while panel b) shows the predicted relaxation rates obtained after accurate evaluation of perturbation effects introduced by the muon (see SI for details). Both A and B sites produce a relaxation rate that is slightly slower than the one observed experimentally. Perfect agreement can be obtained if a large perturbation is induced on the neighboring Bi nuclei, although the expected reduction of the electric field gradient (EFG) is physically unsound. Further analysis of the quantum effects for the muon in this system is likely required to improve the agreement with the experiment. Despite the small discrepancy, our results allow us to conclude that no static magnetic fields larger than 0.25 mT can be present at the muon site. This would potentially correspond to a dipolar contribution from Ti atoms originating from a magnetic moment smaller than 2×10^{-3} μ_B.

The zero field muon spin relaxation (μSR) asymmetry spectra for the lowest measured temperature 5.0 K along with 65 K and 180 K are shown in Fig. 17. The low-temperature data cannot be completely understood by the static Kubo-Toyabe (KT) function. This is due to the approximate nature of this phenomenological fitting function, which

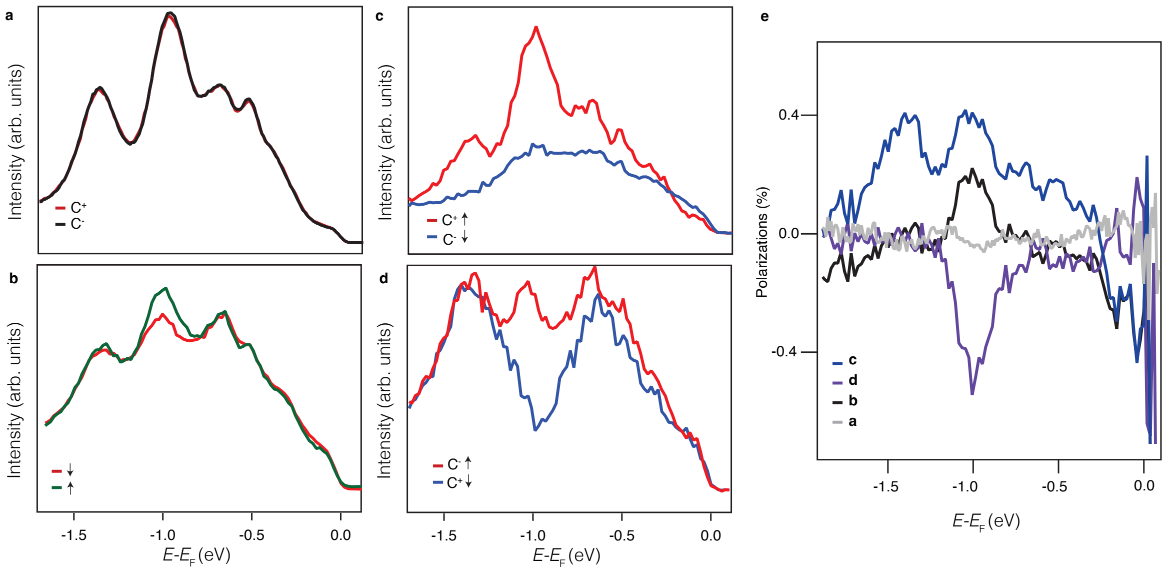

FIG. 15. Comparison between spin and dichroism and spin-dichroism and their relative polarization. **a** Spin-integrated circular positive and negative polarization. The integration performed is of 1 degree and extracted before momentum warping to compare it properly to the spin-resolved data. **b** Spin-integrated spectra collected with unpolarized light (we summed up circular positive and negative) showing that the spin collected at this point is non-zero but very small and the raw curve show a 5% difference. **c** Circular positive and up spin specie and circular negative with species spin down compared and **d** their mixed compositions. **e** Percentages in comparison: blue and purple lines show the percentage of spin-dichroic signal meaning the percentage of time-reversal symmetry breaking. The gray curve shows the dichroic difference, which is very small and negligible, while the black is the spin-resolved signal without dichroism. This is more than 4 times smaller and reaches approximately 15% after dividing it by 0.3 for accounting for a Sherman function. In summary the combined spin and dichroic signal show an amplification.

does not take into account quantum contributions and anisotropies in the nuclear field distribution [12].

Moreover, the temperature evolution of spectra depicts the slow depolarization with increasing temperature. To understand the temperature evolution we fit the experimental data using the dynamic Kubo-Toyabe expression, which follows as,

$$G_z(t) = g_z(t)\exp(-vut) + \nu \int_0^t g_z(\tau)\exp(-vut)G_z(t-\tau)\,d\tau \tag{35}$$

where $g_z(t)$ is the static KT function, and ν is the muon hopping rate. For zero-field, the static KT function accounts for the randomly distributed Gaussian nuclear magnetic moment and can be written as,

$$g_z(t) = \mathrm{A}\left[\frac{1}{3} + \frac{2}{3}\left(1-\Delta^2 t^2\right) e^{-\frac{1}{2}\Delta^2 t^2}\right] \tag{36}$$

here Δ represents the second moment of the local field distribution. For ZF fitting, the Δ parameter was fixed to the low-temperature value 0.19498 μs^{-1}, while the hopping rate was set free. The temperature dependence of the hopping parameter ν, in terms of its logarithmic value as a function of inverse temperature, is shown in Fig. 17. A thermal activation law is used to evaluate the variation of ν, written as,

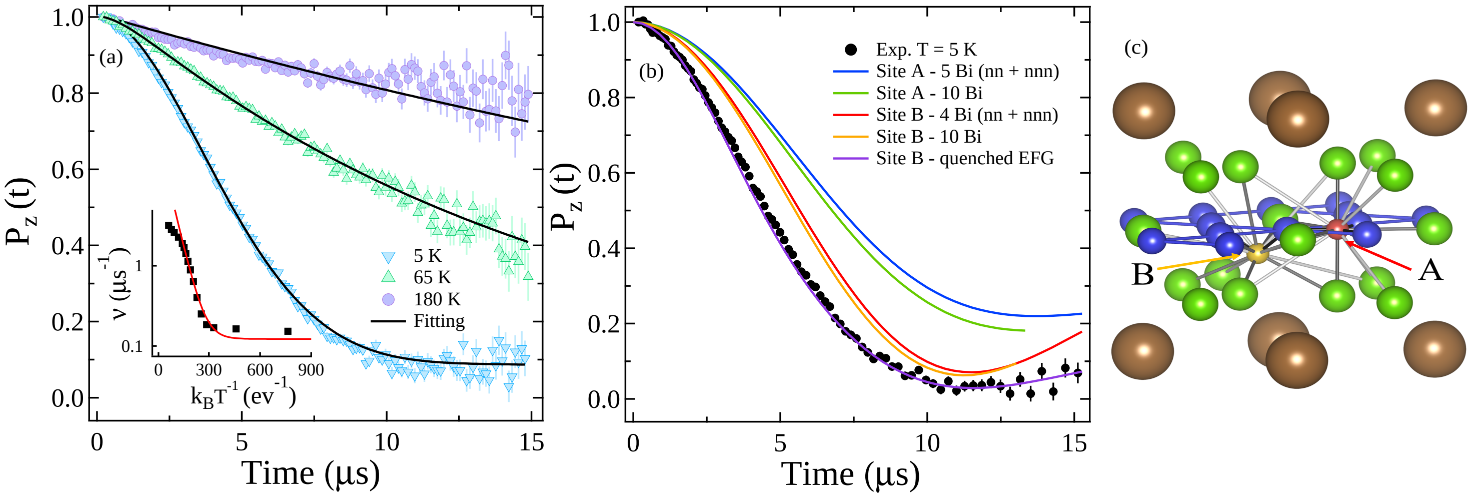

FIG. 16. (a) Experimental data at different temperatures are shown, with fitting represented by solid black line. Inset show the Arrhenius law fit for hopping rate ν. (b) Zero-field experimental data at 5 K is compared with the predicted polarisation function for two sites A and B obtained for different sets of nearest neighbors (nn). (c) The two muon sites, site A and B, represented by red and yellow solid spheres, respectively, were obtained using the DFT+μ method with Cs, Ti, and Bi atoms depicted as brown, blue and green spheres, respectively.

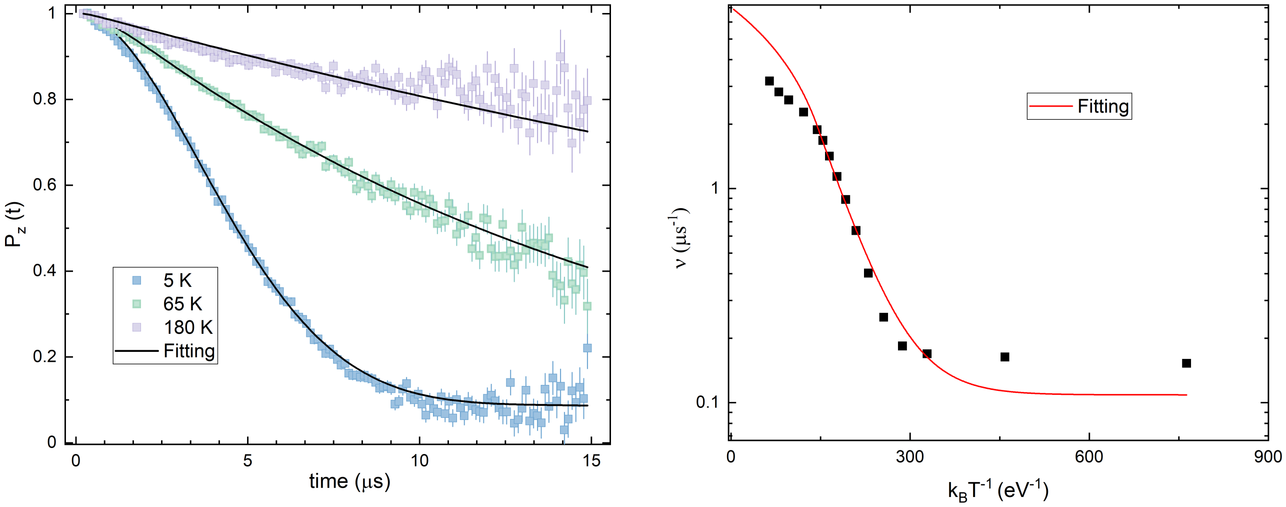

FIG. 17. Left: experimental data of $CsTi_3Bi_5$ at different temperature where black solid line the corresponding fit of the data using the dynamic Kubo-Toyabe expression. Right: hopping parameter ν, is plotted against the inverse temperature, along with its fit using eqn. 37.

$$\nu(T) = \nu_0 \exp\left(\frac{-E_a}{k_B T}\right) \tag{37}$$

where E_a is the activation energy and the estimated value is 8.0(2) meV.

Additional measurements have been performed in a longitudinal applied field, $B_0 = \frac{\omega_0}{2\pi\gamma_\mu} > 0$. The depolarization function $g_z(t)$ entering in Eq. 31 is modified according to

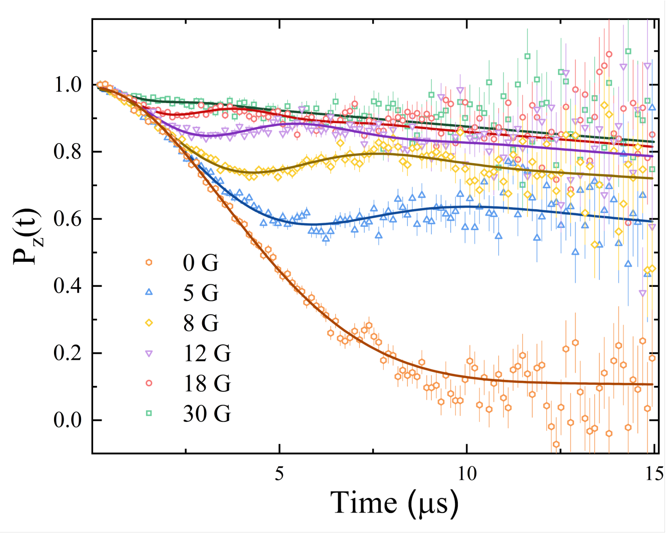

FIG. 18. Experimental data for longitudinal field μSR measurements performed at 5 K with applied fields up to 30 G. The solid lines show fits to the data using the dynamic Kubo–Toyabe model.

$$g_z(t) = A\left[1 - \frac{2\Delta^2}{\omega_0^2}\left(1 - \cos(\omega_0 t)\, e^{-\frac{1}{2}\Delta^2 t^2}\right) + \frac{2\Delta^4}{\omega_0^3}\int_0^t \sin(\omega_0 \tau)\, e^{-\frac{1}{2}\Delta^2\tau^2}\, d\tau\right] \quad (38)$$

and Eq. 35 is used to fit the experimental data with the hooping rate ν fixed to the reference value obtained from the ZF result, and using the nominal value for the externally applied longitudinal field. Figure 18 shows the results obtained at the lowest temperature for applied fields up to 30 G, along with the corresponding fitted spectra. The trend is consistent with the decoupling of quasistatic internal fields of the order of $\gamma_\mu\Delta \approx 2.5\,\mathrm{G}$, and a complete decoupling is indeed obtained for $B_{\mathrm{ext}} \geq 10\,\gamma_\mu\Delta$.

B. Muon localization

In order to better understand diffusion mechanisms in the system, we computed the solution to the Schrödinger equation for a muon in the unperturbed lattice. To achieve that, we discretized the muon Hamiltonian employing a finite-difference method with periodic boundary conditions. By using an orthorhombic supercell with lattice parameters $a = 11.739$ Å, $b = 10.166$ Å and $c = 18.556$ Å, the Potential Energy Surface (PES), i.e., the potential felt by the muon inside the crystal, has been sampled by placing the muon in a regular grid of $31 \times 31 \times 39$ points along the x, y and z directions respectively. Due to the symmetries of the crystal, this resulted in 830 irreducible (or independent) points that we evaluated using DFT as implemented within Quantum Espresso [13] (version 7.1). The 830 different DFT calculations have been performed using a wavefunction cutoff of 70 Ry, a smearing parameter equal to 0.01 Ry and a $4 \times 4 \times 3$ k-points grid. By relaxing the cell, we found that the best agreement between experimental data [14, 15] and simulations is obtained using a rVV10 non-local correlation functional [16]. The lowest eigenvalues and corresponding eigenvectors of the discretized 3-dimensional single-particle Hamiltonian have then been computed using the Arnoldi package [17] for large sparse matrices. The mass of the particle is $m_\mu = 206.768$

atomic units.

As it can be seen from Fig. 19a, the ground state corresponds to a muon wavefunction well localized between three Ti atoms and it is exactly aligned with the kagome plane along the c direction. The first excited state is instead slightly below and above the Ti plane, as shown in Fig. 19b. These results show that a localized ground state and a slightly more delocalized excited state exist and have non-overlapping wavefunctions. The two states are separated in energy by only 140 meV and are therefore both likely to be populated. A third eigenstate, shown in panel c) and 148 meV above the ground state, has instead a probability density that extends over both the previous sites. These results show indeed that a number of almost degenerate state exist in the kagome plane and can easily lead to muon diffusion as the temperature raises.

C. First principles description of muon sites

The refined position of muon sites in this system and the perturbation produced by the positive interstitial is computed using the approach described by Lancaster and Blundell [10] with the method and the code described in Ref. [11]. A 2x2x1 cell was initially used while refined simulations with a 4x4x2 supercell are performed for the two lowest energy sites. The results obtained after the structural relaxation are shown in table I and visualized in the unperturbed unit cell in Fig. 20. Notably, the two lowest energy sites correspond to the lowest energy eigenstates obtained from the solution of the 3D Schrödinger equation, indicating that self-trapping effects are rather limited in this system.

For each site, the perturbation induced by the muon on both the lattice structure and the electric field gradient (EFG) of neighboring atoms is computed and used to produce the polarization functions discussed in the next section. We note that the EFG at Bi sites is very large (see table II) and we therefore expect the Bi nuclei to be in the large quadrupole splitting regime. We also report that the potential along the $z \parallel c$ for site 1 is anharmonic but symmetric with respect to the kagome plane. This can also be appreciated in the results of Fig. 19. As a consequence, the expectation value for the position operator along the z is not altered by the anharmonicity of the potential nor it displays relevant temperature dependencies.

1. Polarization function

For each site, the muon polarization function is obtained by computing the time evolution of the muon spin according to the following Hamiltonian

$$\mathcal{H} = \mathcal{H}_{dip,i} + \mathcal{H}_{Q,i} \tag{39}$$

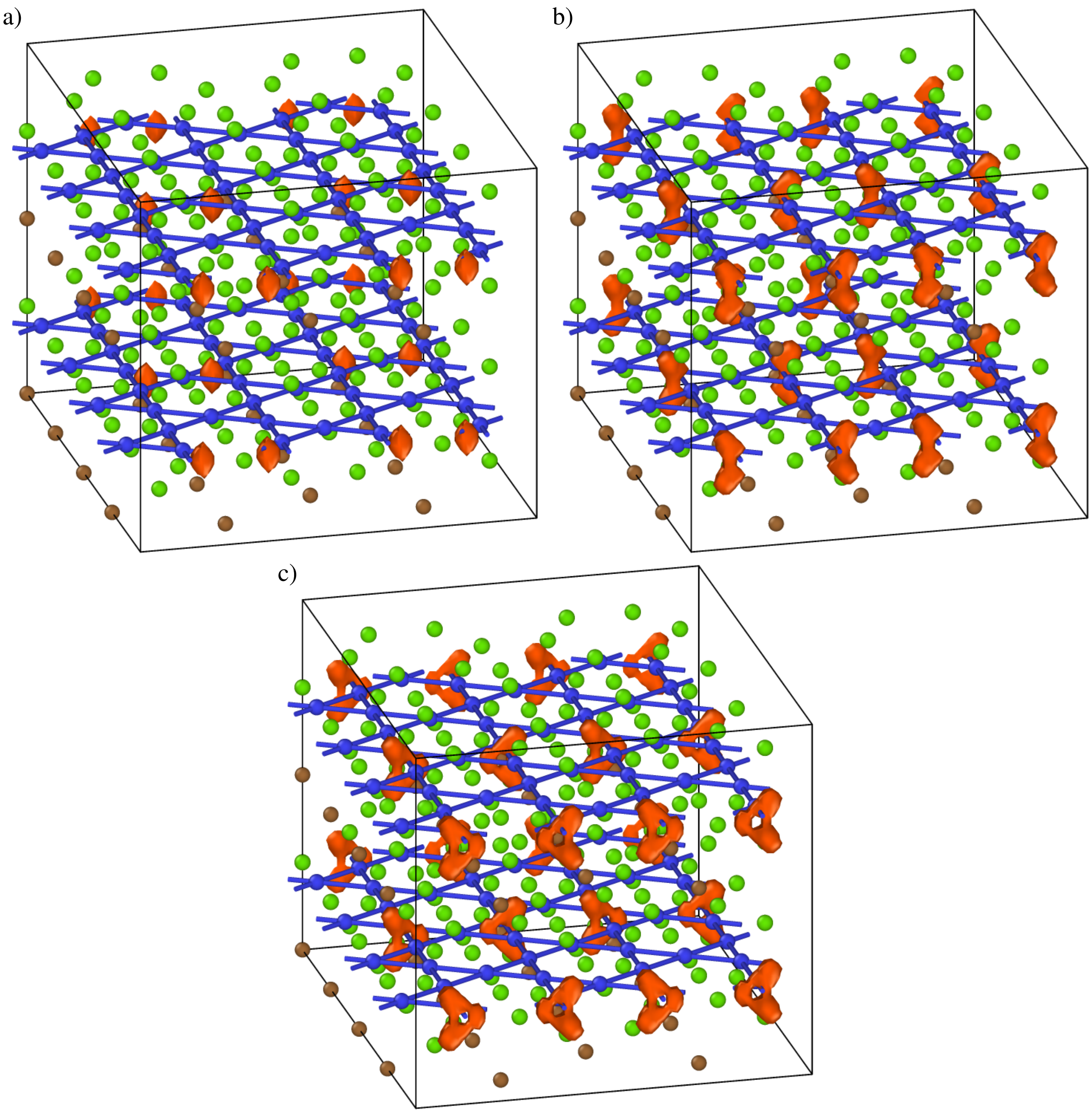

FIG. 19. Square modulus of the muon wavefunction in $CsTi_3Bi_5$ (with Ti as blue spheres, Cs brown and Bi green) for the lowest energy eigenstates. Only few states for each set of degenerate solutions (corresponding to equivalent positions in the lattice) are shown. The isosurface shows the value matching 1 % of the maximum probability density.

530 where

$$\mathcal{H}_{dip,i} = \frac{\mu_0 \hbar^2}{4\pi}\gamma_i\gamma_\mu\left(\frac{\mathbf{I}_i\cdot\mathbf{I}_\mu}{r^3} - \frac{3(\mathbf{I}_i\cdot\mathbf{r})(\mathbf{I}_\mu\cdot\mathbf{r})}{r^5}\right) \quad (40)$$

$$\mathcal{H}_{Q,i} = \frac{eQ_i}{6I_i(2I_i-1)}\sum_{\alpha,\beta\in\{x,y,z\}} V_i^{\alpha\beta}\left[\frac{3}{2}\left(I_i^\alpha I_i^\beta - I_i^\beta I_i^\alpha\right) - \delta_{\alpha\beta}I_i^2\right] \quad (41)$$

531 $\mathcal{H}_{dip,i}$ is the dipolar interaction between the muon and the nuclei accounting for the perturbation induced by the
532 muon on the lattice site and $\mathcal{H}_{Q,i}$ is the quadrupolar interaction between muon and nuclear spin, with V_i being the
533 EFG at nuclear site i. The remaining factors and constants should be clear from the context. The electric field

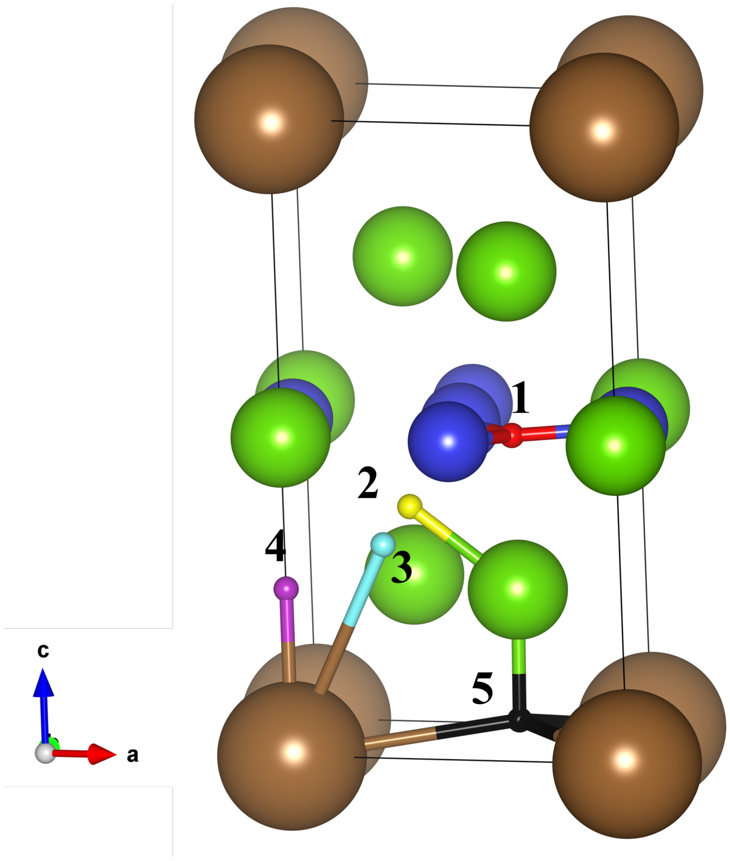

FIG. 20. Muon sites in $CsTi_3Bi_5$

Label	Position (frac. coord)	Energy difference (eV)	Distance from nn (Å)	Distance from nnn (Å)
1 - A	(0.332,0.667,0.5)	0	1.7538 (Ti)	2.5676 (Bi i)
2 - B	(0.372,0.184,0.384)	0.108	1.8961 (Ti)	3.3895 (Bi o)
3	(0.277,0, 0.342)	0.558	1.8649(Ti)	2.4984 (Bi o)
4	(0,0,0.270)	0.634	1.8454 (Bi o)	2.7068 (Cs)
5	(0.333,0.667,0.023)	1.034	2.0039 (Bi o)	3.4131 (Cs)

TABLE I. Total energy of the candidate muon sites found in $CsTi_3Bi_5$ along with its distance from nearest neighbor (nn) and next nearest neighbor (nnn). The two lowest energy sites are reported with letters A and B in the main text. The fraction coordinates and Wyckoff Symbol of other atoms are as Cs:(0,0,0; 1a), Ti (0.5,0.5,0.5;3g) and Bi i (0,0,0.5; 1b) and Bi o (0.333,0.667,0.249;4h).

gradient at the various nuclear sites is calculated with PAW pseudopotentials with the GIPAW code [18]. The values reported in Tab. II for the unperturbed structure are only slightly affected by the presence of the muon, generally by less than a factor 2.

The numerical solution to the time evolution of the muon spin is obtained with the approach proposed by Celio [19] as implemented in the UNDI code [20].

The convergence of the polarization function is limited by the large spin of Bi nuclei ($I = 9/2$). In our analysis

	$\|V_{zz}\|$	η
Bi1	6.73	0
Bi2	6.22	0
Ti	0.56	0.13
Cs	0.17	0

TABLE II. Unperturbed EFG in $CsTi_3Bi_5$. Values for V_{zz} are in atomic units, the conversion factor to SI is 9.717×10^{21} V/m^2.

we exclude Ti atoms, since only ∼15% of Ti nuclei have a (small) nuclear moment. Ignoring titanium, the set of nearest (nn) and next nearest neighbor (nnn) atoms includes 5 Bi for site 1 (or A) and 4 Bi for site 2 (B). A simple strategy to estimate how far is convergence for a given set of nuclei included in the simulation is using the second moment of the nuclear dipole field distribution, given by

$$\sigma_\infty^2 = \frac{2}{3}(\frac{\mu_0}{4\pi})^2\hbar^2\gamma_\mu^2 \sum_{j=1}^{N} \frac{\gamma_j^2 I_j(I_j+1)}{r_j^6} = \sigma_{incl}^2 + \sigma_{excl}^2 \quad (42)$$

where N is the total number of nuclei (including Ti and Cs isotopes) in the sample and we separate the contribution included in the numerical simulation, σ_{incl} from the total.

The square root of the ratio between the terms included in the simulation (σ_{incl}) and σ_∞ is 0.92 for site A and 0.94 for site B, thus showing that, despite the small set of neighbors included in the calculation, the result is less than 10% away from convergence. To further check this point we computed for sites 1 and 2 (A and B in the main text) the evolution of the muon spin considering 10 neighboring Bi atoms of the muon. The result is slightly improved, but is still not aligned with the experimental trend.

Surprisingly, none of the five interstitial sites shows good agreement with the experiment, as shown in Fig. 21. A very good agreement is obtained instead when site 2 is considered with a quenched EFG on Bi atoms, as shown in the same figure. The predicted polarization function works well at both short and long time, but the EFG value set on Bi atoms to produce this curve is 3 orders of magnitude smaller than the value predicted by density functional theory simulations. While such a small value is unrealistic, our results suggest a non-trivial interaction between the muon and the hosting electronic system whose description is beyond the scope of the current study.

The discrepancy between the predicted relaxation rate and the experimentally observed one is about 0.06 μs^{-1} and the missing contribution can be obtained with local magnetic fields at the muon site smaller than 0.2 mT. Even in the non-physical assumption that the relaxation rate Δ of the Kubo-Toyabe function (Eq. 36) is entirely of electronic origin, the magnetic field at the muon can be estimated from the maximum of the Maxwellian field distribution for producing the experimental relaxation rate. The result turns out to be 0.3 mT, which corresponds to local magnetic moments at the Ti sites smaller than one hundredth of Bohr magneton.

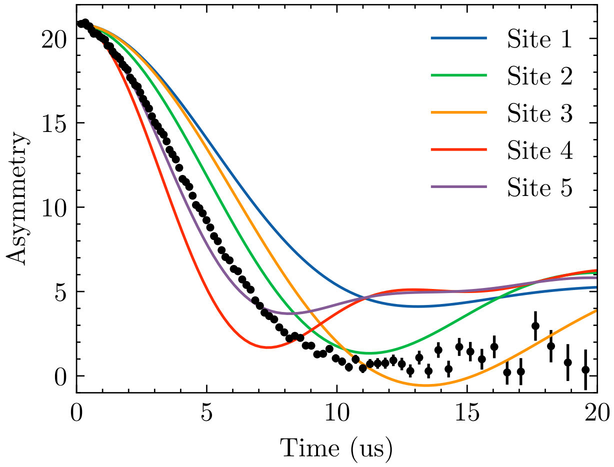

FIG. 21. Calculated polarization function obtained from first principles simulations.

D. Consistency between μSR and ARPES

We would like to point out that there is no inconsistency between the absence or very low amplitude of a static magnetic signal in μSR and the ARPES outcomes. We recall that we are examining states at a given position in momentum space and specifically at the center of the Brillouin zone. These states do not exhibit net spin and orbital moments – though they manifest sizable spin dichroism and non-standard single handedness spin-resolved response. The magnetic signal in μSR instead is given by the average over the entire Brillouin zone. Measurements of the dichroism across the whole Brillouin zone is consistently demonstrating that there is a net cancellation of the orbital moment – i.e. the dichroic amplitude is odd parity upon inversion of momentum. Regarding the fact that the loop current are marked by a charge circulation that may generate a magnetic field, we would like to point out that the proposed spin-orbital quadrupole loop current are not simple charged loop currents configurations. Indeed, they owe an internal helicity with plus and minus parity associated with the spin-orbital quadrupole structure– the components of the $\mathbf{L}\times\mathbf{S}$ operator, e.g. $(L_xS_y+L_yS_x)$, have pairs of eigenvalues with opposite sign. This implies that having a flow of a charge with a given spin-orbital quadrupole results into having electronic configurations with both charge current flow with helicity +1 together with an opposite counterflow associated with the spin-orbital quadrupole helicity -1. This aspect indicates that, although time-reversal symmetry is broken, there can be a vanishing net charge flow when considering spin-orbital quadrupole configurations of all the occupied states below the Fermi level. For instance, we have evaluated the expectation value of the charge flow within the unit cell, expressed by the sublattice current operator $M_z = \frac{1}{\sqrt{3}}(T_x + T_y + T_z)$, and it turns out to be vanishing. Consequently, the magnetic field associated to this type of loop current phase can be vanishing due to the cancelation of the contribution to the charge flow from the internal structure of the spin-orbital quadrupole. Due to this character of the spin-orbital quadrupole loop current states the absence of a static magnetic signal in μSR is not inconsistent with the examined phase. Moreover, there is no need to evaluate the magnetic field distribution at μ^+ stopping sites because the cancellation is not due to competing dipolar field arising from different magnetic atoms.

VIII. HOPPING PARAMETERS BETWEEN TI, CS, AND BI ATOMS

The following tables (from I to VII) report representative next-nearest-neighbor hopping parameters for Ti–Ti, Bi–Ti, and Cs–Ti hybridization. We explicitly report those for one Ti-Ti bond, the others are related by spatial symmetry transformations. One can notice that the hopping between Ti and Bi is generally larger than that between Ti and Cs. Indeed, Ti and Bi can hybridize as can be seen from the band structure. For the Ti–Bi hoppings, the dz^2 orbital was taken as a reference. Additionally, we note that Bi atoms 2, 3, 4, and 5 are all equivalent to each other (these are the Bi atoms outside the kagome plane).

Orbital	Ti_1	Ti_2	Ti_3
d_{xy}	3	13	23
d_{yz}	5	15	25
d_{z^2}	7	17	27
d_{xz}	9	19	29
$d_{x^2-y^2}$	11	21	31

TABLE III. Correspondence between Ti $3d$ orbitals and index numbers employed in the Tables II-VII for the orbital correspondence in the hopping amplitudes.

Orbitals	x	y	z	Re(Hopping)	Im(Hopping)
3–13	9.54	0	0	0.0111	0.00638
3–14	9.54	0	0	0	0
3–15	9.54	0	0	0	0
3–16	9.54	0	0	0.00217	0.00170
3–17	9.54	0	0	-0.0160	-0.00382
3–18	9.54	0	0	0	0
3–19	9.54	9.54	0	0	0
3–20	9.54	0	0	0.00117	0.00211
3–21	9.54	0	0	-0.0893	0.00218
3–22	9.54	0	0	0	0
4–13	9.54	0	0	0	0
4–14	9.54	0	0	0.0111	-0.00638
4–15	9.54	0	0	-0.00217	0.00170
4–16	9.54	0	0	0	0
4–17	9.54	0	0	0	0
4–18	9.54	0	0	-0.0160	0.00382
4–19	9.54	0	0	-0.00117	0.00211
4–20	9.54	0	0	0	0
4–21	9.54	0	0	0	0
4–22	9.54	0	0	-0.0893	-0.00218

TABLE IV. Orbital-dependent hopping amplitudes (real and imaginary parts) between Ti_1 and Ti_2 for second nearest-neighbor atoms. The coordinates (x, y, z) denote the connecting vector in Bohr units, and the hopping amplitudes are given in electron-volts (eV).

Orbitals	x	y	z	Re(Hopping)	Im(Hopping)
5–13	9.54	0	0	0	0
5–14	9.54	0	0	-0.00217	0.00170
5–15	9.54	0	0	0.000683	0.000435
5–16	9.54	0	0	0	0
5–17	9.54	0	0	0	0
5–18	9.54	0	0	-0.00215	0.000177
5–19	9.54	0	0	0.0187	0.00140
5–20	9.54	0	0	0	0
5–21	9.54	0	0	0	0
5–22	9.54	0	0	0.000533	-0.000378
6–13	9.54	0	0	0.00217	0.00170
6–14	9.54	0	0	0	0
6–15	9.54	0	0	0	0
6–16	9.54	0	0	0.000683	-0.000435
6–17	9.54	0	0	0.00215	0.000177
6–18	9.54	0	0	0	0
6–19	9.54	9.54	0	0	0
6–20	9.54	0	0	0.0187	-0.00140
6–21	9.54	0	0	-0.000533	-0.000378
6–22	9.54	0	0	0	0

TABLE V. Orbital-dependent hopping amplitudes (real and imaginary parts) between Ti_1 and Ti_2 for second nearest-neighbor atoms. The coordinates (x, y, z) denote the connecting vector in Bohr units, and the hopping amplitudes are given in electron-volts (eV).

Orbitals	x	y	z	Re(Hopping)	Im(Hopping)
7–13	9.54	0	0	0.0160	0.00382
7–14	9.54	0	0	0	0
7–15	9.54	0	0	0	0
7–16	9.54	0	0	-0.00215	-0.000177
7–17	9.54	0	0	-0.0129	-0.0000728
7–18	9.54	0	0	0	0
7–19	9.54	0	0	0	0
7–20	9.54	0	0	-0.00242	0.000472
7–21	9.54	0	0	-0.00479	0.000169
7–22	9.54	0	0	0	0
8–13	9.54	0	0	0	0
8–14	9.54	0	0	0.0160	-0.00382
8–15	9.54	0	0	0.00215	-0.000177
8–16	9.54	0	0	0	0
8–17	9.54	0	0	0	0
8–18	9.54	0	0	-0.0129	0.0000728
8–19	9.54	0	0	0.00242	0.000472
8–20	9.54	0	0	0	0
8–21	9.54	0	0	0	0
8–22	9.54	0	0	-0.00479	-0.000169

TABLE VI. Orbital-dependent hopping amplitudes (real and imaginary parts) between Ti_1 and Ti_2 for second nearest-neighbor atoms. The coordinates (x, y, z) denote the connecting vector in Bohr units, and the hopping amplitudes are given in electron-volts (eV).

Orbitals	x	y	z	Re(Hopping)	Im(Hopping)
9–13	9.54	0	0	0	0
9–14	9.54	0	0	0.00117	-0.00211
9–15	9.54	0	0	-0.0187	-0.00140
9–16	9.54	0	0	0	0
9–17	9.54	0	0	0	0
9–18	9.54	0	0	0.00242	0.000472
9–19	9.54	0	0	-0.0251	0.000679
9–20	9.54	0	0	0	0
9–21	9.54	0	0	0	0
9–22	9.54	0	0	0.00133	0.00156
10–13	9.54	0	0	-0.00117	-0.00211
10–14	9.54	0	0	0	0
10–15	9.54	0	0	0	0
10–16	9.54	0	0	-0.0187	0.00140
10–17	9.54	0	0	-0.00242	0.000472
10–18	9.54	0	0	0	0
10–19	9.54	0	0	0	0
10–20	9.54	0	0	-0.0251	-0.000679
10–21	9.54	0	0	-0.00133	0.00156
10–22	9.54	0	0	0	0

TABLE VII. Orbital-dependent hopping amplitudes (real and imaginary parts) between Ti_1 and Ti_2 for second nearest-neighbor atoms. The coordinates (x, y, z) denote the connecting vector in Bohr units, and the hopping amplitudes are given in electron-volts (eV).

Orbitals	x	y	z	Re(Hopping)	Im(Hopping)
11–13	9.54	0	0	0.0893	-0.00218
11–14	9.54	0	0	0	0
11–15	9.54	0	0	0	0
11–16	9.54	0	0	0.000533	0.000378
11–17	9.54	0	0	-0.00479	0.000169
11–18	9.54	0	0	0	0
11–19	9.54	0	0	0	0
11–20	9.54	0	0	-0.00133	0.00156
11–21	9.54	0	0	0.0100	-0.00266
11–22	9.54	0	0	0	0
12–13	9.54	0	0	0	0
12–14	9.54	0	0	0.0893	0.00218
12–15	9.54	0	0	-0.000533	0.000378
12–16	9.54	0	0	0	0
12–17	9.54	0	0	0	0
12–18	9.54	0	0	-0.00479	-0.000169
12–19	9.54	0	0	0.00133	0.00156
12–20	9.54	0	0	0	0
12–21	9.54	0	0	0	0
12–22	9.54	0	0	0.0100	0.00266

TABLE VIII. Orbital-dependent hopping amplitudes (real and imaginary parts) between Ti_1 and Ti_2 for second nearest-neighbor atoms. The coordinates (x, y, z) denote the connecting vector in Bohr units, and the hopping amplitudes are given in electron-volts (eV).

Orbital 1	Orbital 2	Hopping amplitude
Ti_1 dz2	Bi_1 6s	-0.512
Ti_1 dz2	Bi_2 6s	0.285
Ti_1 dz2	Bi_3 6s	0.285
Ti_1 dz2	Bi_4 6s	0.285
Ti_1 dz2	Bi_5 6s	0.285
Ti_1 dz2	Bi_1 px	-0.321
Ti_1 dz2	Bi_1 pz	-0.00231
Ti_1 dz2	Bi_1 py	0.556
Ti_1 dz2	Bi_2 px	-0.509
Ti_1 dz2	Bi_2 pz	-0.0614
Ti_1 dz2	Bi_2 py	-0.294
Cs 6s	Ti_1 dxy	0.0430
Cs 6s	Ti_1 dyz	0.0154
Cs 6s	Ti_1 dz2	-0.0727
Cs 6s	Ti_1 dxz	-0.0267
Cs 6s	Ti_1 dx2-y2	-0.0248

TABLE IX. Hopping parameters between Ti, Bi, and Cs orbitals for next-nearest-neighbor interactions. All amplitudes are given in electronvolts (eV).

[1] G. H. Wannier, The structure of electronic excitation levels in insulating crystals, Phys. Rev. **52**, 191 (1937).

[2] G. H. Wannier, Dynamics of band electrons in electric and magnetic fields, Rev. Mod. Phys. **34**, 645 (1962).

[3] K. Koepernik and H. Eschrig, Full-potential nonorthogonal local-orbital minimum-basis band-structure scheme, Phys. Rev. B **59**, 1743 (1999).

[4] I. Opahle, K. Koepernik, and H. Eschrig, Full-potential band-structure calculation of iron pyrite, Phys. Rev. B **60**, 14035 (1999).

[5] N. Marzari, A. A. Mostofi, J. R. Yates, I. Souza, and D. Vanderbilt, Maximally localized wannier functions: Theory and applications, Rev. Mod. Phys. **84**, 1419 (2012).

[6] G. Pizzi, V. Vitale, R. Arita, S. Blugel, F. Freimuth, G. Géranton, M. Gibertini, D. Gresch, C. Johnson, T. Koretsune, J. Ibanez-Azpiroz, H. Lee, J.-M. Lihm, D. Marchand, A. Marrazzo, Y. Mokrousov, J. I. Mustafa, Y. Nohara, Y. Nomura, L. Paulatto, S. Poncé', T. Ponweiser, J. Qiao, F. Thole, S. S. Tsirkin, M. Wierzbowska, N. Marzari, D. Vanderbilt, I. Souza,

A. A. Mostofi, and J. R. Yates, Wannier90 as a community code: new features and applications, Journal of Physics: Condensed Matter **32**, 165902 (2020).

[7] A. Hillier, J. Lord, K. Ishida, and C. Rogers, Muons at isis, Philosophical Transactions of the Royal Society A **377**, 20180064 (2019).

[8] A. D. Hillier, S. J. Blundell, I. McKenzie, I. Umegaki, L. Shu, J. A. Wright, T. Prokscha, F. Bert, K. Shimomura, A. Berlie, *et al.*, Muon spin spectroscopy, Nature Reviews Methods Primers **2**, 4 (2022).

[9] G. Solt, A generalized kubo-toyabe formula for muon spin relaxation in crystals with uniaxial symmetry, Hyperfine Interactions **96**, 167 (1995).

[10] S. J. Blundell and T. Lancaster, Dft+μ: Density functional theory for muon site determination, Applied Physics Reviews **10**, 021316 (2023).

[11] I. J. Onuorah, M. Bonacci, M. M. Isah, M. Mazzani, R. D. Renzi, G. Pizzi, and P. Bonfa', Automated computational workflows for muon spin spectroscopy (2024), arXiv:2408.16722 [physics.comp-ph].

[12] S. Blundell, R. De Renzi, T. Lancaster, and F. L. Pratt, *Muon spectroscopy: an introduction* (Oxford University Press, 2022).

[13] P. Giannozzi, O. Andreussi, T. Brumme, O. Bunau, M. B. Nardelli, M. Calandra, R. Car, C. Cavazzoni, D. Ceresoli, M. Cococcioni, N. Colonna, I. Carnimeo, A. D. Corso, S. de Gironcoli, P. Delugas, R. A. DiStasio, A. Ferretti, A. Floris, G. Fratesi, G. Fugallo, R. Gebauer, U. Gerstmann, F. Giustino, T. Gorni, J. Jia, M. Kawamura, H.-Y. Ko, A. Kokalj, E. Küçükbenli, M. Lazzeri, M. Marsili, N. Marzari, F. Mauri, N. L. Nguyen, H.-V. Nguyen, A. O. de-la Roza, L. Paulatto, S. Poncé, D. Rocca, R. Sabatini, B. Santra, M. Schlipf, A. P. Seitsonen, A. Smogunov, I. Timrov, T. Thonhauser, P. Umari, N. Vast, X. Wu, and S. Baroni, Advanced capabilities for materials modelling with Quantum ESPRESSO, Journal of Physics: Condensed Matter **29**, 465901 (2017).

[14] X. Chen, X. Liu, W. Xia, X. Mi, L. Zhong, K. Yang, L. Zhang, Y. Gan, Y. Liu, G. Wang, A. Wang, Y. Chai, J. Shen, X. Yang, Y. Guo, and M. He, Electrical and thermal transport properties of the kagome metals $a\mathrm{ti}_3\mathrm{bi}_5$ (a = Rb, Cs), Phys. Rev. B **107**, 174510 (2023).

[15] H. Yang, Y. Ye, Z. Zhao, J. Liu, X.-W. Yi, Y. Zhang, H. Xiao, J. Shi, J.-Y. You, Z. Huang, B. Wang, J. Wang, H. Guo, X. Lin, C. Shen, W. Zhou, H. Chen, X. Dong, G. Su, Z. Wang, and H.-J. Gao, Superconductivity and nematic order in a new titanium-based kagome metal csti3bi5 without charge density wave order, Nature Communications **15**, 9626 (2024).

[16] R. Sabatini, T. Gorni, and S. de Gironcoli, Nonlocal van der waals density functional made simple and efficient, Phys. Rev. B **87**, 041108 (2013).

[17] R. B. Lehoucq, D. C. Sorensen, and C. Yang, *ARPACK Users' Guide* (Society for Industrial and Applied Mathematics, 1998) https://epubs.siam.org/doi/pdf/10.1137/1.9780898719628.

[18] N. Varini, D. Ceresoli, L. Martin-Samos, I. Girotto, and C. Cavazzoni, Enhancement of dft-calculations at petascale: nuclear magnetic resonance, hybrid density functional theory and car–parrinello calculations, Computer Physics Communications **184**, 1827 (2013).

[19] M. Celio, New method to calculate the muon polarization function, Phys. Rev. Lett. **56**, 2720 (1986).
[20] P. Bonfà, J. Frassineti, M. M. Isah, I. J. Onuorah, and S. Sanna, Undi: An open-source library to simulate muon-nuclear interactions in solids, Computer Physics Communications **260**, 107719 (2021).
